# High-Efficiency Particulate Air Filters to Prevent Winter Respiratory Infections in Care Homes: The AFRI-c Cluster Randomized Clinical Trial

**DOI:** 10.1001/jamainternmed.2026.2199

**Published:** 2026-07-27

**Authors:** Alastair D. Hay, Rachel C. M. Brierley, Nick Turner, Joanna C. Thorn, Sophie Rees, Claire E. Brown, Laurel Campbell-Smith, Eleanor A. Gidman, Emily J. Henderson, Graziella Mazza, Gemma Morgan, Derren Ready, Craig Riddington, Jane Sprackman, Jodi Taylor, Tomas Welsh, Ruth Kipping

**Affiliations:** 1 Centre for Academic Primary Care, Bristol Medical School: Population Health Sciences, University of Bristol, Bristol, United Kingdom; 2 Bristol Trials Centre, Bristol Medical School: Population Health Sciences, University of Bristol, Bristol, United Kingdom; 3 Health Protection Operations, UK Health Security Agency, Bristol, United Kingdom; 4 Royal United Hospitals, Bath, United Kingdom; 5 Ageing and Movement Research Group, Bristol Medical School: Population Health Sciences, University of Bristol, Bristol, United Kingdom; 6 South Gloucestershire Council, Bristol, United Kingdom; 7 Public Health Microbiology Reference Service, UK Health Security Agency, London, United Kingdom; 8 Centre for Public Health, Bristol Medical School: Population Health Sciences, University of Bristol, Bristol, United Kingdom

## Abstract

**Importance:**

Portable high-efficiency particulate air (HEPA) filters effectively remove airborne microbes.

**Objective:**

To investigate whether HEPA filters reduce respiratory infection episodes in care home residents.

**Design, Setting, and Participants:**

This 2-arm cluster randomized clinical trial included care homes for older adults in England, with or without nursing and dementia care provision, and capacity for 20 or more residents in individual bedrooms. Data were collected between September 2021 and May 2024, with each care home participating for 1 winter.

**Interventions:**

Up to 5 HEPA filters for communal areas (clean air delivery rate set at 160 m^3^/h) and up to 16 filters for bedrooms (clean air delivery rate set at 60 m^3^/h). Both groups continued usual infection prevention and control measures.

**Main Outcomes and Measures:**

The primary outcome was respiratory infection rate per winter per bedroom resident (exposed to bedroom and communal room filters). Secondary outcomes included staff absenteeism. All outcomes were also explored for residents exposed only to communal room filters.

**Results:**

During the study period, 91 care homes were randomized, 47 to receive communal room filters, with 569 bedroom residents (median [IQR] age, 87 [81-92] years; 398 [70.0%] female), and 44 without communal room filters, with 589 residents without bedroom filters (median [IQR] age, 88 [82-92] years; 23 [71.8%] female). There was no evidence of a difference in the number of respiratory infections per winter per intervention vs control bedroom residents (0.99 vs 1.04; adjusted incident rate ratio, 0.92; 95% CI, 0.64-1.33; *P* = .67). There was also no evidence of a difference in the rates of staff absenteeism (adjusted incident rate ratio, 0.80 95% CI, 0.55-1.16; *P* = .24). Results were similar for the residents exposed only to communal room HEPA filters.

**Conclusions and Relevance:**

In this cluster randomized clinical trial, there was no difference in resident respiratory infection rates in care homes with HEPA filters in communal areas and bedrooms. Care homes should continue existing recommended prevention measures to control respiratory and other infections.

**Trial Registration:**

isrctn.org Identifier: ISRCTN63437172

## Key Points

**Question:**

Do portable high-efficiency particulate air filters in communal rooms and private bedrooms reduce respiratory (and other) infections in care home residents, as well as staff absenteeism?

**Findings:**

In this 2-arm cluster randomized clinical trial of 91 care homes with 1158 residents providing primary outcome data, there was no difference in the number of respiratory infections per winter per resident when care homes placed high-efficiency particulate air filters in communal rooms and private bedrooms. There was also no difference in the rates of other infections or staff absenteeism.

**Meaning:**

Care homes should continue using existing recommended prevention measures to control respiratory and other infections.

## Teaser

This randomized clinical trial investigates whether high-efficiency particulate air filters in communal rooms and private bedrooms reduce respiratory infection episodes in care home residents.

## Introduction

Respiratory and other infections are leading causes of morbidity^1^ and health service use worldwide in older adults.^2,3^ Globally, the proportion of people 65 years and older is rising faster than younger age groups, with the percentage expected to increase from 10% to 16% between 2022 and 2050.^4^ Around 10% of adults aged 65 to 85 years in the UK live in care homes^5^ and are particularly vulnerable to infections like COVID-19 and influenza.^6^ They are also twice as likely to receive an antibiotic prescription as their community peers,^7^ increasing their risk of antimicrobial-resistant infections, itself a major public health challenge.^8^

Infections are common and easily transmitted in care homes.^9^ Much has been done to develop models to explain and predict infection transmission. Prepandemic, the Wells-Riley model, developed to assess the risk of measles transmission,^10^ described 3 modes: direct (now scored by the World Health Organization [WHO] as contact), indirect (scored by WHO as direct deposition), and airborne. The WHO score takes account of SARS-CoV-2 transmission learning^11^ and further divides airborne into short- and long-range transmission. Short-range transmission occurs within 2 meters when infectious droplets are deposited onto mucous membranes or inhaled. Long-range transmission occurs beyond 2 meters, when smaller particles, which can remain airborne for several hours,^12^ are inhaled.

To our knowledge, there are no data regarding the relative contribution of these transmission modes. Existing infection prevention and control (IPC) methods focus on interrupting human contact (handwashing), direct deposition (cleaning), and short-range transmission (face coverings). However, long-range transmission remains largely unmitigated beyond improving ventilation where outdoor temperature and humidity permit.

Portable high-efficiency particulate air (HEPA) filters, developed to remove pollutants and pollen, trap particles that are 20 nm or smaller. Bacteria and viruses, including influenza viruses and SARS-CoV-2, are all smaller than 20 nm, suggesting that HEPA filters could prevent long-range transmission.^13^ One HEPA filter manufacturer demonstrated that its product can remove more than 99% of virus particles from a room-sized test chamber in 20 minutes.^14^ However, there is a paucity of evidence regarding the health benefits for care home residents. We therefore conducted the Air Filters to Prevent Respiratory Infections (Including COVID-19) in Care Homes (AFRI-c) cluster randomized clinical trial to investigate whether portable HEPA filters are effective in reducing respiratory and other infections in residents, as well as reducing staff absenteeism.

## Methods

### Design

This was a pragmatic, 2-arm, parallel, randomized (1:1) clinical trial clustered by residential care home because respiratory and gastrointestinal tract infections can spread quickly between residents and staff. The trial was designed in partnership with residents, care home staff, and members of the public. The trial protocol is available in Supplement 1.

Ethical approval for this trial was given by London–Harrow National Health Service Research Ethics Committee, and participants provided informed consent (or advice from consultees). The study followed Consolidated Standards of Reporting Trials (CONSORT) reporting guidelines.

### Recruitment, Randomization, and Masking

**Figure 1.**
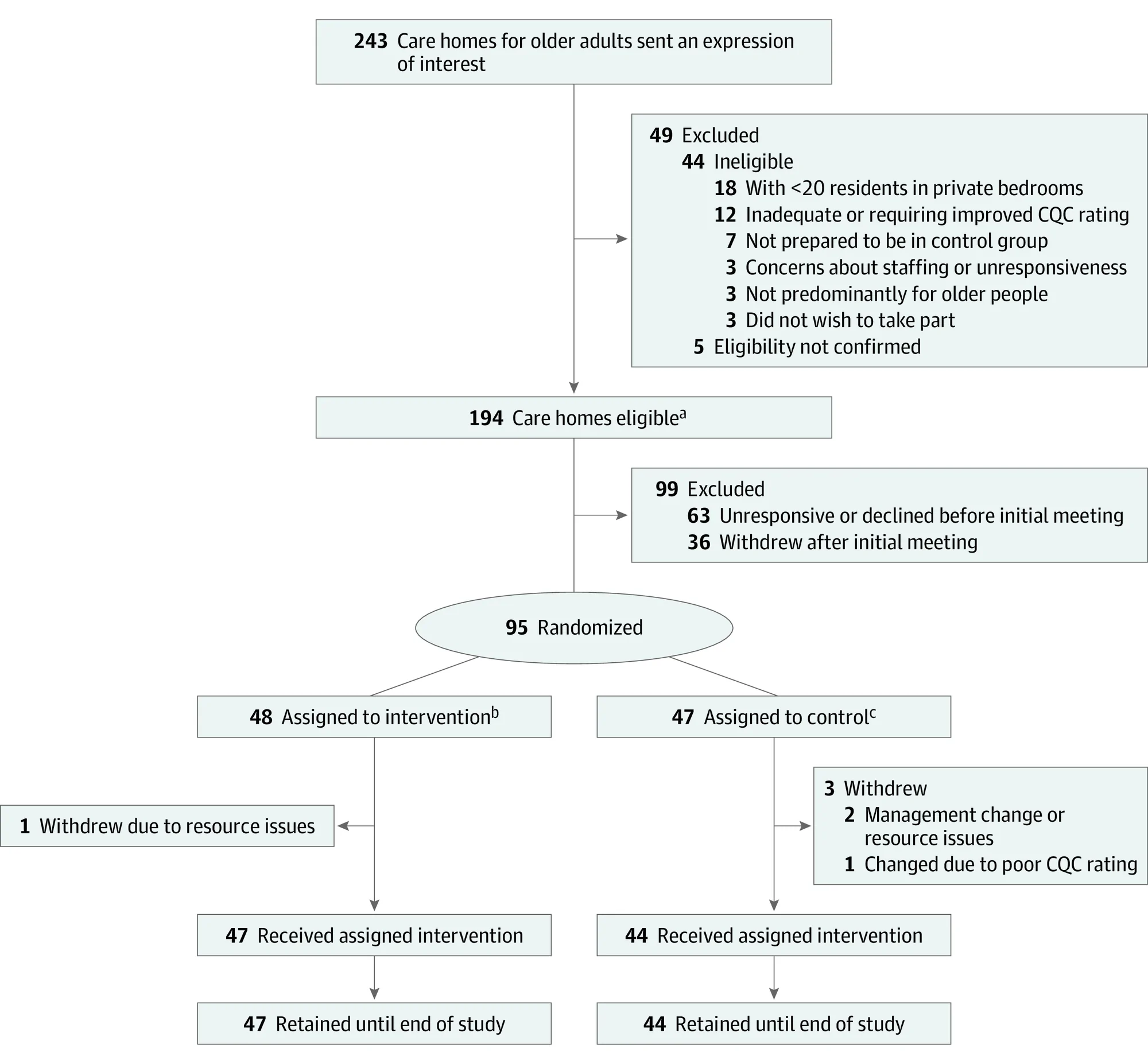
CONSORT Flow Diagram for Care Homes CQC indicates UK Care Quality Commission. ^a^Capacity for 20 or more residents in single bedrooms, willing to place high-efficiency particulate air filters in communal rooms, and willing to remove existing high-efficiency particulate air filters. ^b^Intervention care homes sought resident consent for bedroom high-efficiency particulate air filters, to complete questionnaires, and to provide data from National Health Service records (see Figure 2). ^c^Control care homes sought resident consent to complete questionnaires and provide data from National Health Service records (see Figure 2).

Care homes in England were identified, recruited, and followed up with the support of the National Institute for Health and Care Research’s ENRICH (Enabling Research in Care Homes) network. A detailed description of recruitment has been published,^15^ but in summary, care homes were eligible if they had capacity for 20 or more older residents in single bedrooms, were willing to place HEPA filters in communal rooms, and were willing to remove any existing HEPA filters. They were excluded if rated as inadequate or requiring improvements by the UK Care Quality Commission.^16^ Once the owner or manager had provided written agreement and information on care home baseline characteristics, care homes were randomized to receive communal room HEPA filters (intervention) or continue with usual infection, prevention, and control measures (control) (Figure 1). Randomization occurred between September 2021 and May 2024, with each care home participating for 1 winter.

Randomization lists were generated using the Stata command *ralloc *(StataCorp), conducted by a statistician (N.T.) with no involvement in recruitment and concealed from trial operations staff who were involved in recruitment. Randomization was stratified by the Index of Multiple Deprivation decile (categorized as 1-3, 4-7, or 8-10) of the geographical area (postcode) of the care home and provision of nursing care (yes/no) using randomly permuted blocks of size 2 and 4. Care home staff and residents, and research team members visiting and interacting with the care homes, were unmasked. The trial statistician (E.A.G.) was also unmasked, to prepare reports for review by members of the data monitoring committee. Other investigators remained unaware of care home allocation.

**Figure 2.**
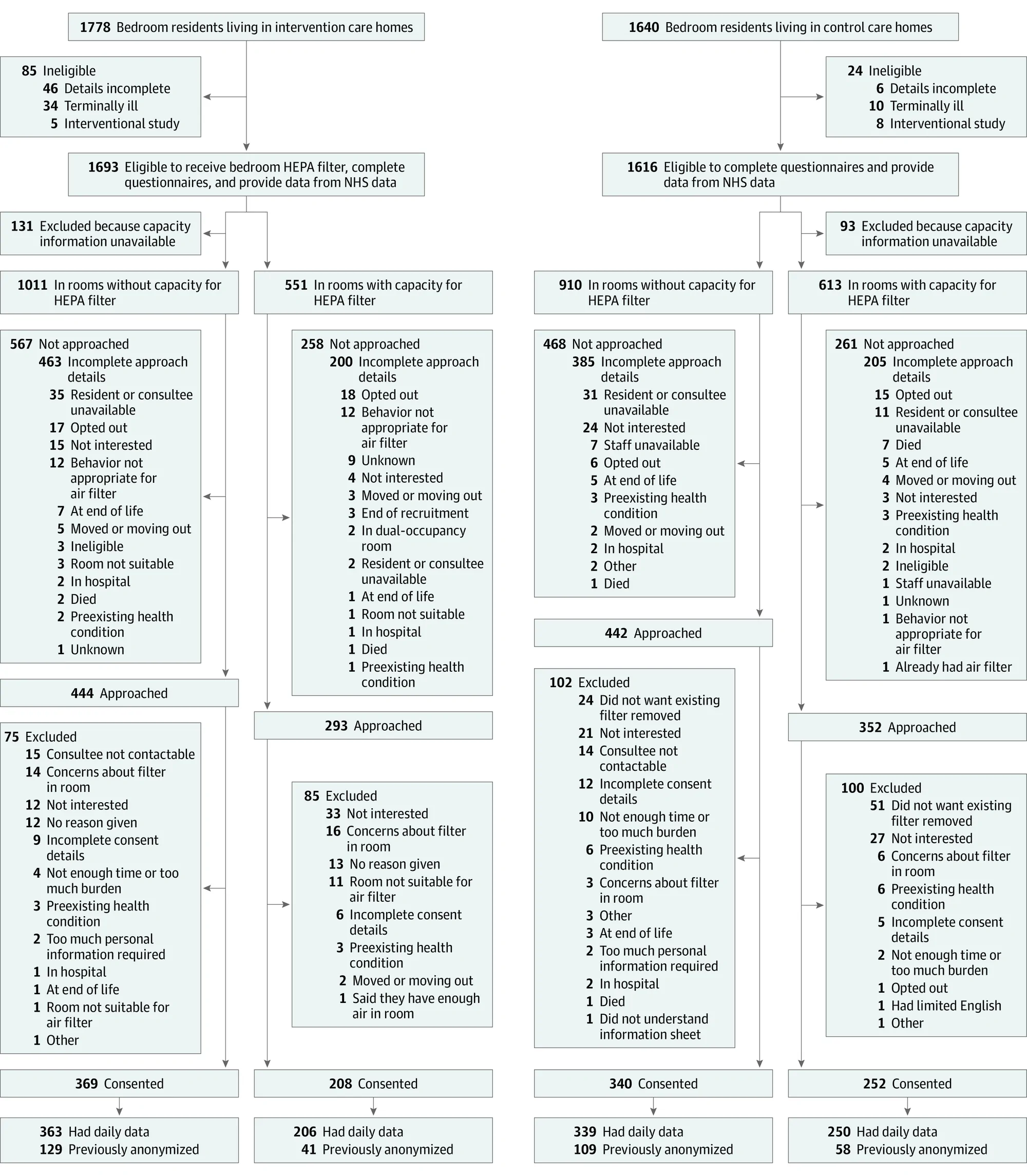
CONSORT Flow Diagram for Residents in Primary Outcome HEPA indicates high-efficiency particulate air; NHS, National Health Service.

Care home residents were then assessed for eligibility. They were included if expected to reside in a single-occupancy bedroom (the UK norm) for at least 1 month and excluded if expected to die within 7 days or were participating in a conflicting study (Figure 2). Informed consent (or advice from consultees) was sought by a member of the research or ENRICH team until up to 16 residents agreed to (1) have a HEPA filter in their bedroom (only for residents in intervention care homes), (2) to complete questionnaires, and (3) for information regarding National Health Service treatments to be extracted from primary care medical records.

From here on, these participants who gave consent are referred to as bedroom residents (eTable 2 in Supplement 2). The remaining residents, for whom only anonymized daily infection data were collected (and who did not receive a bedroom air filter), are from here on referred to as communal room residents (eTable 2 in Supplement 2) because these participants were exposed only to communal room filters if residing in an intervention care home (or no HEPA filter use if residing in a control care home).

### Intervention

Philips HEPA filters were selected by researchers, care home managers, and residents as suitable for the setting. Similarly, in discussion with Philips and public representatives, the number of HEPA filters, and filter settings, were selected to balance clean air delivery rate (CADR) with acceptability to residents (noise and air flow cooling). Care home floor plans and communal room dimensions were submitted by intervention care homes such that up to 5 AC3033 units could be distributed between the most frequently used resident and staff communal rooms to ensure a CADR of 160 m^3^/h on setting 1 (eFigure 1 in Supplement 2). One AC2936/33 HEPA filter was provided for each of up to 16 intervention residents, set on the quietest sleep setting, providing a CADR of 60 m^3^/h. Staff were provided with manufacturer guidance about how to maximize HEPA filter efficiency and with labels to remind staff and all residents to keep all units switched on. Staff in both groups were advised to continue IPC measures as usual.

### Outcomes and Analytic Methods

All outcomes were prespecified in the protocol,^15^ statistical analysis plan (Supplement 1), and eTable 1 in Supplement 2. The primary outcome was the number of bedroom resident respiratory infection episodes, derived from new (or worsening of preexisting) objective respiratory symptoms: runny/blocked nose, sneezing, runny ear, red/sticky eye(s) (considered part of the respiratory tract due to the lacrimal duct), hoarse voice, cough, wheeze, noisy breathing, or sputum. Shortness of breath was also included as an objective respiratory symptom but when present in isolation did not contribute to the onset or end of a respiratory infection episode. Symptoms were collected using electronic versions (eFigures 2 and 3 in Supplement 2) of a previously validated symptom diary,^17^ used in multiple previous studies.^18,19^ The eMethods in Supplement 2 describe how episodes were defined and how sensitivity analysis outcomes were defined.

Care home staff were asked to record sickness absences, time off work, and type of infection where an absence was due to an infection (eMethods in Supplement 2). A primary care medical record review was conducted to record medically diagnosed infections, antibiotic prescribing and hospitalization, and UK Health Security Agency data used to record care home infection outbreaks and microbiologically confirmed infections.

The primary analysis compared the effect of HEPA filters in communal rooms and private bedrooms on respiratory infection episodes in bedroom residents. Secondary analyses included a comparison of the effect of HEPA filters in communal rooms on respiratory infection episodes in communal room residents. Sample-size calculation details and a full description of the analytic methods are provided in the eMethods in Supplement 2.

### Statistical Analysis

Results are presented as treatment effects with 95% CIs. Analyses were performed using Stata, version 18.0 (StataCorp) and SAS, version 9.4 (SAS Institute). *P* < .05 was considered statistically significant.

## Results

### Care Homes

Expressions of interest were received from 243 care homes, of which 144 were considered eligible and 95 randomized (Figure 1). Four care homes subsequently withdrew (1 in intervention and 3 in control), leaving 91 care homes (47 intervention and 44 control), all of which completed the study. They were similar with respect to key characteristics, including architectural type, UK Care Quality Commission rating, ownership, provision of dementia and nursing care, number of communal rooms, and the very high proportion of residents residing in single rooms (Table 1).

**Table 1.** Baseline Characteristics of Care Homes

Characteristic	No. (%)		
	Intervention (n = 44)	Control (n = 47)	Overall (N = 91)
Winter randomized			
First	6 (13.6)	4 (8.5)	10 (11.0)
Second	17 (38.6)	18 (38.3)	35 (38.5)
Third	21 (47.7)	25 (53.2)	46 (50.5)
IMD decile			
1-3	10 (22.7)	8 (17.0)	18 (19.8)
4-7	19 (43.2)	22 (46.8)	41 (45.1)
8-10	15 (34.1)	17 (36.2)	32 (35.2)
CQC inspection rating			
Outstanding	3 (6.8)	6 (12.8)	9 (9.9)
Good	41 (93.2)	41 (87.2)	82 (90.1)
Building classification			
Stuart/Jacobean	1 (2.3)	0	1 (1.1)
Georgian	2 (4.6)	2 (4.3)	4 (4.4)
Regency	1 (2.3)	2 (4.3)	3 (3.3)
Victorian	10 (22.7)	8 (17.0)	18 (19.8)
Queen Anne/Domestic revival	2 (4.6)	1 (2.1)	3 (3.3)
Arts and Crafts	1 (2.3)	2 (4.3)	3 (3.3)
Edwardian	2 (4.6)	1 (2.1)	3 (3.3)
Addison	0	2 (4.3)	2 (2.2)
Art Deco	1 (2.3)	0	1 (1.1)
1930s	0	1 (2.1)	1 (1.1)
1950s	1 (2.3)	2 (4.3)	3 (3.3)
1960s and 1970s	1 (2.3)	1 (2.1)	2 (2.2)
1980 to current	22 (55.0)	25 (53.2)	47 (51.6)
Part of a care home chain^a^	28 (63.6)	22 (46.8)	50 (54.9)
Provides nursing care^a^	19 (43.2)	21 (44.7)	40 (44.0)
Provides dementia care^a^	38 (86.4)	41 (87.2)	79 (86.8)
Maximum capacity, median (IQR)^a^	44 (32-62)	36 (28-52)	38 (30-58)
Total No. of residents in all care homes^a^	1736	1757	3493
Total occupancy, median (IQR)^a^	38 (30-56)	31 (24-43)	35 (27-49)
Residents in single rooms, No./total No. (%)^b^	1225/1246 (98.3)	1654/1678 (98.6)	2879/2924 (98.5)
Residents with dementia, No./total No. (%)^a^	522/1736 (30.1)	742/1757 (42.2)	1264/3493 (36.2)
Residents receiving nursing care, No./total No. (%)^a^	958/1736 (55.2)	907/1757 (51.6)	1865/3493 (53.4)
No. of communal rooms^a^			
Staff only, median (IQR)	2 (1-3)	2 (1-2)	2 (1-3)
Resident only, median (IQR)	4 (3-6)	4 (3-5)	4 (3-5)
Already using air filter device^c^	2 (4.5)	3 (6.4)	5 (5.5)
Total permanent staff, median (IQR)	48 (38-66)	51 (35-72)	49 (36-68)
Full-time permanent staff, median (IQR), %	63 (54-81)	57 (46-75)	60 (49-80)
Total agency staff, median (IQR)	5 (2-6)	13 (5-19)	6 (2-8)
Window policy			
Open	1 (2.3)	2 (4.3)	3 (3.3)
Closed	2 (4.5)	3 (6.4)	5 (5.5)
No policy	41 (93.2)	42 (89.4)	83 (91.2)
Door policy			
Open	4 (9.1)	7 (14.9)	11 (12.1)
Closed	1 (2.3)	3 (6.4)	4 (4.4)
No policy	39 (88.6)	37 (78.7)	76 (83.5)
Has IPC policy	44 (100)	47 (100)	91 (100)

Abbreviations: CQC, UK Care Quality Commission; IMD, Index of Multiple Deprivation; IPC, infection prevention and control.

^a^ All data collected at care home screening (expression of interest).

^b^ Denominators differ due to some care homes having incomplete single-occupancy data.

^c^ Care homes asked to switch off or remove existing filters. Any not willing were excluded.

### Bedroom Residents

A total of 1169 bedroom residents consented, of whom 713 (61.0%) did not have capacity (Figure 2). After excluding bedroom residents for whom no daily data were collected (eTable 13 in Supplement 2), 1158 residents (569 in intervention and 589 in control) contributed 201 319 valid follow-up days to the primary analysis (6787 [3.4%] in the first winter 1, 63 198 [31.4%] in the second winter, and 130 884 [65.0%] in the third winter). Intervention and control group bedroom residents were similar with respect to key characteristics including sex (398 [70.0%] female vs 423 [71.8%] female), age (median [IQR], 87 [81-92] years vs 88 [82-92] years), receiving nursing care (199 [35.0%] vs 253 [43.0%]), dementia diagnosis (333 [58.5%] vs 332 [56.4%]), and Rockwood frailty score (median [IQR], 6 [4-7] vs 6 [5-7]) (Table 2).

**Table 2.** Baseline Characteristics of Bedroom Residents^a^

Characteristic	No./total No. (%)^b^		
	Intervention (n = 569)	Control (n = 589)	Overall (N = 1158)
Winter randomized, No. (%)			
First	62 (10.9)	38 (6.5)	100 (8.6)
Second	197 (34.6)	191 (32.4)	388 (33.5)
Third	310 (54.5)	360 (61.1)	670 (57.9)
Sex, No. (%)			
Female	398 (70.0)	23 (71.8)	821 (70.9)
Male	171 (30.1)	166 (28.2)	337 (29.1)
Age, median (IQR), y	87 (81-92)	88 (82-92)	88 (82-92)
Race and ethnicity^c^			
Asian or Asian British	0/568	1/589 (0.2)	1/1157 (0.1)
Black, African, Caribbean, or Black British	3/568 (0.5)	6/589 (1.0)	9/1157 (0.8)
White or Caucasian	564/568 (99.3)	582/589 (98.8)	1146/1157 (99.0)
Other groups	1/568 (0.2)	0/589	1/1157 (0.1)
Receives nursing care	199/568 (35.0)	253/589 (43.0)	452/1157 (39.1)
Dementia diagnosis^d^	333 (58.5)	332 (56.4)	665 (57.4)
Frailty score, median (IQR)^d^	6 (4-7)	6 (5-7)	6 (4-7)
Received influenza vaccine^d^	538 (94.6)	554 (94.1)	1092 (94.3)
Received COVID-19 vaccine^d^	553/569 (97.2)	562/588 (95.6)	1115/1157 (96.4)
Belief that infections can spread through the air^d^			
Strongly disagree	23/475 (4.8)	28/515 (5.4)	51/990 (5.2)
Slightly disagree	14/475 (2.9)	8/515 (1.6)	22/990 (2.2)
Not sure	34/475 (7.2)	22/515 (4.3)	56/990 (5.7)
Slightly agree	55/475 (11.6)	73/515 (14.2)	128/990 (12.9)
Strongly agree	347/475 (73.1)	384/515 (74.6)	731/990 (73.8)
Belief that HEPA filters can reduce infections^d^			
Strongly disagree	5/475 (1.1)	18/515 (3.5)	23/990 (2.3)
Slightly disagree	15/475 (3.2)	5/515 (1.0)	20/990 (2.0)
Not sure	214/475 (45.1)	235/515 (45.6)	449/990 (45.4)
Slightly agree	130/475 (27.4)	147/515 (28.5)	277/990 (28.0)
Strongly agree	112/475 (23.6)	110/515 (21.4)	222/990 (22.4)
Sleep quality^d^			
Very satisfied	106/475 (22.3)	149/515 (28.9)	255/990 (25.8)
Satisfied	204/475 (42.9)	221/515 (42.9)	425/990 (42.9)
Not sure	104/475 (21.9)	83/515 (16.1)	187/990 (18.9)
Dissatisfied	33/475 (6.9)	45/515 (8.7)	78/990 (7.9)
Very dissatisfied	22/475 (4.6)	14/515 (2.7)	36/990 (3.6)
Care home temperature^d^			
Very satisfied	185/475 (38.9)	204/515 (39.6)	389/990 (39.3)
Satisfied	253/475 (53.3)	275/515 (53.4)	528/990 (53.3)
Not sure	25/475 (5.3)	11/515 (2.1)	36/990 (3.6)
Dissatisfied	16/475 (3.4)	25/515 (4.9)	41/990 (4.1)
Very dissatisfied	1/475 (0.2)	3/515 (0.6)	4/990 (0.4)
Care home odors^d^			
Very satisfied	183/475 (38.5)	239/515 (46.4)	422/990 (42.6)
Satisfied	246/475 (51.8)	240/515 (46.6)	486/990 (49.1)
Not sure	31/475 (6.5)	27/515 (5.2)	58/990 (5.9)
Dissatisfied	14/475 (2.9)	6/515 (1.2)	20/990 (2.0)
Very dissatisfied	1/475 (0.2)	3/515 (0.6)	4/990 (0.4)
Care home air quality^d^			
Very satisfied	129/475 (27.2)	136/515 (26.4)	265/990 (26.8)
Satisfied	263/475 (55.4)	286/515 (55.5)	549/990 (55.5)
Not sure	68/475 (14.3)	87/515 (16.9)	155/990 (15.7)
Dissatisfied	15/475 (3.2)	6/515 (1.2)	21/990 (2.1)
Very dissatisfied	0/475	0/515	0/990
Communal room visiting frequency			
>2 d/wk	124/475 (26.1)	125/515 (24.3)	249/990 (25.2)
2-4 d/wk	51/475 (10.7)	55/515 (10.7)	106/990 (10.7)
>4 d/wk	302/475 (63.6)	335/515 (65.0)	637/990 (64.3)

Abbreviation: HEPA, high-efficiency particulate air.

^a^ Residents in intervention care homes were exposed to HEPA filters in their private bedrooms and communal rooms (and their control group counterparts).

^b^ Difference between denominators are the numbers of missing values for the respective measurement. Unless otherwise stated, all data were collected at resident baseline (consent). The number of consented residents includes all residents consented over the winter periods who had valid daily data collection.

^c^ Race and ethnicity were self-reported by participants, including other groups.

^d^ Collected at resident screening (registry).

Intervention bedroom residents experienced 390 respiratory infection episodes during 95 235 at-risk days (0.99 per bedroom resident per winter), while control bedroom residents experienced 442 episodes in 102 579 days (1.04 per bedroom resident per winter) (Table 3 and eFigure 4 in Supplement 2). There was no evidence of a difference between intervention and control groups (adjusted incident rate ratio [aIRR], 0.92; 95% CI, 0.64-1.33; *P* = .67). This result was unchanged by the sensitivity analyses (Table 3). There was also no evidence of treatment effect heterogeneity for care homes providing nursing care or any resident subgroups (eTable 5 in Supplement 2).

**Table 3.** Primary Outcome and Sensitivity Analyses Among Bedroom Residents

Variable	Intervention			Control			aIRR (95% CI)^c^	*P* value
	No. of episodes	Risk days^a^	Rate per resident per winter^b^	No. of episodes	Risk days^a^	Rate per resident per winter^b^		
Primary outcome: No. of resident respiratory infection episodes (objective symptoms)^d^	390	95 235	0.99	442	102 579	1.04	0.92 (0.64-1.33)	.67
Sensitivity analysis 1: primary outcome plus presence of fever^e^	9	97 376	0.02	15	105 032	0.03	0.59 (0.19-1.81)	.36
Sensitivity analysis 2: primary outcome plus presence of physical and/or mental decline	84	97 053	0.21	124	104 622	0.29	0.67 (0.41-1.10)	.11
Sensitivity analysis 3: primary outcome plus subjective symptoms^f^	157	35 848	1.06	206	45 254	1.10	0.90 (0.56-1.45)	.67

Abbreviation: aIRR, adjusted incident rate ratio.

^a^ Number of risk days is defined as the number of valid days, minus the number of episode days, minus 2 days (that define episode end), plus 1 person-day to ensure risk above 0.

^b^ Number of symptomatic winter respiratory infection episodes per 242 resident risk days.

^c^ Adjusted for nursing care provision, deprivation tertile, and winter.

^d^ Derived from daily data collection where staff were asked to report new (or worsening of preexisting) objective respiratory symptoms (eg, runny/blocked nose, sneezing, cough, and wheeze or sputum) reported daily by staff between September 1 and May 31. Infection episode start was defined as the onset of 2 new (worsening) respiratory symptoms for at least 1 day, or 1 respiratory symptom for at least 2 days, and the end as the last symptomatic day preceding 2 asymptomatic days.

^e^ Defined as temperature measured at 37.8 °C or higher.

^f^ Symptoms include sore/tickly throat, earache, and change in taste or smell. Collection was restricted to residents with capacity.

There was also no evidence of a difference in any of the secondary outcomes (eTable 6 in Supplement 2). Intervention arm compliance was very high, both for binary (96% of residents had HEPA filter in use at ≥20% of checks) and continuous (percentage of days HEPA filter was in use: median [IQR], 97% [89%-98%]) measures of compliance. Repeating the primary outcome analysis based on compliance status was consistent with the primary analysis model (eTable 6 in Supplement 2). Completion of daily data recording was similarly very high (completion of valid daily symptom diary days was 97%) (eTable 7 in Supplement 2).

### Communal Room Residents

Valid daily symptom data were provided for 1936 communal room residents, 1004 in intervention care homes and 932 in control care homes, contributing 258 286 follow-up days. The intervention and control group communal room residents were also similar with respect to age (median [IQR], 87 [81-92] years vs 87 [81-92] years), dementia diagnosis (670 [66.7%] vs 567 [60.8%]), and Rockwood frailty scores (median [IQR], 6 [4-7] vs 6 [5-7]) (eTable 8 in Supplement 2).

There was no evidence of differences in the number of respiratory infection episodes, symptomatic respiratory infection days, gastrointestinal tract infections (episodes or symptomatic days), episodes of fever and/or delirium, or falls/near falls. There was evidence of a reduction in the number of symptomatic days with fever and/or delirium and/or acute deterioration in physical ability (aIRR, 0.49; 95% CI, 0.26-0.90, *P* = .02) and number of days antibiotics were taken (aIRR, 0.57; 95% CI, 0.37-0.87; *P* = .01) in intervention communal room residents (eTable 9 in Supplement 2). Intervention arm compliance was very high, both for binary (96% of communal room HEPA filters were in use at ≥20% of checks) and continuous (percentage of days HEPA filter was in use: median [IQR], 96% [87%-98%]) measures of compliance (eTable 10 in Supplement 2).

### Staff Absenteeism and Infection Outbreaks

There was no evidence of a difference between intervention and control care homes in the number of staff working days lost to illness or infections (aIRR, 0.80; 95% CI, 0.55-1.16; *P* = .24; eTable 11 in Supplement 2) or the number of microbiologically confirmed care home infection outbreaks (eTable 12 in Supplement 2).

### Potential Costs Avoided

A HEPA filter like those used in the AFRI-c trial currently costs around £240 (US $323).^20^ Electrical running costs are estimated to be around £27 (US $36) per year,^21^ and manufacturer documentation suggests the internal filter needs to be replaced every 12 months at £62 (US $83) each.^20^ Assuming each care home uses 15 filters, the costs of implementing air filters over the first 5 years would be approximately £10 275 (US $13 830) per care home. With approximately 15 000 care homes in England, the budget impact could be as much as £154 million (US $207 million) if HEPA filters were implemented in every care home.

## Discussion

This robust pragmatic trial found no evidence that placing portable HEPA filters in bedrooms and communal rooms reduced respiratory or other infections in care home residents. We also found no evidence of overall effects on falls, antibiotic use, hospital admissions, care home infection outbreaks, care home staff absenteeism, or perceived care home air quality.

### Results in Context of Previous Research

There is clear evidence that HEPA filters are capable of cleansing the air of pollutants,^22^ allergens,^23^ and microbes^14,24^; that they can improve quality of life^22^; and that clean air increases longevity.^25^ A small, individually randomized crossover trial found some possible signals of HEPA filter effectiveness in reducing respiratory infections in care home residents but appeared underpowered.^26^ The present trial is larger, randomized at the care-home level, and provides evidence consistent with 2 systematic reviews, which concluded that portable HEPA filters do not reduce respiratory infection acquisition in private homes and offices.^27,28^

### Implications

There is currently insufficient evidence to support managers, residents, and local authorities investing in HEPA filters to supplement existing infection-prevention measures to further reduce respiratory infections in care home residents. Care homes should maintain non–air filtration IPC measures, including ventilation, surface cleaning, handwashing, personal protective equipment, and promoting vaccination. Potential cost savings accrued by care homes in England not purchasing HEPA filters are estimated to be £30 million (US $40 million) per annum.

### Strengths and Limitations

To our knowledge, this is the first pragmatic, cluster randomized clinical trial to investigate the health benefits of HEPA filters in care home residents in single-occupancy rooms (the UK norm), a group known to be vulnerable to infections. The effect sizes we report are likely to reflect what could be expected if a similar number of portable HEPA filters are placed in care homes. The study care home and resident populations are comparable to UK care homes^5^ and residents^5^ in terms of the percentage providing nursing care (44% vs 50%), median age (88 vs 86 years), female sex (71% vs 70%), and White race (99% vs 98%), due to study teams maximizing nonselective resident invitation.

We considered 6 explanations for the observed null result. First, treatment allocation was unmasked. If staff beliefs that filters were effective led to reduced reporting of respiratory infection episodes, it might also have led to shorter illness reporting, which was not observed. In any case, we decided against using sham units in the control group because without an internal fan, group allocation could have been determined by the absence of air flow and motor sounds, and with an internal fan, unfiltered air would have been circulated, which could increase infection rates in the control group, leading to a false positive or exaggerated evidence of effectiveness.

Second, data collection started during the COVID-19 pandemic when additional care home IPC interventions, such as use of personal protective equipment, resident isolation, closing care homes to visitors, preventing contact with symptomatic staff and visitors, and vaccination, were being implemented. As well as explaining the lower-than-expected respiratory infection rate,^18^ a ceiling effect from personal protective equipment cannot be ruled out. However, by the second winter (2022-2023) additional IPC use was falling and, apart from vaccination, was mostly absent by the third winter. The respective number of bedroom resident days contributing to the main outcome were 6787 (3.4%) in the first winter 1, 63 198 (31.4%) in the second winter, and 130 884 (65.0%) in the third winter. An explanatory study might have sought to establish if HEPA filters added value to any of the additional IPC activities. However, this was a pragmatic study, aiming to establish if HEPA filter use provided benefits compared with usual care, and we considered the additional burden of measuring and reporting other IPC activities was beyond care home staff capacity.

Third, there was low intervention adherence. However, staff reported that the HEPA filters were usually switched on and were correctly located. We considered and decided against conducting monitoring visits to check intervention compliance because the study was designed to be conducted remotely (during the pandemic), and because external compliance monitoring would have become part of the intervention, making the trial less pragmatic.

Fourth, there were also insufficient CADRs. We used the manufacturer-recommended CADR based on room volume. An explanatory study might have used more sophisticated modeling, including frequency and proximity of staff, visitor, and resident interactions and room ventilation levels to determine more precise CADR requirements.^29^ However, we do not believe that a greater number of HEPA filters, or the increased cost, air cooling, and noise associated with higher CADR units, would have been acceptable.

Fifth, the long-range airborne transmission considered potentially modifiable by HEPA filters might be minor compared to direct contact, direct deposition, and short-range airborne transmission in this population. Air quality monitors (such as measures of CO_2_ or fine particulate matter [PM_2.5_] concentration) would have helped us understand if the air remained contaminated despite the filters, or if the air was cleaned, but residents became infected via contact transmission. However, this would have become part of the intervention and required additional equipment costs and staff time, which we did not consider realistic in this underresourced setting. Finally, postrandomization recruitment bias appears to be an unlikely explanation given the low care home and resident dropout rates, and the comparability of care homes and residents in the 2 groups.

The primary, and many of the secondary outcomes, required staff to report the presence of resident symptoms daily, via an online database. We found rates of respiratory and gastrointestinal tract infections per bedroom resident per winter of approximately 1 and 0.1 (respiratory:gastrointestinal tract ratio of approximately 10), respectively. However, the respiratory:gastrointestinal tract ratio was similar to the approximately 8 per bedroom resident per winter previously observed in another care home study.^18^ Furthermore, removing care homes with outlier reporting rates did not affect the results, and respiratory episode durations were similar to those seen in previous studies.^18,19^

Among communal room residents, we observed evidence of reductions in respiratory infection episodes resulting in fever, delirium, and/or decline in physical functioning, and the number of days antibiotics were consumed. However, given the number of hypothesis tests performed, these findings are consistent with the number of false positives that would be expected due to type I error. We think that these results should be interpreted with caution, and it is our opinion that these findings likely reflect false positives due to multiplicity, rather than true differences between arms, particularly given the consistency of the results in the other outcomes.

Even with the lower-than-expected respiratory infection rate, AFRI-c was adequately powered to detect the 50% reduction in respiratory infections per resident per winter advised as clinically important by our public contributors (the 95% CI for the primary incident rate ratio analysis was 0.64-1.33). However, the 95% CI includes smaller, potentially clinically and cost-important reductions (eg, 25%).

## Conclusions

In this cluster randomized clinical trial, we found no evidence that HEPA filters reduced resident winter respiratory infection episodes, other infections, antibiotic use, or staff absenteeism. Care homes should continue to adhere to nationally recommended prevention measures to control respiratory and other infections.

## Notes

Supplement 1.
Trial Protocol

Supplement 2. **eTable 1. **Primary and secondary objectives and outcomes
eMethods
**eTable 2.** Description of residents’ intervention exposure **eTable 3.** Dates of key activities in participating care homes for AFRI-c **eTable 4.** Deviations from specified models **eFigure 1.** Communal room HEPA filter in-situ **eFigure 2.** Daily resident data collection for infection symptoms **eFigure 3.** Daily resident data collection confusion and falls/near falls **eFigure 4.** Duration of respiratory infection episodes by group **eTable 5.** Primary outcome pre-specified subgroup analyses: bedroom residents **eTable 6.** Secondary outcomes: bedroom residents **eTable 7.** Complier average causal effect (CACE) analyses: bedroom residents **eTable 8.** Baseline characteristics: communal room residents **eTable 9.** Secondary outcomes: communal room residents **eTable 10.** Complier average causal effect (CACE) analyses: communal room residents **eTable 11.** Secondary outcomes: staff absenteeism **eTable 12.** Secondary outcomes: care home microbiological outbreaks **eTable 13.** Description of turnover **eTable 14.** Primary and secondary outcomes presented per 1000 resident days: bedroom residents
eReferences

Supplement 3.
Data Sharing Statement
