# Supplementary material for: High-Efficiency Particulate Air Filters to Prevent Winter Respiratory Infections in Care Homes: The AFRI-c Cluster Randomized Clinical Trial
Source: JAMA Intern Med. 2026 Jul 27:e262199. Online ahead of print. doi: 10.1001/jamainternmed.2026.2199 (PMC13409133; doi:10.1001/jamainternmed.2026.2199)
Supplement: Supplement 1. — Trial Protocol [file jamainternmed-e262199-s001.pdf]

A cluster randomised controlled trial of Air Filtration to prevent symptomatic winter Respiratory Infections (including COVID-19) in care homes (AFRI-c)

Approvals and IDs	Reference
**Protocol version**	V12.0
**IRAS project ID**	298022
**Research Ethics Committee (REC)**	London - Harrow (Ref: 21/HRA/4318)
**Sponsor**	University of Bristol
**Funding**	NIHR PHR 129783
**ISRCTN**	ISRCTN63437172
**NIHR CRN CPMS**	50819

This protocol has regard for the HRA guidance

KEY TRIAL CONTACTS

Chief Investigator	Professor Alastair D Hay<br>GP and Professor of<br>Primary Care<br>Centre of Academic Primary Care<br>NIHR School for Primary Care Research<br>Bristol Medical School: Population Health Sciences<br>Canynge Hall, 39 Whatley Road<br>Bristol BS8 2PS<br><br>Tel: +44 117 928 7376<br>Email: Alastair.Hay@bristol.ac.uk
Co-investigators (applicants for funding)	Professor Chris Metcalfe, Professor of Medical Statistics<br>University of Bristol<br>Email: chris.metcalfe@bristol.ac.uk<br><br>Dr Ruth Kipping, Associate Professor in Public Health, Programme Director for MSc in Public Health, University of Bristol<br>Email: Ruth.Kipping@bristol.ac.uk<br><br>Dr Joanna Thorn, Research Fellow in Health Economic Evaluation, University of Bristol.<br>Email: Joanna.thorn@bristol.ac.uk<br><br>Ms Clare Clement, Senior Research Associate, University of Bristol<br>Email: c.clement@bristol.ac.uk<br><br>Dr Nicholas Turner, Research Fellow in Medical Statistics, University of Bristol<br>Email: Nicholas.Turner@bristol.ac.uk<br><br>Dr Tomas Welsh, Consultant geriatrician/Deputy Director of RICE, Royal United Hospital Bath, NHS Foundation Trust,<br>Email: tomas.welsh@bristol.ac.uk<br><br>Dr Emily Henderson, Consultant geriatrician and consultant Senior Lecturer, University of Bristol<br>Email: Emily.Henderson@bristol.ac.uk<br><br>Dr Michael Jenkins, Clinical Lead for Integrated care, NHS Bristol, North Somerset and South Gloucestershire Clinical Commissioning Group<br>Email: michael.jenkins1@nhs.net<br><br>Jane Sprackman, PPI<br><br>Karen Sargent, PPI<br><br>Dr Dominic Mellon. Lead Consultant Clinical Scientist in Public Health Infections at Publish Health England South West and COVID-19 incident lead for the South West Protection Team. Email: dominic.mellon@phe.gov.uk

	Dr Derren Ready. Consultant Clinical Scientist in Public Health Infections at Publish Health England South West.<br>Email: derren.ready@phe.gov.uk<br><br>Dr Gemma Morgan, Consultant in Public Health, South Gloucestershire Council<br>Email: Gemma.Morgan@southglos.gov.uk
Lead Statistician	Dr Nicholas Turner, Research Fellow in Medical Statistics Bristol Medical School: Population Health Sciences, University of Bristol<br>Email: Nicholas.Turner@bristol.ac.uk
Study website	https://afric.blogs.bristol.ac.uk/
UK-specifics	
Trial Manager (UK)	Dr Rachel Brierley, Research Fellow, Bristol Medical School: Population Health Sciences, University of Bristol<br>Email: R.Brierley@bristol.ac.uk<br>General trial email:<br>AFRIc-study@bristol.ac.uk

FUNDING AND SUPPORT IN KIND

Funder(s)	Financial and non-financial support given
NIHR Public Health Research (PHR) Programme<br>National Institute for Health and Care Research<br>Evaluation, Trials and Studies Coordinating Centre<br>University of Southampton<br>Alpha House, Enterprise Road<br>Southampton, SO16 7NS (UK)<br><br>Email: netsmonitoring@nihr.ac.uk	Grant funding
Bristol Trial Centre (BTC)<br>Bristol Trials Centre (BTC)<br>Bristol Medical School<br>University of Bristol<br>Office G.05<br>1-5 Whiteladies Road, Clifton<br>Bristol, BS8 1NU<br>Email: btc-mailbox@bristol.ac.uk	Methodological expertise
Adam Taylor<br>Head of Research Governance<br>Research and Enterprise Division<br>University of Bristol Second Floor<br>Augustines Courtyard<br>Orchard Lane<br>Bristol, BS1 5DS<br><br>Telephone: 01174556247<br>Email: research-governance@bristol.ac.uk	Sponsorship and research governance
Telephone: 07867901807<br>Email: lawrence.symes@philips.com	Commercial filter unit supplier
NHS Bristol, North Somerset and South Gloucestershire<br>Clinical Commissioning Group<br>South Plaza<br>Marlborough Street,<br>Bristol, BS1 3NX<br><br>Telephone: 0117 984 1673<br>Email: bnssg.research@nhs.net	Host

LIST OF ABBREVIATIONS

AE	Adverse Event
AR	Adverse Reaction
BRTC	Bristol Randomised Trials Collaboration
BTC	Bristol Trials Centre
C&C	Confirmation of Capacity and Capability
CCA	Cost–Consequences Analysis
CI	Chief Investigator
CONSORT	Consolidated Standards of Reporting Trials
COVID/COVID 19	Disease caused by SARS-CoV-2 Coronavirus
CRF or eCRF	Case Report Form or Electronic CRF
CRN	Clinical Research Network
CSO	Clinical Study Officer
CTA	Clinical Trial Authorisation
CTN	Clinical Trial Notification
CUA	Cost–Utility Analysis
DHSC	Department of Health and Social Care
DRP	Detailed Research Plan (submitted as part of NIHR EME funding application)
DSUR	Development Safety Update Report
EC	European Commission
ED	Emergency Department
EOI	Expression of Interest
EU	European Union
GDPR	General Data Protection Regulation
GE	Gastroenteritis
GLP	Green Light Process
GP	General Practitioner
HEPA	High-Efficiency Particulate Air
HES	Hospital Episode Statistics
HPU	Health Protection Unit
HRA	Health Research Authority
HMP	Human Metapneumovirus
ICH-GCP	International Conference on Harmonisation for Good Clinical Practice
ID	Intellectual Disability
I.D.	Identification
IDMC	Independent Data Monitoring Committee
ILI	Influenza-like illness
IPC	Infection Prevention and Control
ISF	Investigator Site File
ISRCTN	International Standard Randomised Controlled Trials Number
ITT	Intention-to-treat
MAR	Missing at Random
MHRA	Medicines and Healthcare Products Regulatory Agency
MIA	Manufacturer and Importation Authorisation
NHMRC	National Health and Medical Research Council
NHS	National Health Service
NHS R&D/R&I	National Health Service Research & Development/Research & Innovation
NICE	National Institute for Health and Care Excellence
NIHR	National Institute for Health and Care Research
NIHR CRN	National Institute for Health and Care Research Clinical Research Network
NIHR PHR	National Institute for Health and Care Research Public Health Research Programme

PCR	Polymerase Chain Reaction
PHE	Public Health England
PI	Principal Investigator
PICs	Patient Identification Centres
PIS	Participant Information Sheet
PPE	Personal Protective Equipment
PPI	Patient and Public Involvement
PQ	Participant Questionnaires
PROFANE	Prevention of Falls Network Europe
PROM	Patient Reported Outcome Measure
PSS	Personal Social Services
QALY	Quality Adjusted Life Years
QP	Qualified Person
RA	Research Assistant
RCT	Randomised Controlled Trial
R&D	Research and Development
RGO	Research Governance Office
REC	Research Ethics Committee
RSI	Reference Safety Information
RTI	Respiratory Tract Infection
RSV	Respiratory Syncytial Virus
SAE	Serious Adverse Event
SAR	Serious Adverse Reaction
SARS-CoV-2	Severe Acute Respiratory Syndrome Coronavirus 2
SAP	Statistical Analysis Plan
SD	Standard Deviation
SEAR	Screened, Eligible, Approached, Randomised (SEAR) framework
SLA	Service Level Agreement
SOP	Standard Operating Procedure
SSTI	Skin and Soft Tissue Infection
SUSAR	Suspected Unexpected Serious Adverse Reaction
TGA	Therapeutic Goods Administration
TMF	Trial Master File
TMG	Trial Management Group
TSC	Trial Steering Committee
UK	United Kingdom
UKCRC	UK Clinical Research Collaboration
UKHSA	UK Health Security Agency (formerly Public Health England)
UoB	University of Bristol
USM	Urgent Safety Measure
UTI	Urinary Tract Infection
WHO	World Health Organization

TRIAL SUMMARY

Trial title	Air Filtration to prevent symptomatic winter Respiratory Infections (including COVID-19) in care homes: the AFRI-c cluster randomised controlled trial with nested internal pilot, process and economic evaluations
Short title	Air Filtration to prevent symptomatic Respiratory Infections including COVID-19 in care homes (AFRI-c)
Acronym	AFRI-c
Chief Investigator	Professor Alastair D Hay, NIHR Senior Investigator, GP and Professor of Primary Care, Centre of Academic Primary Care, NIHR School for Primary Care Research, University of Bristol, UK
Sponsor	University of Bristol (UoB)
Funder	National Institute for Health and Care Research (NIHR) Public Health Research (PHR) Programme<br>(reference NIHR 129783)
Aim	The overall aim is to investigate the clinical and cost effectiveness of portable HEPA air filters in reducing symptomatic winter respiratory infections (including COVID-19) in residents of older people's care homes.
Trial design	A two-arm, open controlled trial with 1:1 cluster randomisation to intervention or control at care home level, stratified by nursing care provision and socioeconomic status, with nested: mixed-method process evaluation; internal pilot (with go/no-go criteria); and health economic evaluations.
Planned sample size	Approximately 91 - care homes (increase from original plan of 74 care homes)<br>Approximately 1002 – residents (increase from original plan of 740 residents)
Inclusion criteria	Eligibility will be assessed first by care home and then resident.<br>**Care homes** will be eligible if (all of): ≥20 residents residing in single bedrooms; care homes which predominantly focuses on care for older people (residential/nursing home); willing to maintain register of all residents; willing to invite eligible residents to receive filters and/or accept medical notes review until an average of 12 (maximum 16) agree to take part (referred to as the "consented"); care home owner permission to take part; willing to commit to installing air filters in in care home if allocated to intervention group; willing to commit to not installing air filters if allocated to control group.<br>**Residents** will be eligible if (all of): expected to reside in the care home for at least 1 month of the care home data collection period.<br>**Residents for the "consented"** will be eligible if (all of): in single occupancy bedroom; expected to reside in the care home for at least 1 month of the care home data collection period; able to give informed consent (or if lacks capacity, a consultee is willing to complete a consultee declaration form).
Exclusion criteria	**Care homes** will be ineligible if (any of): CQC website rates as 'inadequate' or 'requiring improvements' in any area; ≥10% private room use of portable HEPA filtration devices; or participating in competing care home study.<br>**Residents** will be excluded if (any of): terminal illness (death expected within seven days).<br>**Residents for the "consented"** will be excluded if (any of): participating in a competing study; terminal illness (death expected within seven days).
Number of care homes	Approximately 10 care homes for internal pilot study, a further 35 for the second winter and 46 for the third winter.
Duration	Funding start date: 1st June 2021<br>Anticipated duration: 40 months (total; subject to change)<br>Anticipated end date: 30th April 2024 (subject to change)
Primary objective	To investigate the effect of portable HEPA air filters on symptomatic winter respiratory infection episodes (including COVID-19) between residents in care homes with air filters in private residential, communal and staff-only rooms and residents in care homes without air filters in private residential, communal and staff-only rooms.
Secondary objectives	To determine the effect of air filters installed in private residential, communal and staff-only rooms during the winter months versus no air filters on residents':

	a) Respiratory infections (symptomatic days)<br>*b) Fever and/or delirium and/or acute deterioration in physical ability (episodes and symptomatic days)*<br>*c) Gastro-intestinal infection (episodes and symptomatic days)*<br>*d) Antibiotic use (courses prescribed/days consumed)*<br>*e) Number of falls/near falls*<br>*f) Possible SARS-CoV-2 and Influenza-like illness infection episodes*<br>*g) PCR confirmed SARS-CoV-2 infections in consented residents only*<br>*h) PCR (or other test) confirmed Influenza A&B infections in consented residents only*<br>*i) Other microbiologically confirmed infections including Streptococcal, Meningococcal, Respiratory Syncytial Virus, Norovirus and Human Metapneumovirus infections in consented residents only*<br>j) *GP diagnosed respiratory (including COVID), gastrointestinal, skin and urinary infections*<br>*k) Perception of care home environment*<br>*l) Hospitalisations (all cause, and potentially air filter preventable)*
	To determine the effect of air filters versus no air filters installed during winter months on staff members':<br>*a) Sickness days away from work*<br>*b) Sickness days away from work due to respiratory infections*<br>*c) Sickness days away from work due to any other infection*<br>*d) Change over time in staff confidence in, and use of, infection prevention and control strategies*<br>*e) Perception of care home environment*
	To determine the effect of air filters versus no air filters installed during winter months **on Care Homes':**<br>Number of days experiencing PCR or other microbiologically confirmed infection outbreaks
	To determine the cost-effectiveness of air filter use in reducing symptomatic respiratory infection episodes.
	To identify the views of local authority and CCG commissioners on intervention maintenance, sustainability and possible dissemination.
	In the intervention arm:<br>To explore staff, resident and residents' relatives/friends attitudes to and perceptions and experiences of air filters and determine factors influencing their use, including acceptability, satisfaction and potential benefits and harms.<br>To assess fidelity to the intervention.

TRIAL FLOW CHART

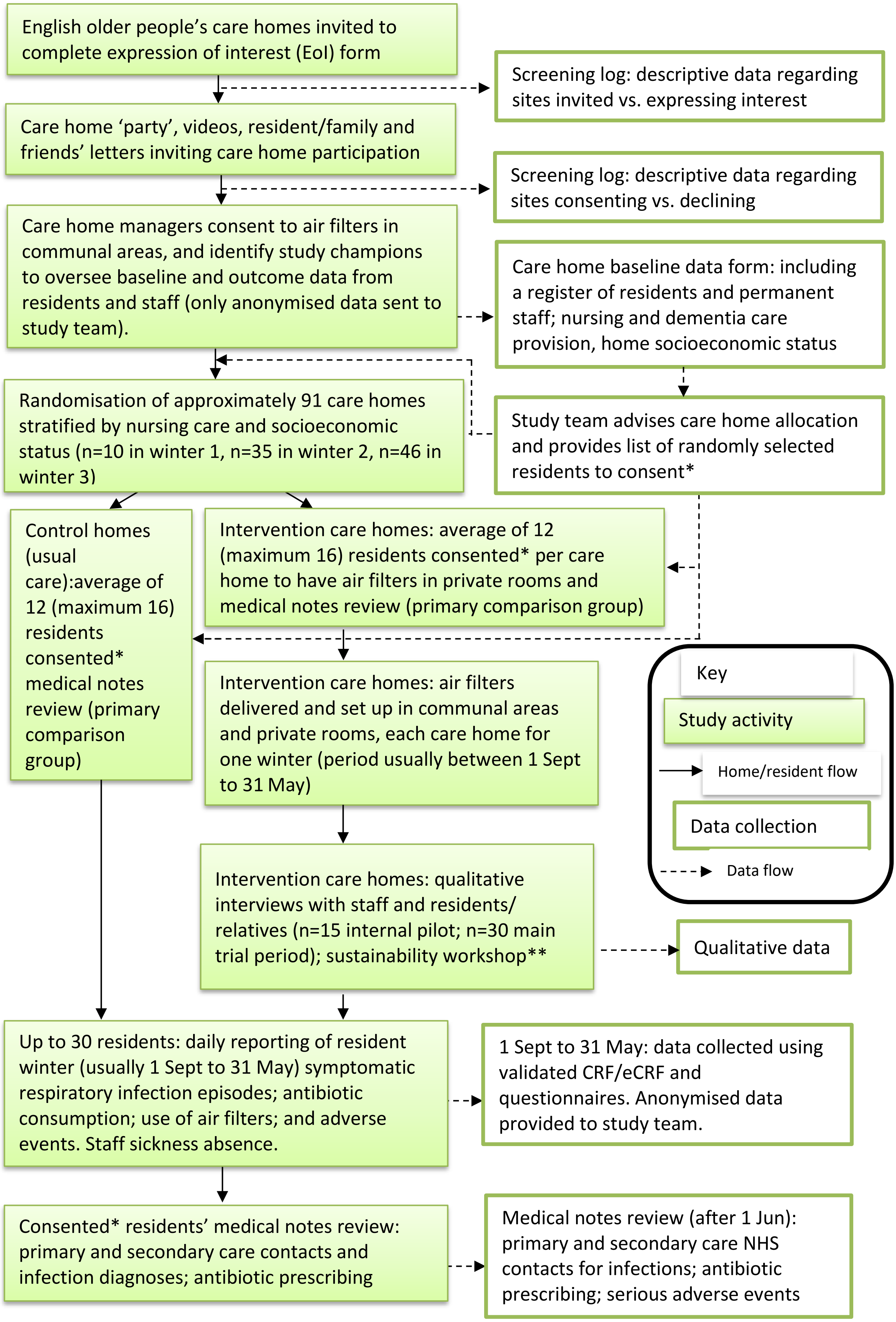

* consultee declaration for residents without capacity by study and CRN nurses trained in Mental Capacity Act
** additional stakeholders will be involved in the sustainability workshop

TABLE OF CONTENTS

KEY TRIAL CONTACTS 2

FUNDING AND SUPPORT IN KIND 4

LIST OF ABBREVIATIONS 5

TRIAL SUMMARY 7

TRIAL FLOW CHART 9

TRIAL PROTOCOL TITLE 13

1 BACKGROUND AND RATIONALE 14

2 AIMS AND OBJECTIVES 16

2.1 AIM 16
2.2 PRIMARY OBJECTIVE 16
2.3 SECONDARY OBJECTIVES 16
2.4 PRIMARY OUTCOME 17
2.5 SECONDARY OUTCOMES 17

3 TRIAL DESIGN 20

3.1 PROJECT TIMETABLE 20
3.2 INTERNAL PILOT 20
3.3 PLANNED RECRUITMENT 20
3.4 TAKING ACCOUNT OF THE SARS-COV-2 PANDEMIC 21

4 TRIAL SETTING 22

5 ELIGIBILITY CRITERIA 23

5.1 PARTICIPANT POPULATION 23
5.2 CARE HOMES 23
5.2.1 Inclusion criteria *23*
5.2.2 Exclusion criteria *23*
5.3 ALL RESIDENTS 23
5.3.1 Inclusion criteria *23*
5.3.2 Exclusion criteria *23*
5.4 THE “CONSENTED” RESIDENTS 24
5.4.1 Inclusion criteria *24*
5.4.2 Exclusion criteria *24*
5.4.3 Co-enrolment in other research studies *24*

6 TRIAL PROCEDURES 25

6.1 SCHEDULE OF TRIAL ASSESSMENTS AND OUTCOMES (OVERVIEW) 25
6.2 KEY TIME POINTS FLOW CHART 26
6.3 CARE HOME SELECTION 29
6.3.1 Identification and invitation to take part *29*
6.3.2 Expression of interest and screening *29*
6.3.3 Eligibility assessment *29*
6.3.4 Care home willingness to take part *29*
6.4 CARE HOME SET-UP AND RANDOMISATION 29
6.4.1 Nomination of care home study champions *29*
6.4.2 Green light process and study specific training *30*
6.4.3 Identification, eligibility assessment and the resident register (all residents) *30*
6.4.4 Care home baseline data collection *30*
6.4.5 Randomisation and allocation specific training *31*
6.4.6 Equipment provision *31*
6.5 INDIVIDUAL RESIDENT INVITATION 31
6.5.1 Identification of individual residents *31*
6.5.2 Invitation *32*
6.6 CONSENTED RESIDENTS BASELINE DATA COLLECTION 32
6.7 RESIDENT DATA COLLECTION DURING WINTER PERIOD 32
6.7.1 Data collected on up to 30 ELIGIBLE residents in intervention and control arms *33*
6.7.2 Data collection on ALL CONSENTED residents in intervention and control arms *33*
6.7.3 Data collected on CONSENTED residents in INTERVENTION arm only *33*

6.8 Care home follow up data collection 33
6.8.1 *Care home follow up data collection in ALL care homes* *33*
6.8.2 *Care home follow up data collection in INTERVENTION care homes* *33*
6.9 Air filter data collection 33
6.9.1 *Data collection in ALL care homes* *34*
6.9.2 *Data collection in INTERVENTION care homes* *34*
6.9.3 *Data collection in CONTROL care homes* *34*
6.10 Staff questionnaires 34
6.10.1 *Infection prevention control strategies questionnaire (IPC) (frequency: baseline and end of March)* *34*
6.10.2 *Care home satisfaction questionnaire (frequency: baseline and end of March only)* *34*
6.10.3 *Air filter satisfaction questionnaire (frequency: intervention care homes/end of March only)* *34*
6.11 Resident Questionnaires 35
6.11.1 *Infection Prevention Control Strategies Questionnaire (frequency: baseline and end of March)* *35*
6.11.2 *Care Home Satisfaction Questionnaire (frequency: baseline and end of March)* *35*
6.11.3 *Air filter Satisfaction Questionnaire (frequency: intervention care homes/end of March only)* *35*
6.12 UK Health Security Agency (formerly Public Health England) (collected retrospectively) 35
6.13 New residents 35
6.14 Qualitative outcomes 35
6.15 Change in participation status of care homes and participants 36
6.15.1 *Care home change in participation status* *36*
6.15.2 *Resident change in participation status* *36*
6.15.3 *Resident opt-out* *36*
6.16 Care home closedown 36
6.16.1 *Care home study paperwork, tablets and electricity monitors* *36*
6.16.2 *Removal of air filters* *37*
6.16.3 *Thanking care home staff for their involvement* *37*
6.16.4 *Thanking residents for their involvement* *37*
6.17 Methods/procedures to protect against other sources of bias 37
6.18 Contact procedure for care homes 37
6.19 Blinding 38
6.20 End of trial 38
6.21 Trial stopping rules 38

7 CONSENT 39

7.1 Overview 39
7.2 Consent of individual residents 39
7.2.1 *Residents with capacity to consent to take part* *40*
7.2.2 *Residents without capacity to consent to take part* *40*
7.2.3 *Follow-up* *41*
7.3 Methods of consent 42
7.3.1 *Face to face, consent consultation* *42*
7.3.2 *Remote consent consultation* *43*
7.4 Documenting consent 43
7.4.1 *eConsent /eDeclaration* *43*
7.4.2 *Verbal consent/ verbal declaration* *43*

8 MIXED METHODS PROCESS EVALUATION 44

8.1 Logic model 44
8.2 Quantitative data (questionnaires) 44
8.3 Qualitative Interviews 44
8.3.1 *Identifying and consenting participants* *45*
8.3.2 *Qualitative data analysis* *45*
8.4 Data management, protection and patient confidentiality in relation to the qualitative research data 46
8.5 Safeguarding residents during interviews 46
8.6 Sustainability Workshop 46

9 STUDY CLASSIFICATION 48

10 INTERVENTION 49

10.1 General information 49
10.2 Care home allocation 49
10.3 Distribution of air filters 49
10.4 Installation and use of air filter units 49
10.5 Air filter modes and settings 49

10.6 Maintenance of filters ... 49
10.7 Removal of air filters ... 50

11 CONTROL ... 51

12 SAFETY ... 52

12.1 Adverse event terms and definitions ... 52
12.2 Classification of severity ... 52
12.3 Classification of relatedness ... 52
12.4 Identification of AEs ... 53
12.5 Operational definitions for (S)AEs due to residents health status ... 53
12.6 Operational definition of SAEs related to research procedures ... 53
12.7 Recording and reporting SAEs related to the research procedure ... 53
12.8 Responsibilities related to SAE reporting ... 56
12.9 Safeguarding procedures ... 56

13 STATISTICS AND HEALTH ECONOMICS ANALYSIS ... 57

13.1 Main trial ... 57
13.1.1 Sample size calculation ... 57
13.1.2 Data and statistical analysis ... 57
13.1.3 Health economic evaluation ... 58

14 DATA MANAGEMENT ... 59

14.1 Source data and documentation ... 59
14.2 Storage and access to data ... 59
14.3 Archiving and destruction of trial materials ... 59
14.4 Access to the final study data set ... 60

15 TRIAL MANAGEMENT ... 61

15.1 Trial management group (TMG) ... 61
15.2 Trial Steering committee (TSC) ... 61
15.3 Independent Data monitoring committee (IDMC) ... 61
15.4 Patient and public involvement (PPI) ... 61
15.5 Collaborators ... 61
15.6 Sponsor and funding ... 62

16 MONITORING, AUDIT AND INSPECTION ... 63

16.1 Monitoring ... 63
16.2 Protocol compliance ... 63
16.3 Notification of serious breaches to GCP and/or the protocol ... 63

17 ETHICAL AND REGULATORY CONSIDERATIONS ... 64

17.1 Research governance ... 64
17.2 Governance and legislation ... 64
17.3 Research Ethics Committee (REC) review and reports ... 64
17.4 Peer review ... 64
17.5 Poor quality data ... 65
17.6 Financial and other competing interests ... 65
17.7 Risks and benefits ... 65
17.8 Indemnity ... 65

18 DISSEMINATION POLICY ... 66

19 SIGNATURE PAGE ... 67

20 REFERENCES ... 68

TRIAL PROTOCOL TITLE

Air Filtration to prevent symptomatic winter Respiratory Infections (including COVID-19) in care homes: the AFRI-c cluster randomised controlled trial with nested internal pilot, process and economic evaluations (AFRI-c).

1 BACKGROUND AND RATIONALE

Like most respiratory infections, SARS-CoV-2 is primarily spread by airborne droplets.(1) (2) To date SARS-CoV-2 infection has led to over one million deaths worldwide, with older care home residents disproportionately affected.(3) Between 2 March and 12 June 2020, there were 66,112 deaths in UK care home residents of which 19,394 (29%) were related to SARS-CoV-2.(4)

The high prevalence of frailty and multimorbidity in older people means they lack functional reserve to cope with any infection and are at risk of functional decline, high care needs, lower quality of life and increased mortality.(5) An episode of pneumonia in a resident with a low 'activity of daily living' score predicts recurrent pneumonia and death within 2 years.(6) A 2017 study reported a 16-day care home outbreak of respiratory syncytial virus and human metapneumovirus outbreak associated with 50% a hospital admission rate, and 17% mortality.(7)

The number of people living in UK care homes is projected to nearly double in the next 20 years, from 380,000(8) to 700,000.(9) Care homes are defined by law as establishments providing accommodation, together with nursing or personal care, for persons who are or have been ill,(10) and are either 'residential' or 'nursing' (we will use 'care home' to refer to both).

Recent research showed the odds of antibiotic prescription for care home residents is double that of non-care home residents of similar age.(11) Increased use of antibiotics increases the risk of *Clostridioides difficile* infection and antimicrobial resistance.(12) Care homes have been identified as 'reservoirs' of antimicrobial resistance,(13) which of itself is considered a major challenge to public health.(14) In 2014, antibiotic resistance was voted 'one of the greatest issues of our time' by the UK public, and has been on the UK government civil emergency risk register for over 10 years.

Registered providers of health and adult social care must adhere to, and demonstrate to the Care Quality Commission (CQC) compliance with the UK Department of Health and Social Care 'Code of Practice on the Prevention and Control of Infections'.(15) This recommends: good hand hygiene; use of personal protective equipment such as disposable gloves, aprons and face protection; good catheter management; cleaning of the environment/ medical devices; vaccination; food safety; and nursing residents with acute respiratory and gastrointestinal infections in isolation.(16)

Despite these measures, infections remain common in care home residents. Respiratory followed by urinary infections are the most commonly reported.(17) Microbial transmission takes place via three mechanisms: (i) airborne microbes generated during talking, coughing and sneezing; (ii) direct contact, via contaminated hands or blood/bodily fluid exchange, with staff often being unwitting vectors;(7) and (iii) indirect contact via fomites.(16) While there are no data regarding the relative contribution of different transmission modes, the two most common types of infection in care homes suggest airborne (respiratory) and direct contact (urinary) are important. Existing infection control methods focus on interrupting direct and indirect mechanisms, with evidence suggesting hand hygiene is effective.(18)

However, airborne transmission remains largely unmitigated. Microbes can remain airborne for up to one week.(19) Isolating residents is logistically difficult and cannot protect other residents prior to symptom onset (when many illnesses are most infectious). Vaccination can only reduce the transmission of microbes for which vaccines have been given. Despite many years of use within healthcare environments such as operating theatres, it remains unclear what effect air filtration has when used in other settings, including care homes, schools, day cares and workplaces.

Viral dimensions range between 20–30nm (nanometres), and bacteria 500–10,000nm (Figure 1).(20) SARS-CoV-2 is approximately 100nm in diameter.(21) However, viruses and bacteria usually become airborne via aerosols (diameter ≤5,000nm) or droplets (diameter ≥5,000nm).(22) (23)

Figure 1. Nanoscale with vertical red line showing typical HEPA filter particle size capture, Woodall©

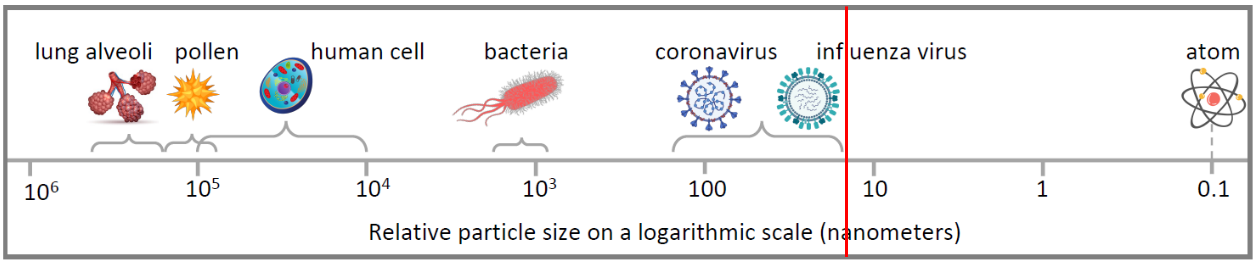

Aerosols and droplets contain some of the most pathogenic respiratory viruses (SARS-CoV-2, Influenza, Respiratory Syncytial Virus and Adenovirus,(22) bacteria,(20) and gastrointestinal viruses (such as Norovirus).(24) Once inhaled or swallowed, microbes can invade the respiratory (and gastrointestinal) mucosa, causing infection. Airborne particles may also land on surfaces and hands, increasing the chance of direct and indirect transmission. Reducing airborne microbes could therefore prevent respiratory as well as urinary, gastrointestinal and skin infections, transmitted via contamination of hands, medical equipment such as catheters, and fomites. Portable high efficiency particulate (HEPA) air filtration units, initially developed to remove vehicle emissions and pollen, trap particles ≥20nm including droplet and aerosolised bacteria and viruses.(20) One manufacturer has demonstrated its product capable of removing >99% of virus particles from a room-sized test chambers in 20 minutes.(25)

The UK government advisory SAGE Environmental and Modelling Group's 'Ventilation in controlling SARS-CoV-2 transmission' report,(26) advised 'air cleaning' devices can include one or more of: (i) fibrous (such as HEPA) filters; (ii) electrostatic precipitators; (iii) ionisers; (iv) ozone generators; (v) hydroxy-radical generators; and/or (vi) ultraviolet (UV) light. Only fibrous filters and UV light have been proven to remove viruses including SARS-CoV-2 with the principal advantage of fibrous (HEPA) filters being relative low cost and maintenance requirements.

We conducted a full systematic review (27) and found no studies investigating whether portable filters used in indoor settings prevent the incidence of respiratory (and other) infections. We did find two studies reporting removal and capture of airborne bacteria in indoor (office and emergency department) settings, but neither investigated the removal of viruses nor if infections were reduced. Another study (not included) compared outcomes for severely immunocompromised patients with and without built-in HEPA air filtration.(28) It reduced pneumonia incidence (7% vs. 17%, p=0·05). Finally, we are aware of a UV germicidal radiation trial in an acute hospital setting which demonstrated a substantial reduction in respiratory infections (average monthly rate 20·3 vs 8·6, p=0·001).(29)

In summary, HEPA air filters can capture relevant airborne particles; have been shown to reduce infections in severely immunocompromised patients; but there is an absence of evidence as to whether portable filters can prevent infection acquisition in care homes and other indoor settings. We agree with the SAGE-EMG report(26) that research is urgently needed to help health and social care providers know whether portable HEPA filters are clinically and cost-effective in reducing respiratory (and other) infections.

2 AIMS AND OBJECTIVES

2.1 Aim

The overall aim is to investigate the clinical and cost effectiveness of portable HEPA air filters in reducing symptomatic winter respiratory infections (including COVID-19) in residents of older people's care homes.

2.2 Primary objective

To investigate the effect of portable HEPA air filters in private residential, communal and staff only rooms on residents' symptomatic winter respiratory infection episodes (including COVID-19) in care homes with air filters compared with care homes without air filters.

2.3 Secondary objectives

1. To determine the effect of air filters installed in private residential, communal and staff only rooms during the winter months versus no air filters **on residents'**:
    a) Respiratory infections (episodes[B] and symptomatic days[A, B])
    b) Fever and/or delirium and/or acute deterioration in physical ability (episodes[A, B] and symptomatic days[A, B])
    c) Gastro-intestinal infection (episodes[A, B] & symptomatic days[A, B])
    d) Antibiotic use (courses prescribed[A]/days consumed[A, B])
    e) Number of falls/near falls[A, B]
    f) Possible SARS-CoV-2 and Influenza-like illness infection episodes[A, B]
    g) PCR confirmed SARS-CoV-2 infections[A,]
    h) PCR (or other test) confirmed Influenza A&B infections[A,]
    i) Other microbiologically confirmed infections including Streptococcal, Meningococcal, Respiratory Syncytial Virus, Norovirus and Human Metapneumovirus infections[A,]
    j) GP diagnosed respiratory (including COVID), gastrointestinal, skin and urinary infections[A]
    k) Perception of care home environment[A]
    l) Hospitalisations (all cause, and potentially air filter preventable)[A]

    A = an average of 12 (maximum 16) randomly selected residents per care home will be consented to provide permission to access medical records. In intervention care homes, these "consented" residents will also be invited to receive air filters in their private bedroom.
    B = up to (approx) 30 randomly selected residents (all residents where the care home has ≤30 residents)

2. To determine the effect of air filters versus no air filters installed during winter months **on staff members'**:
    a) Sickness days away from work
    b) Sickness days away from work due to respiratory infections
    c) Sickness days away from work due to any other infection
    d) Change over time in staff confidence in, and use of, infection prevention and control strategies. Perception of care home environment

3. To determine the effect of air filters versus no air filters installed during winter months **on Care Homes':**
    a) Number of days experiencing PCR or other microbiologically confirmed infection outbreaks

4. To determine the cost-effectiveness of air filter use in reducing symptomatic respiratory infection episodes (to include hospital admissions, deaths and cause of death).

5. To identify the views of care homes, local authority and CCG commissioners on intervention maintenance, sustainability and possible dissemination.

6. **In the intervention arm:**
   a) To explore staff, resident and residents' relatives' attitudes to and perceptions and experiences of air filters and determine factors influencing their use, including acceptability, satisfaction and potential benefits and harms.
   b) To assess the fidelity of the intervention.

2.4 Primary outcome

The primary outcome is the number of symptomatic winter respiratory infection episodes measured using case report forms (CRFs) completed by staff and/or the resident. 'Winter' is usually defined as the period starting on 1 September and ending on 31 May.

The definition of a symptomatic respiratory infection is "one or more new (or worsening of pre-existing) objective respiratory symptoms from the following: runny/blocked nose; sneezing; runny ear; red/sticky eye/s (part of the respiratory tract due to the lacrimal duct); hoarse voice; cough; wheeze; noisy breathing; or sputum (phlegm)". Shortness of breath will also be collected (as it contributes to UKHSA's definition of possible Influenza-like illness) but when present in isolation will not be regarded as a symptomatic respiratory infection episode. The subjective respiratory symptoms (sore/tickly throat, earache and change in taste/smell) will similarly be collected where the resident is able to communicate their presence for sensitivity analyses.

The start and end of a discrete symptomatic respiratory infection episode will be defined as per previous studies:(35) (36) (37) the start will be the onset of two new (or worsening of pre-existing) respiratory symptoms for ≥1 day or one respiratory symptom for ≥2 days; the end will be the last symptomatic day preceding two asymptomatic days.

2.5 Secondary outcomes

Secondary outcomes are summarised in Table 1 below. Outcomes will be measured at the time intervals indicated in Table 1 below. See Table 3 in section 6 for full details of trial assessments and time-points.

Table 1. Summary of primary and secondary outcomes and measures (tools).

Outcome	Tool / method
**Primary outcome**	
Symptomatic winter respiratory infection episodes	Measured by staff using daily case report forms (CRF)/electronic case report forms (eCRF).
**Secondary outcomes**	
**Secondary objective 1 (residents)**	
a. Number of days with respiratory infection symptoms	Daily CRF/eCRF
b. Presence of fever and/or delirium and/or acute deterioration in physical ability	Daily CRF/eCRF
c. Number of gastro-intestinal infection episodes and number of symptomatic days of gastro-intestinal infection symptoms	Daily CRF/eCRF
d. Number of days antibiotics are:<br>- Consumed<br>- Prescribed (number and name)	Daily CRF/eCRF<br>Medical notes review (for consented residents only*)
e. Number of falls/near falls	CRF/eCRF

f. Number of possible** SARS-CoV-2 infection episodes and possible** Influenza-like illness episodes	Daily CRF/eCRF
g. Number of PCR confirmed SARS-CoV-2 infections	UKHSA Capacity Tracker (for consented residents only*)
h. Number of PCR (or other test) confirmed Influenza A&B infections	UKHSA Capacity Tracker (for consented residents only*)
i. Number of other microbiologically confirmed infections as investigated by PHE during care home outbreaks, including Streptococcal, Meningococcal, Respiratory Syncytial Virus, Norovirus and Human Metapneumovirus infections	UKHSA Capacity Tracker (for consented residents only*)
j. Number of diagnosed respiratory (including COVID), gastrointestinal, skin and urinary infections	Medical notes review (for consented residents only*)
k. Perception of care home environment	Resident questionnaires (for consented residents only*)
l. Hospitalisations (all cause, and potentially air filter preventable)§	Medical notes review (for consented residents only*)
**Secondary objective 2 (staff)**	
a. Number of sickness days away from work	CH manager CRF/eCRF daily/weekly
b. Working days lost to sickness due to respiratory infections	CH manager CRF/eCRF daily/weekly
c. Working days lost to sickness due to any other infection	CH manager CRF/eCRF daily/weekly
d. Change over time in staff confidence in, and use of, infection prevention and control strategies.	Staff questionnaires
e. Perception of care home environment	Staff questionnaire (optional)
**Secondary objective 3 (Care Homes)**	
Number of days experiencing PCR or other microbiologically confirmed infection outbreaks	HPZone data supplied by UKHSA
**Secondary objective 4 (consented residents)**	
Overall healthcare resource use (costs)	Medical notes review/care homes notes review/eCRF (for consented residents only*)
**Secondary objective 5**	
Identify the views of care homes, local authority and CCG commissioners on intervention maintenance, sustainability and possible dissemination.	Sustainability workshop and stakeholder opinion market research
**Secondary objective 6 (intervention only)**	
a. To explore staff, resident and residents' relatives/ friends' attitudes to and perceptions and experiences of air filters and determine factors influencing their use including acceptability, satisfaction and potential benefits and harms.	Qualitative interviews, staff and resident questionnaires (consented residents only*)
b. Assess fidelity to intervention	Weekly CRF/eCRF and qualitative interviews

* average of 12 (maximum 16) residents per care home will be consented (from a list selected at random) to receive a private bedroom air filter and/or medical notes review

** Using UKHSA ‘possible’ case definitions: (i) COVID - new continuous cough OR temperature ≥37.8°C OR loss of, or change in, normal sense of smell or taste; and (ii) ILI - temperature ≥37.8°C AND one of the following: acute onset of at least one of the following respiratory symptoms (cough (with or without sputum), hoarseness, nasal discharge or congestion, shortness of breath, sore throat, wheezing, sneezing) OR an acute deterioration in physical or mental ability without other known cause.

§ Reasons for hospital admissions will be reviewed to identify those potentially considered preventable by air filters, namely: respiratory tract infections (+/- sepsis); urinary tract infections (+/- sepsis); skin and soft tissue infections (+/- sepsis); sepsis (no focus identified); trauma (+/- fracture); and major adverse cardiovascular events (MACE). MACE will be further categorised into: *arterial* (myocardial infarct, angina, new/exacerbation heart failure, transient ischaemic attack, haemorrhagic or thrombotic cerebrovascular accident) and *venous* (pulmonary embolism or deep vein thrombosis of the upper or lower limb).

3 TRIAL DESIGN

A two-arm, cluster randomisation trial of portable HEPA air filters versus no air filters for reducing symptomatic winter respiratory infections (including COVID-19) in care home residents with mixed method process and economic evaluation.

3.1 Project timetable

The funding start date for this trial is 01 July 2021 and the study duration is expected to be 42 months, to 31st December 2024 (subject to change).

3.2 Internal pilot

Following set-up, we will carry out a nested internal pilot study for up to six months (winter 1 will end 31 May, assuming COVID-19 is still prevalent). We aim to confirm care home and resident recruitment, follow up and event rates, the intervention is being used as intended and assess unintended consequences. Detailed information will be gathered during the internal pilot from both the qualitative and quantitative elements of the study to inform optimisation and refinement of study processes; and stop/go progression criteria (Table 2 below).

Table 2. Progression criteria, stop/go with a traffic light progress

Stop/go progression criteria			
Criterion (planned number by end of winter 1 – 31 May)	Traffic Light Progression		
	Stop (if any of):	Amend (if any of):	Go (if all of):
Care home consented to use of air filters in communal rooms.	≤5 care homes consented	6-7 care homes consented	≥8 care homes consented
Mean of intervention care home residents consented to have air filter in private bedroom .	<5 residents consented per care home	5-7 residents consented per care home	>7 residents consented per care home
Communal, staff-only, and private bedroom air filters sited as intended and not switched off for a mean of the checks.	<40% of checks	40-50% of checks	>50% of checks
Communal, staff-only, and private bedroom air filters cleaned, and air filter replacement for a mean of the checks.	<30% of checks	30-50% of checks	>50% of checks
Primary outcome data collection (days)	<4652 resident days complete data for main comparison primary outcome	≥4652 and <5351 resident days complete data for main comparison primary outcome	≥5351 resident days complete data for main comparison primary
Primary outcome data collection (proportion)	<70% follow up days complete for primary outcome among consented residents	≥70% and <80% follow up days complete for primary outcome among consented residents	≥80% follow up days complete for primary outcome among consented residents

3.3 Planned recruitment

A total of 91 care homes will be recruited for AFRI-c, with a mean cluster size of 12 consented at the start of the winter period. We anticipate recruiting at least 1002 consented participants throughout the course of the study to allow for drop out and the model of continuous recruitment. Further details about the trial setting are provided in the next section (Section 4). Recruitment is seasonal, therefore each care home will only be involved for 1 winter season. The duration of recruitment will be over 3 winters. In our recruitment progression estimates, we assumed that 10 care homes noted in Section 3.2 would be set-up and recruiting by month 6 of the internal pilot phase.

3.4 Taking account of the SARS-CoV-2 pandemic

The trial was designed anticipating the SARS-CoV-2 pandemic would remain active for at least the first study year. Since care home managers advised that access for research staff to care homes may be restricted for the foreseeable future, it was concluded that a ‘remote’ data collection policy was required, with research staff supporting study activities with telephone and video communications. The main risk of this approach is that staff will be too busy with care home responsibilities to assist with study activities.

To minimise this, we have decided: (i) to select care homes with that are the most engaged in the study with varying levels of research experience, e.g. via the NIHR ENRICH network; (ii) to request care homes provide the study team with anonymised data such that consent is necessary only for air filters to be installed, and for medical notes review; (iii) consent will be overseen by CRN/ study researcher experienced and trained in the enrolment and retention of older people with and without capacity to consent; (iv) all data will be collected by care home staff (i.e. not relying on residents to grapple with website forms) using simple-to-use forms collecting the minimum, necessary data; (v) to appoint ‘care manager study champions’ to ensure smooth running of study processes and data completion, and be points of contact; and (vi) to provide the care homes with financial reimbursement.

4 TRIAL SETTING

This trial will be delivered through care homes providing personal, with/without nursing, with/without dementia care to people predominantly over the age of 65 years in the UK. We will approach NIHR ENRICH Research Ready Care Home Network. Additional care homes will be identified via other routes, if required. Where possible, care homes will be selected to facilitate: (i) maximum NIHR CRN nurse support; and (ii) geographically clustered to facilitate the study researcher visits.

5 ELIGIBILITY CRITERIA

5.1 Participant population

Adults residing in a care home in the England.

5.2 Care homes

5.2.1 Inclusion criteria

- Capacity for ≥20 residents residing in single bedrooms*
- Care homes which predominantly focuses on care for older people (residential home/nursing home)
- Willing to maintain a register of all residents, to be updated weekly until the end of April, after which new residents will not be invited
- Willing to invite residents to receive air filters and/or accept medical notes review until average of 12 (maximum 16) agree (the "consented") per care home
- Willing to provide anonymised resident infection data, administer brief resident and staff questionnaires, and respond to data queries
- Care home owner permission to take part in study
- Willing to commit to installing air filters in care home if allocated to the intervention group
- Willing to commit to not installing air filters if allocated to control group

5.2.2 Exclusion criteria

- CQC website rates as 'inadequate' or 'requiring improvements'
- ≥10% private bedroom use of portable HEPA filtration devices
- Participating in a competing** care home level study

<u>*single bedrooms:</u> residents residing in a room of single occupancy will be referred to as residents in a private and/or single bedroom. Residents may share bathroom facilities.

<u>**Competing studies:</u> Competing studies are those whose aim (or a plausible consequence) is to prevent infections or reduce infection severity. Co-enrolment in the AFRI-c study and another competing study should be avoided due to potential impact on the study objectives and resident burden and safety.

5.3 All residents

5.3.1 Inclusion criteria

- Expected to reside in the care home for at least 1 month of the care home data collection period

5.3.2 Exclusion criteria

- Terminal illness (death expected within seven days)

We recognise that it will take more time and effort to proactively enrol residents who are more vulnerable. It is imperative however that active steps should be taken TO ENROLL the following groups of residents wherever possible. Those:

- With extreme age e.g. >90 years of age and/ or advanced frailty
- Who are bed-bound
- Who are usually semiconscious or unconscious, or whose conscious levels vary
- Who have a diagnosis of dementia, or have cognitive impairment

5.4 The "consented" residents

The following eligibility criteria are for the "consented" residents invited to receive an air filter and/or permit medical notes review **only**:

5.4.1 Inclusion criteria

- In single occupancy bedroom
- Expected to reside in the care home for at least 1 month of the care home data collection period
- Able to give informed consent (or if lacks capacity, a consultee is willing to complete a consultee declaration form)

5.4.2 Exclusion criteria

- Participating in any interventional* study
- Terminal illness (death expected within seven days)

5.4.3 Co-enrolment in other research studies

*Interventional study: Interventional studies are those which involve an 'intervention'. In medical terms this could be a drug treatment, surgical procedure, diagnostic test or psychological therapy. Examples of public health interventions could include action to help someone to be physically active or to eat a more healthy diet. Examples of social care interventions could include safeguarding or support for carers. If a site becomes aware that a participant has enrolled in an interventional study whilst taking part in AFRI-c, they should inform the central research team (UoB). The research team will evaluate whether it is appropriate for the resident to continue participating in AFRI-c.

6 TRIAL PROCEDURES

Approximately 91 care homes will be recruited and randomised (enrolled) over a 22-month period. This section outlines the key trial procedures from identification of potential care homes (section 6.3 and 6.4) and residents (section 6.5) through to end of the trial. An overview of the trial is shown in a flow diagram on page 9. An overview of key time points are depicted in the flow diagram below on page 25.

6.1 Schedule of trial assessments and outcomes (overview)

Table 3, below, details the key assessments/outcome measures along with care home and participant-related procedures scheduled at various trial timepoints. The care home data collection period will be the winter months (usually defined as 1 September to 31 May assuming COVID-19 is still prevalent) during which the air filters will be switched on and anonymised infection data collected daily from all eligible residents that do not opt-out of study involvement.

To summarise, the following research activity will take place:

1. Care homes will be identified and screened
2. Care homes will sign up to take part in the trial and be randomised
3. Eligible residents will contribute baseline and follow-up data on a daily basis over the winter period (capped at approx 30 randomly selected residents for winters 2 & 3)
4. Care home staff will be invited to complete baseline and follow up questionnaires on an optional basis
5. Some care home staff, residents/consultees and residents' relatives will be invited to take part in a qualitative interview

A subgroup of residents (average of 12, maximum of 16 per care home) will be selected at random to be consented to provide permission to access medical records. In intervention care homes, these "consented" residents will also be invited to receive air filters in their private bedroom. The consent process for these residents will be detailed in section 7.

6.2 KEY TIME POINTS FLOW CHART

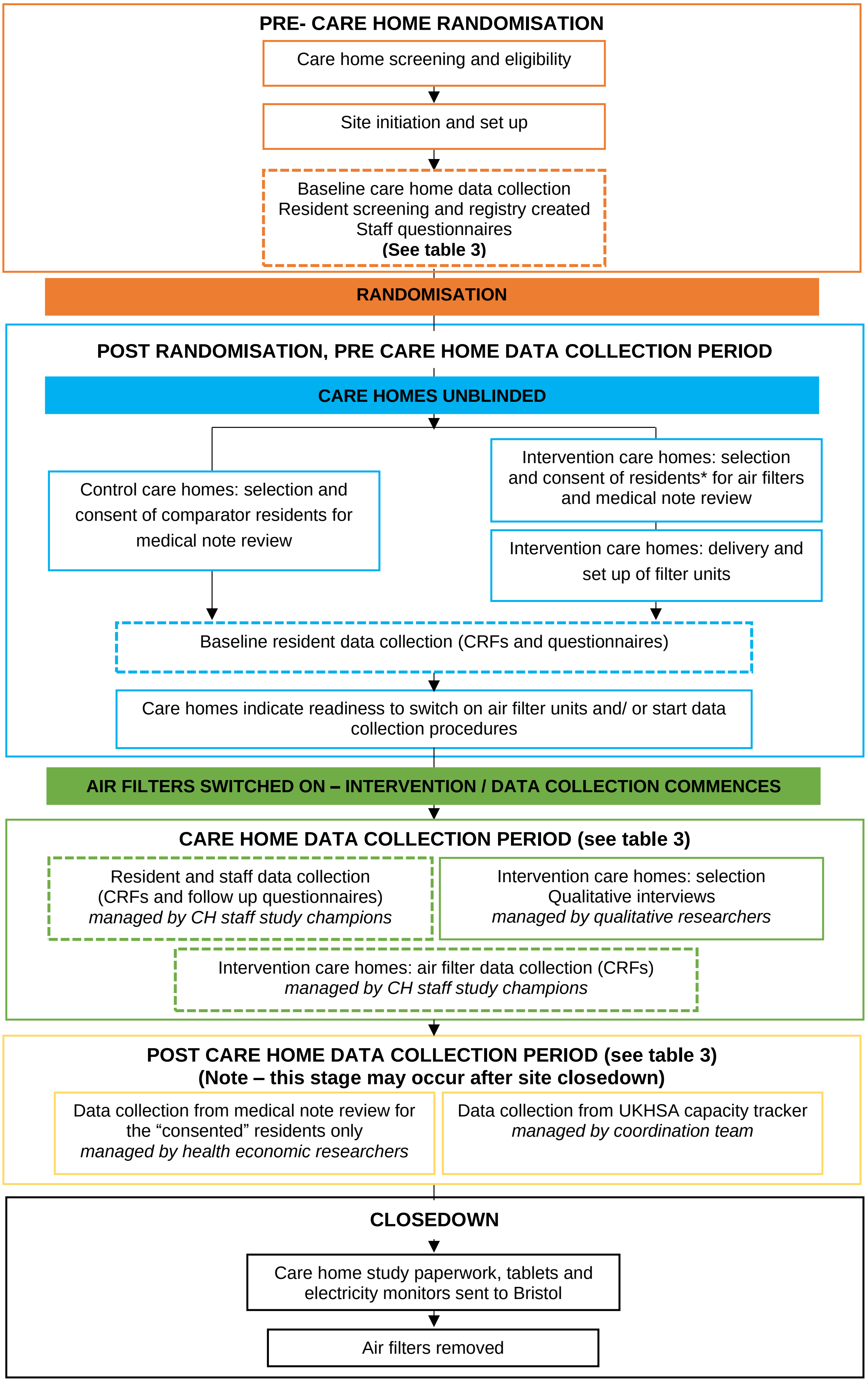

* Replacement residents may be consented throughout the care home data collection period. Residents are eligible if expected to reside in care home ≥1 month during the care home data collection period and if willing to accept same units with new air filters.

Table 3. Trial assessments with care home and resident-related procedures.

Data item:		Data collection time points				
	Baseline (CRF/eCRF) (August)	Care home data collection period<br>Follow up: [usually] 1 September to 31 May [a, b]			Post care home data collection period<br>~May and June[c]	
		Daily / weekly, for up to approx 30 residents, throughout data collection period (CRF/eCRF)	Weekly, for whole care home, throughout data collection period (CRF/eCRF)	Satisfaction questionnaires & care home follow up data collection (March)	Medical note review ****CONSENTED RESIDENTS ONLY****	UK Health Security Agency capacity tracker
**Residents data collection**						
Demography	●					
Resident registry	●		●			
Vaccination status (influenza, coronavirus)	●					
Frailty score	●					
Infection prevention and control strategies & satisfaction of care home environment questionnaires ***consented residents only***	●			●		
Respiratory infection symptoms (including COVID)[e]	●	●				
Fever[f,g], delirium[f] and/or acute deterioration in physical ability	●	●				
Gastrointestinal infection symptoms	●	●				
Medically diagnosed infections (including respiratory, gastro-intestinal, SARS-CoV-2, urinary, skin and soft tissue infections)					●	
PCR (or other) test confirmed infections (including SARS-CoV-2, Influenza A&B, respiratory (including COVID), other)						●
Antibiotic consumption		●				
Additional care ***consented residents only***		●				
Antibiotics prescribed (name and number)					●	
Frequency of adverse events (falls, injuries, hospital admissions and deaths)[d]		●			●	

****Intervention care homes only****						
Resident Air Filter satisfaction questionnaire (satisfaction with air filters in private bedrooms) ***consented residents only***				●		
Resident and residents' relatives/friends' satisfaction of air filters interviews (for residents who consent to interviews only)		*Assessed by interviews carried out throughout care home data collection period*				
Hospitalisations (all cause, and potentially air filter preventable)					●	
**Care home staff data collection**						
Staff absenteeism			●			
Staff demography (roles, staffing levels, funding etc)	●			●		
Infection prevention and control & satisfaction with care home environment questionnaires (optional)	●			●		
****Intervention care homes only****						
Staff Air Filter satisfaction questionnaire (optional)				●		
Staff satisfaction with air filters interviews (only for staff who give consent)		*Assessed by interviews carried out throughout care home data collection period*				
**Air filter data collection**						
Use of additional air filters in communal, staff and private bedrooms (acquired outside of the study)	●	●		●		
****Intervention care homes only****						
Air filters in private and communal areas are switched on, maintained and in the correct location.	●	●				
**Care home level data collection**						
Number of days experiencing PCR or other microbiologically confirmed infection outbreaks						●

[a] *Expected time periods for winters 2 and 3. Internal pilot timing will differ.*

[b] *Period within which intervention care homes are requested to turn on air filters, and both intervention and control group care homes will collect daily symptom data.*

[c] *Allowing time for medical records to be updated with letters from other providers. For winters 1 & 2 this period of data collection may occur later.*

[d] *See serious adverse event section 12*

[e] *To be determined at data analysis where the start/ end definition will be as per previous studies:(30) (31) (32) the onset of two new (or worsening of pre-existing) respiratory symptoms for ≥1 day or one respiratory symptom for ≥2 days; the end the last symptomatic day preceding two asymptomatic days*

[f] *To inform primary outcome sensitivity analyses and take account that infections in older people may present non-specifically(33)*

[g] *Temperature ≥37.8°C*

6.3 Care home selection

6.3.1 Identification and invitation to take part

Care homes belonging to organisations such as the NIHR ENRICH Research Ready Care Home Network and other networks known to the co-applicants will be approached via the CRN / directly by the study team using a study Research Information Sheet for Care Homes (RISCH) or provided access to other study promotional materials. Care homes may also be approached through other networks (e.g. NHS organisations doing research in non-NHS settings). Care homes may also contact the study team as a result of hearing about the study through other methods (e.g. finding the website online, word of mouth in care home networks).

6.3.2 Expression of interest and screening

Interested care homes will be asked to complete an expression of interest form (EoI), containing information required for the study team to carry out an eligibility assessment.

The CRN / study team will keep a screening log to record which care homes have been invited and which respond.

6.3.3 Eligibility assessment

Eligibility will be determined using the EoI form. Eligibility will be checked against the criterion in section 5.2 Any care homes found to be ineligible will be informed of why they are not eligible to take part in the study. For those that seem eligible, the care home manager will be invited to an initial meeting with the study team to discuss the study, answer any questions and check if the care home manager is willing to take part. If they are then next steps will be discussed.

6.3.4 Care home willingness to take part

Care home managers will be asked to share information about the study with staff, residents and resident's friends/family. They will be encouraged to put up posters (overview infographic) in visible areas around the care home. Information describing the aims of the study (taken from the RIS) will be shared to enable care home management teams to communicate information via email / newsletters with residents / families / friends. Care homes will also be asked to share the study overview video with residents and families/ friends. Members of the study research team may attend meetings if required. Refreshments can be provided as appropriate.

If residents or their friends/family express a concern about the study and do not wish to contribute, this will be recorded on the resident register as appropriate and no further study or data requests will be made of these individuals (see section 6.16.3). Where the majority view is to participate, the care home manager will confirm their willingness to support the trial by returning completed study set-up documents.

Care homes will be provided with some financial support for study activities (as detailed in the site agreement). There may also be some service support costs available through their local Clinical Research Network. The trial management team will provide relevant training to care home staff and will support care home teams throughout the research. A researcher will support staff with assessing capacity and consenting residents / consultees as required.

6.4 Care home set-up and randomisation

6.4.1 Nomination of care home study champions

Study champions will be integral to the success of this study. At least two study champions per care home will be appointed to oversee study processes and support the care home staff who will conduct most of the day-to-day study activities. The PI may be a study champion, or they may delegate responsibility for the smooth running of the study to named study champion(s).

Study champions will:

- Help invite and consent an average of 12 (maximum 16) residents per care home (see section 7 for consent details)
- Complete CRFs to record daily resident respiratory and gastro-intestinal infection symptoms, delirium, fever, antibiotic consumption, resource use and frequency of adverse events taking ~2 minutes (all residents)
- Support consented residents to complete questionnaires relating to infection prevention and control and their satisfaction of the care home environment, at baseline and at the end of March (May for winter 1).
- Help to identify and consent staff, residents and residents relatives/ friends to take part in qualitative interviews (actual consent will be taken by study team, see section 7.2)

In addition, care homes allocated to the intervention arm will:

- Receive, unpack and label air filters (see section 10.4)
- Install air filters (with electrician supervision if necessary)
- Ensure completion of case report forms (CRFs) about the air filters to record:
    - Daily checks (switched on/off, location)
    - Weekly checks (air filter unit is cleaned, filter replacement requirement, electricity monitor reading)
- Support consented residents to complete questions relating to their satisfaction of air filters, at baseline and at the end of March
- Help to identify and consent staff, residents and residents' relatives/close friends to take part in qualitative interviews (actual consent will be taken by study team, see section 7.2)

6.4.2 Green light process and study specific training

The Green Light Process (GLP) will consist of online study specific training for the care home manager and study champions, in key study elements e.g. supporting the invitation and consent/declarations (residents/consultees) processes, data collection, safety reporting and use of the study specific database. Care homes will have office hours access to team expertise, including a Mental Capacity Act trained central researcher experienced in the care and research of older people. Completion of training modules will be prompted and monitored using an automated online system or similar equivalent.

Once the care home has received the general site training and completed the set-up documents (including providing aggregate care home baseline data, section 6.4.4), the greenlight will be given to commence research activity.

6.4.3 Identification, eligibility assessment and the resident register (all residents)

At each care home, trained staff (managers and study champions) will be asked to provide a register for all residents at the care home (section 6.4.3). The study champions will confirm which residents meet the eligibility criteria in section 5, this will form the screening log. To ensure residents are easily identifiable to care home staff, resident names will be recorded on the database which will only be visible to the care home staff and staff consenting residents (delivery teams and the central research nurse). Their name will be recorded next to a corresponding study ID, both of which are visible to the care home / consenting staff. When the data is passed to the rest of the study team, only anonymised data will be visible (only the study ID will be visible). The register will be maintained throughout the care home data collection period (see 6.8.1).

6.4.4 Care home baseline data collection

Baseline data collection will include data captured on the EoI form and the information detailed below. The care home manager will complete this using an online care home baseline data collection form. We understand some data will vary with time, and that this is essentially a baseline cross sectional survey.

Staff roles and numbers (FTE, permanent and agency); CQC rating; number and type of rooms in care home; care home ventilation policy (windows open/closed); IPC policy; care home manager and additional study champion contact details; funding (private/public); use of additional air filters in communal, staff and private bedrooms (acquired outside of the study).

For all eligible residents, regardless of consent status the following anonymised aggregate baseline data will be collected by trained staff: age; sex; ethnicity; receiving nursing care; vaccination status.

On an individual basis residents' frailty will also be assessed using the clinical frailty scale (a judgement-based frailty tool that evaluates specific domains including comorbidity, function, and cognition to generate a frailty score ranging from 1 (very fit) to 9 (terminally ill)).

Staff will be asked to complete the (optional) infection prevention and control & care home satisfaction questionnaires (see section 6.10).

We will also ask care homes to provide a copy of their local safeguarding policies for the study records.

6.4.5 Randomisation and allocation specific training

Following green light activation, and baseline data being received, the care homes will be randomised to receive either the intervention (usual care plus HEPA filters) or control (usual care) (see section 10 and 11). Randomisation will be stratified by whether they provide nursing care yes/no; and socioeconomic tertile (high/medium/low)) using a randomisation list pre-generated by a CTU statistician with no other involvement in recruitment activities.

Following randomisation, care homes will be informed of their allocation.

Intervention care homes will complete additional study training, specific to the use of devices in their care homes – e.g. installation, monitoring and maintaining air filters (according to manufacturer instructions), recording of outcomes and the arrangements for delivery of the air filters to the relevant care homes. For care homes allocated to the intervention, training will also involve discussions of where to locate communal room air filters. Staff will then begin inviting an average of 12 (maximum of 16) residents per care home to participate to receive an air filter in their private bedrooms and/or provide access to their medical records (see section 6.5) and share identifiers with the UKHSA.

In the control care homes staff will begin inviting an average of 12 (maximum of 16) residents per care home to participate provide access to their medical records (see section 6.5) and share identifiers with the UKHSA.

6.4.6 Equipment provision

All care homes will be provided with at least one tablet to aid live data collection. In addition, care homes allocated to the intervention will be provided with the following equipment:

- Up to 21 air filters whereby a maximum of 16 will be placed in private bedrooms and 5 in communal areas (location to be decided during site set up). The final number of air filters will depend on the number of "consented" residents within each care home, which will in turn partly depend on the size of the care home.
- Energy meters (same number as air filters)
- Extension cords (as required)

6.5 Individual resident invitation

6.5.1 Identification of individual residents

The anonymised study register (see section 6.4.3) will be sent to the study team to create a random order of eligible residents for the care home managers/study champions to approach and invite residents to receive an air filter in their private bedrooms and/or provide access to their medical records and share identifiers with the

UKHSA. If the staff recruiting approach the first residents randomly selected for consent on the list and some decline, the study team will request they work their way down the random list until they have consented enough residents. The average number of residents consented at each care home will be 12. Some larger care homes will be asked to recruit more residents to make up for fewer residents being consented at smaller care homes. The maximum number of residents that will be consented at any care home will be 16.

Residents will have the option to opt out of any data being collected about them (see section 6.15.3). This will be recorded on the register.

6.5.2 Invitation

Trained staff (managers and study champions) will be asked to provide the relevant invitation materials to the randomly selected eligible residents. Resident Information Sheets (RIS; provided in written, audio, picture and video formats) will be provided to residents/consultees to enable them to decide whether or not they would like to be one of the "consented" residents at that site. A letter will be sent to the GP of residents who consent to take part in the study.

If a resident is selected that does not have capacity, a suitable Consultee will be identified. This will either be a Personal Consultee (relative or close friend), or a Nominated Consultee (someone who is not a member of the care home staff and who is independent of the study). If a suitable Consultee cannot be identified, the resident will not be approached to be one of the "consented". See section 7 for further information.

6.6 Consented residents baseline data collection

For eligible and consented residents, the following baseline data will be collected on an individual level: name; date of birth; sex; ethnicity; NHS number; receiving nursing care, infection symptoms.
Study champions will ask eligible and consented residents to complete the resident infection prevention and control & care home satisfaction questionnaires (see section 6.11).

Consented residents in the intervention arm will receive an air filter for the winter period. For further information regarding consent procedures, see section 7.

6.7 Resident data collection during winter period

Data will be collected on up to approx 30 eligible residents ("the 30") in the winter months (usually defined as 1 September to 31 May- assuming COVID-19 is still prevalent). The pool of "the 30" residents will be randomly selected from all eligible residents on the resident register. The random selection will be carried out either via the study database or, if required for technical reasons, by the study statistician. If a resident in the pool of "the 30" opts out of the study, another randomly selected eligible resident will be selected to replace them. Where the care home has ≤30 eligible residents, data will be collected for all eligible residents. During the winter period, the air filters will be switched on in the intervention care homes (following 2 days of data collection at baseline). Data may be collected on a paper CRF, on a daily basis and entered into the database at the end of each week or entered directly into the database on a daily basis.

Data collection will begin once sufficient residents have been approached and consented in both control and intervention care homes. Usually "sufficient" residents would be considered to be at least 7. Every effort will be made to consent residents as soon as possible in each care home for both groups. Data collection should start no earlier than the 1 September.

For the intervention care homes, data collection should start no earlier than the 1 September. Two days of data collection is required before the air filter units are switched on.

The resident register (section 6.4.3) will be kept up to date throughout the care home data collection period with resident eligibility and basic information. Resident absences from the care home will be recorded. Residents joining the care home will be added to the register up until 1 May.

6.7.1 Data collected on up to 30 ELIGIBLE residents in intervention and control arms

The following data will be collected on up to 30 eligible residents in an anonymised format (unless they opt out of the study);

- **Infection symptoms** (Frequency: Daily): Any new (or worsening of pre-existing) respiratory symptoms including: runny/blocked nose; sneezing; sore/tickly throat; earache; runny ear; red/sticky eye/s (part of the respiratory tract due to the lacrimal duct); hoarse voice; cough; wheeze; noisy breathing; sputum (phlegm); chest pain; and/or shortness of breath, loss and/or change in taste/smell, vomiting, diarrhoea, delirium and/or confusion, deterioration in physical ability, fever (highest temperature).
- **Antibiotic consumption** (Frequency: Daily): Study champions use whichever data source is most appropriate, including direct discussion with the resident and/or consulting care home drug charts. For residents with capacity, where the drug chart and resident disagree, the resident's view will take priority.
- **Frequency of adverse events** (Frequency: Daily): Any falls or near falls using PROFANE (Prevention of Falls Network Europe consensus, see http://www.profane.eu.org/) definitions respectively for falls as ''an unexpected event in which the participants come to rest on the ground, floor, or lower level''.

6.7.2 Data collection on ALL CONSENTED residents in intervention and control arms

- **Infection prevention control strategies questionnaire** (frequency: end of March): see section 6.12.
- **Care home satisfaction questionnaire** (frequency: end of March): see section 6.12.
- **Additional care** (frequency: daily): The resident register for consented residents will include details of additional care received outside of the care home (e.g. primary care contacts).
- **End of follow-up medical notes review.** The central study team will contact the relevant GP practice for those residents and ask them to provide data between baseline and end of follow-up. Data will include: medically diagnosed infections (respiratory, urinary, gastrointestinal and skin/soft tissue infections); number and names of antibiotic prescriptions, hospital admissions*, adverse events and GP consultations.

6.7.3 Data collected on CONSENTED residents in INTERVENTION arm only

- **Air filter Satisfaction questionnaire** (frequency: end of March): see section 6.12.

6.8 Care home follow up data collection

Care home level follow up data will commence as defined in section 6.7.

6.8.1 Care home follow up data collection in ALL care homes

- **Infection prevention control strategies questionnaire** (frequency: end of March): see section 6.10.
- **Care home satisfaction questionnaire** (frequency: end of March): see section 6.10.
- **Staff absenteeism** (frequency: daily/weekly) – managers will anonymously report absenteeism, number of days absent and reason for absence e.g. whether it was related to an infection.
- **Staff roles and numbers** (FTE, permanent and agency); funding (private/public) (collected at baseline and at the end of March). Care homes will also be asked to record any changes in staffing levels and funding since baseline.

6.8.2 Care home follow up data collection in INTERVENTION care homes

- **Air filters satisfaction questionnaire** (frequency: end of March): see section 6.10

6.9 Air filter data collection

All air filters will be assigned a unique identifier. Locations of each device will be recorded and a record of whether the device is being used in an individual bedroom, staff only area or communal area will be recorded.

If a consented resident in the intervention group moves room, their assigned air filter will be relocated to their new room. See also section 6.15.2.

6.9.1 Data collection in ALL care homes

- **Use of additional air filters in communal, staff and private bedrooms** Care home managers will record if they have any new air filters (acquired outside of the study) in the care home since baseline and if so, the number of communal, staff or private resident bedrooms with air filters and whether they have been switched on (see section 5 for eligibility criteria).

6.9.2 Data collection in INTERVENTION care homes

- **Fidelity data:** Intervention study champions/ managers will record the following data relating to the use of all (both private and communal room) air filters
    - Fidelity measures: checklist regarding location as planned (including whether residents with air filter in private bedrooms have the filters assigned to them (frequency; weekly), switched on (frequency: daily), electricity use (frequency: weekly).
    - Filter maintenance (frequency: weekly) air filter unit is cleaned, filter replacement requirement checked.

6.9.3 Data collection in CONTROL care homes

- **Installation and use of new air filters in private rooms for consented residents only (frequency: daily) (see 6.7.4)**

6.10 Staff questionnaires

Staff will complete the questionnaire via an online survey or on paper. Staff will be provided with freepost envelopes to send their completed questionnaire to the central study team for entry onto the database. Staff that are involved in consenting residents will not be asked to complete the questionnaires.

6.10.1 Infection prevention control strategies questionnaire (IPC) (frequency: baseline and end of March)

Staff will be asked to report their beliefs and confidence in IPC using 5-point Likert scales (strongly agree/ agree/ not sure/ disagree/ strongly disagree) in response to the following statements:

- Lack of time prevents me from always following infection prevention and control procedures.
- I am confident my use of infection prevention and control procedures is effective.
- I believe infections can be spread through the air, for example as droplets from a sneeze or cough.
- I believe air filters reduce infections being spread through the air.

6.10.2 Care home satisfaction questionnaire (frequency: baseline and end of March only)

Staff will be asked to completed a care home satisfaction questionnaire using 5-point Likert scales (very satisfied/ satisfied agree/ not sure/ dissatisfied/ very dissatisfied) about their overall satisfaction with the care home environment (e.g. air temperature, odours).

6.10.3 Air filter satisfaction questionnaire (frequency: intervention care homes/end of March only)

All care home managers and staff will be asked to report their satisfaction with air filters using 5-point Likert scales (very satisfied/ satisfied agree/ not sure/ dissatisfied/ very dissatisfied) for all air filters (staff rooms, communal rooms and private bedrooms).

6.11 Resident Questionnaires

6.11.1 Infection Prevention Control Strategies Questionnaire (frequency: baseline and end of March)

Residents will be asked to report their beliefs and confidence in IPC using 5-point Likert scales (strongly agree/ agree/ not sure/ disagree/ strongly disagree) in response to statement such as the following:

- I believe infections can be spread through the air, for example as droplets from a sneeze or cough.
- I believe air filters reduce infections being spread through the air.

6.11.2 Care Home Satisfaction Questionnaire (frequency: baseline and end of March)

Residents/consultees will be asked to complete a care home resident satisfaction questionnaire using 5-point Likert scales (very satisfied/ satisfied agree/ not sure/ dissatisfied/ very dissatisfied) about their overall satisfaction with the care home environment (e.g. sleep quality, air temperature, odours). The approximate amount of time residents spend in communal areas will also be captured.

6.11.3 Air filter Satisfaction Questionnaire (frequency: intervention care homes/end of March only)

Residents/consultees will be asked to complete a resident air filter satisfaction questionnaire about their overall satisfaction of the air filter in their bedroom using 5-point Likert scales (very satisfied/ satisfied agree/ not sure/ dissatisfied/ very dissatisfied).

6.12 UK Health Security Agency (formerly Public Health England) (collected retrospectively)

UK Health Security Agency (UKHSA – formerly Public Health England) data regarding outbreaks within the care home will be collected in an anonymised format for the whole care home (this will include residents who opt out due to the anonymous format). This data is expected to include:

- SARS-CoV-2 PCR testing: this data is collected by the care home when a PCR test has been requested. The results of this test will also be known.
- Other respiratory virus testing: this data is also a requirement to be collected by the care home for cases of influenza A and B and other respiratory viruses.
- Other infections: This includes Streptococcal, Meningococcal, Respiratory Syncytial Virus, Norovirus and Human Metapneumovirus infection testing which will also be required by the care home to provide this data to UKHSA.
- With consent, the research team will share identifiable information about the “consented” (which may include initials, NHS number, full name, date of birth) with UK Health Security Agency (formerly Public Health England) so that they can provide the University of Bristol with relevant information about study outcome measures, for example infection outbreaks in the care home and mortality data.

6.13 New residents

New residents joining the care home will be informed of the study and will be added to the anonymised resident registry. They may be added to the randomly selected pool of “the 30” residents for data collection / potentially invited to consent if any of “the 30” opt-out of the study. Where care homes have ≤30 residents, data collection will commence on new residents unless they opt out of study involvement, or they become a resident in the care home after the after 1 May, since they will not be able to contribute at least 1 month of data collection.

6.14 Qualitative outcomes

See section 8.

6.15 Change in participation status of care homes and participants

In all instances, data collected up to the date of the change/opt-out will be retained by the trial in accordance with GDPR regulations.

6.15.1 Care home change in participation status

Any care home can request to change their participation status in the study at any time. The care home can change their participation by requesting to stop any of the following:

i) Air filters in resident communal areas
ii) Air filters in staff communal areas
iii) Air filters in resident private bedrooms
iv) Data collection for residents
v) Data collection for staff
vi) Complete discontinuation of all trial procedures. If this is decided, the care home will end all involvement in the trial, including activities associated with the individually consented residents. If a care home does wish to withdraw completely, they will be required to complete a study change in participation status form and will need to arrange return of the air filters (intervention homes only).

Note: If a care home changes their participation status, permission will be sought to continue with the GP notes review for individually consented participants).

6.15.2 Resident change in participation status

Consented residents can request to change their participation status in the study at any time. They can change their participation by requesting to stop any of the following:

i) Having an air filter in their private bedroom (intervention residents only)
ii) Access to their medical records

Even if a resident decides to change their participation by no longer having an air filter in their bedroom or allowing access to their medical records, they can still contribute data to the daily anonymised data collection elements (unless they opt-out of this).
If a consented resident changes their participation in the study between the period 1st Sept to 1st May, a replacement resident will be sought following the procedures in sections in section 6.5; consent to join trial, baseline data collection and follow up. If a resident in the intervention group decides they no longer want an air filter in their private bedroom, the newly consented resident will be offered the same shell air filter unit but the study will supply a new inner air filter. Used air filters may be sent to the laboratory for storage as detailed in section 10.7.

6.15.3 Resident opt-out

Throughout the set-up of the trial at the care home and during follow-up, if a resident (or a consultee) decide they do not want their data included in the trial, they can opt-out of the anonymised data collection at any time from their data being collected from baseline through to end of follow-up. This will be recorded on the trial resident register by the care home staff and if a reason for the opt out is given, this will be recorded.

6.16 Care home closedown

6.16.1 Care home study paperwork, tablets and electricity monitors

At the end of the winter period, care home managers will ensure all study paperwork is logged online and any hard-copies are returned via secure post to the University of Bristol. Similarly tablets, electricity monitors and extension cords will also be returned to Bristol.

6.16.2 Removal of air filters

At the end of the care home data collection period, care home staff may be asked to remove the filters from the air filter unit. If this is the case, they will use the materials provided to label and package the filters and arrangements will be made to send the used filters to the research laboratory (see section 10.7).

The remaining ‘shell’ units will be offered to the care homes and residents for use with new filters that they would be responsible for purchasing. If, for any reason, the used filters are not required by the study team, appropriate arrangements will be made with the individual care homes for the used internal filters (e.g. local disposal according to manufacturer guidance).

6.16.3 Thanking care home staff for their involvement

At the end of the care home data collection period, the study team will provide ‘study certificates’ for all care home managers and study champions.

6.16.4 Thanking residents for their involvement

The study team will provide a large colour poster to hang on the communal room wall thanking the residents for their help with the study. In addition, the study team will provide consented residents with a thank you card.

The study team will contribute to an appropriate gift/vouchers as guided by the PAG group and care home managers.

6.17 Methods/procedures to protect against other sources of bias

Randomisation will be at care home level stratified by nursing care provision and socioeconomic status, because infection transmission occurs within care homes between staff and residents, and we anticipate individual use of air filters could benefit non-users.

To minimise post-randomisation bias, the care home/care home staff will complete baseline care home data prior to randomisation. Where possible, staff questionnaires will also be completed before data collection starts.

Previous research suggests ~30% of residents will wish to take part and ~40% will leave the care home or die during the study,(34) reflecting the frailty and vulnerability of the study population. To maintain study power, we will continually recruit new residents, resulting in an expected overall study dropout ~20%. Sensitivity analyses will be used to investigate the potential impact of this strategy, including checking for evidence of post-randomisation recruitment bias.

Primary outcome attrition will be minimised through the use of short, simple-to-complete, staff administered symptom questions.

The process evaluation will assess if use of air filters results in any unintended relaxation of infection prevention and control procedures.

6.18 Contact procedure for care homes

Details of what a participant/care home should do if they experience any problems whilst taking part in the trial are detailed in the AFRI-c information sheets and care home training / FAQs on the study website. Most study activities will be conducted electronically. All care homes will be required to maintain a small paper-based study file which will include the contacts for the Bristol-based study team who will be available to respond to queries during normal office hours.

6.19 Blinding

The central research team, clinicians, other researchers, site staff and participants will not be blinded to the allocation of intervention group. The Trial Management Group (TMG) will not review unblinded data until all follow-up and data queries have been resolved at the end of the trial. Two statisticians based at the University of Bristol (UoB) will support this trial. The lead statistician will be blinded throughout the trial. The trial statistician will perform all disaggregated analyses according to a pre-specified SAP and will attend closed Data Monitoring Committee (DMC) meetings as required. Two health economists based at UoB will be blinded when cleaning data and preparing the analysis plan, but the junior health economist will be unblinded when conducting the analysis.

6.20 End of trial

Participant: The participant ends their involvement with the trial when their last assessment (including medical notes review for consented residents) are completed (or they have changed their participation status/opted-out from the study).

Trial: The end of trial for AFRI-c will be when the last resident has completed their follow-up, which includes completion of the medical notes review, all data queries have been resolved and the database has been locked, with subsequent data analysis completed.

6.21 Trial stopping rules

The trial may be prematurely discontinued by the Sponsor, Chief Investigator (CI), Regulatory Authority or Funder based on new safety information or for other reasons given by the DMC / TSC regulatory authority or ethics committee concerned.

The trial may also be prematurely discontinued due to lack of recruitment or upon advice from the TSC, who will advise on whether to continue or discontinue the trial and make a recommendation to the Funder. If the trial is prematurely discontinued, no new participants will be recruited, and a decision on data collection on active participants will be made in discussion with the TSC/DMC and Sponsor.

7 CONSENT

AFRI-c consent training documentation will describe all the details of consent processes, including which activities should be conducted by which members of care home and study staff.

7.1 Overview

Infection symptoms, antibiotic consumption and any falls or near falls will be collected on up to 30 randomly selected eligible residents (that do not opt out of study involvement) without obtaining individual consent. UK Health Security Agency (UKHSA – formerly Public Health England) data about outbreaks among all care home residents and staff will also be collected. These datasets will be provided by the care home and UKHSA in a fully anonymised format for all residents. In addition, for the "consented" residents we will collect consent to share identifiers with the UKHSA (full name, date of birth, NHS number) to obtain identifiable information for secondary outcomes. A study information poster will be displayed around the care home and given to residents where appropriate. This will describe a summary of the study, the aims, the details of the data being collected about residents and the option to opt-out of the study (see section 6.15.3).

Names of all residents will be collected on the care home register and stored securely within the study specific database on the University of Bristol servers. The resident names will be encrypted so are only viewable to care home staff / consenting teams and not readily available to the rest of the study team. These will only be held for the minimum amount of time required for the study processes. The study team will be able to see names of consented residents / consultees on the consent / declaration forms.

For the residents selected to be approached at random (see section 6.5.1) in each care home, the full consent process described below will be followed.

Care home staff will be informed about the study and given the opportunity to complete the Infection Prevention Control strategies questionnaire (IPC) and care home satisfaction questionnaire at baseline and at the end of March. Intervention care home staff will also be given the opportunity to complete the Air Filter Satisfaction questionnaire at the end of March. Individual consent from staff will not be sought for questionnaire completion; this will be implied by the completion of the questionnaires.

7.2 Consent of individual residents

A summary of the various study invitation and consent pathways are shown below in Figure 2. Individual residents selected to be approached to participate in the study will be given an AFRI Resident Information Sheet (AFRI-RIS) (for residents that have capacity to provide informed consent). If a resident is selected that does not have capacity, a suitable Consultee will be identified. These individuals will be given an AFRI - Consultee Information pack (AFRI-CIS). The information sheets provided will be specific to the allocation of the care home (Intervention or Control i.e. there will be specific information sheets described above, tailored to the study procedures taking place in the intervention and control arms

Residents will be invited to consent to:

- In both intervention and control care homes-
    - Allow access to their medical records (regarding baseline medical conditions and their treatments, and then subsequent infections, antibiotic prescribing and hospital admissions) for data collection purposes;
    - Complete study satisfaction questionnaires;
    - Allow the study team to share their identifiers with UKHSA (as described in section 6.12)
- In the intervention homes only-
    - Have an air filter in their private bedroom (with subsequent data collection about the air filter).

Informed consent will remain unless the individual requests to change permissions, in which case the relevant procedures will be followed.

7.2.1 Residents with capacity to consent to take part

In line with the Mental Capacity Act (2005), residents in the care home will be assumed to have capacity to take part in the study unless established otherwise. Randomly selected residents with capacity will be given the AFRI Resident information sheet (AFRI-RIS). They will be given sufficient time to read the information sheet and ask any questions they may have about the study. The informed consent process will be undertaken by appropriately trained care home staff or a researcher from the central study team, or CRN delivery teams. All members of the care home staff delegated to undertake the consent procedure must be listed on the study roles and responsibilities log at the relevant site and have completed site-specific training (incorporating GCP training), the study research nurse or delivery teams will also assess their ability to consent.

In the event that a resident loses capacity to take part during their involvement in the trial, we will identify a suitable Personal Consultee (or Nominated Consultee if a Personal Consultee is not available) to provide advice on the resident's continued involvement in the study as soon as is feasible. If a Consultee cannot be identified, or advises that the resident should no longer take part, the resident will stop participation in the study, as one of the "consented" (as applicable). Where participation stops, any data collected up to that point will be retained. Care homes will be given up to four weeks to identify a Consultee before a new resident is randomly selected to be approached.

7.2.2 Residents without capacity to consent to take part

We are committed to involving a representative sample of residents, including those with issues that may affect their decision making capacity, in order to ensure the results are generalisable. We acknowledge that a resident's cognitive impairment may relate to a neurodegenerative disease, such as dementia, which is likely to remain throughout the duration of the trial due to its chronicity. Alternatively, resident's cognitive impairment may be due to an acute medical condition or emergency, such as delirium, and may therefore be temporary. These residents may lack capacity to provide consent for themselves if they are unable to:

- understand the information relevant to the decision
- retain the information
- use or weigh the information
- communicate their decision (by any means).

If a potential participant is identified as lacking capacity to provide consent for themselves at the time a decision or action needs to be taken, the Central Research Nurse or delegated individual with relevant experience will take reasonable steps to seek an opinion from a Consultee. If possible, this will be a Personal Consultee (e.g. a close relative or friend). In the event that the person lacking capacity does not have a suitable friend or relative who is willing to act as a Personal Consultee, a Nominated Consultee (who is not a member of care home staff and who is independent of the study) will be sought. In accordance with the Mental Capacity Act 2005, Consultees will be asked to advise whether the resident would wish to participate if they had capacity. In accordance with the guidance, examples of people who would be considered suitable to be a Nominated Consultee are: someone with a professional relationship with the person (e.g. doctor, solicitor, social worker), a spiritual advisor, or an appointed Independent Mental Capacity Advocate (IMCA)

The majority of care homes within the UK have imposed visitor restrictions in light of the (ongoing) COVID-19 pandemic. In order to facilitate timely and appropriate participant recruitment with Consultee approval, discussion of the trial will be made via telephone (or alternative method if requested by the Consultee and where it is appropriate and feasible, e.g. Sponsor/care home-approved video/tele-conference platforms) in the first instance with a AFRI-c Consultee Information Pack (either control arm or intervention arm information sheet, depending on care home allocation, and either Personal or Nominated Consultee pack – depending on the person). This will be made available to the Consultee, either by hand (at the care home) or sent by email or post if required.

Ideally the Consultee will have discussed and/or read the AFRI-c Consultee Information Sheet before providing informed advice. Advice from a Consultee about whether the named resident would wish to be included in the trial or not, can be obtained via e-consent/ telephone/ fully written consent (see section 7.3)

If someone declines the invitation to be a Consultee, they will not be asked to sign anything, however the research team will record, in the relevant study documentation and resident records, that the person was asked and said no; this is so that they are not asked again about this role in the future.

If reasonable steps to contact a Personal Consultee have failed (e.g. where no family member or friend is willing and able to act as consultee, or where the family or friend live a long distance away, and/or are unable to at least discuss the information sheet(s) within adequate time), and/or a Personal Consultee becomes unavailable during the study, or is no longer willing to undertake the role, then a Nominated Consultee will be sought.

Informed advice will remain, unless the individual requests to change their participation status in the study, in which case the relevant procedures will be followed. If the Consultee advises the research team that the participant should stop taking part in the study, the research team must stop their participation in the study, but any data collected up to that point will be retained.

Where appropriate, and if permitted by the Consultee, the research team will invite collaboration in the completion of any questionnaires or research procedures. The ability of residents with mild to moderate cognitive impairment to complete study related questionnaires is an important outcome in terms of completeness of data.

- **Residents who regain capacity**

If a resident regains capacity to consent during the trial period, then the central research nurse (or staff from delivery teams) will ask them to give their own ongoing consent when and if they are able. Such residents will be provided with the appropriate “Recovered Capacity Resident Information Sheet”, that explains what has happened so far and what we are seeking their consent for. Where feasible, no further trial assessments and/or procedures will take place until after consent from the resident has been obtained. If the resident advises that they no longer want to take part in the study the resident’s participation in the study will be stopped. Consultees will be informed of this consent process, following regained capacity, at the outset via the AFRI-Consultee Information Pack.

7.2.3 Follow-up

Once a resident is consented, capacity assessments will not be re-performed during the trial period unless there is reason to believe the resident’s capacity has changed.

Figure 2: Study invitation and consent pathways depending on capacity status.

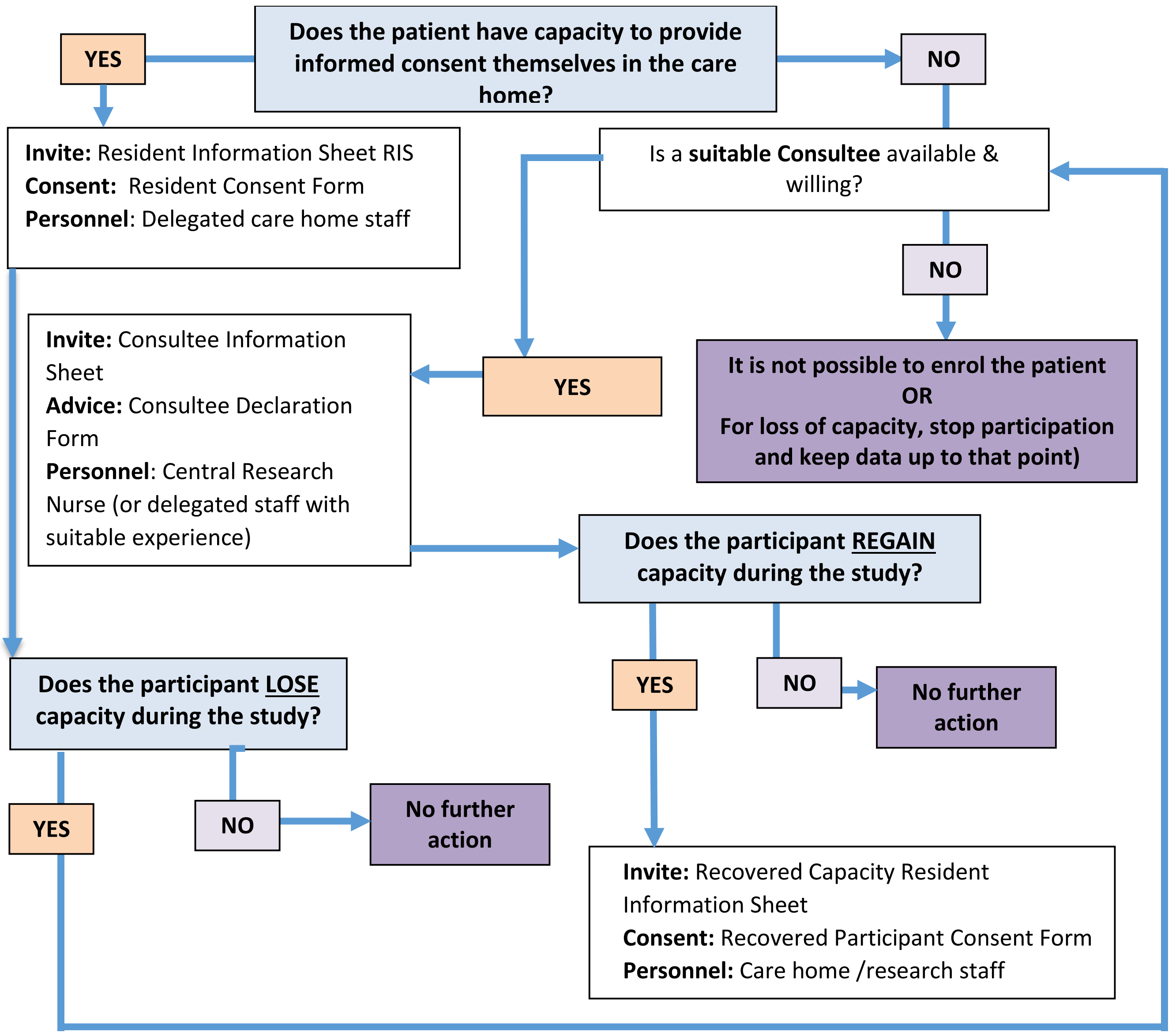

7.3 Methods of consent

Irrespective of the method used to record consent/advice (as described below), 5 copies of the consent/declaration form will be required: (1) to be provided to the resident/consultee with a copy of the information sheet; (2) to be filed with a copy of the information sheet in the participant's care home records; (3) to be filed in the ISF; (4) to be provided to the central trial team (study office) and (5) to be provided to the resident's General Practitioner (GP).

Besides completing the consent form (which includes the study title and date of consent), sites should record key details of the informed consent process in the resident's care home notes. Residents are not required to provide reasons for taking part in the study, or not, but if reasons are given, then they should also be documented in their notes.

7.3.1 Face to face, consent consultation

Residents (or consultees) will be given the relevant study information sheet and be given adequate time to read the information. During the face to face consultation the resident (or consultee) will be provided with the opportunity to understand the nature, significance, implications and risks of the trial in order that they may make an informed decision about whether to take part or not (or provide advice that the resident would wish to take part).

If the resident/ consultee wishes to participate, written consent will be received on the Consent Form/Declaration form. This should be completed and signed by the resident/consultee, dated and countersigned by the individual who is taking consent. If the resident / consultee finds writing difficult then eConsent / eDeclaration may be used (see section 7.4.1).

7.3.2 Remote consent consultation

To support remote consent mechanisms, informed consent consultations may take place over the phone or via an approved University or Care home approved video conferencing facility. Residents (or consultees) will have been sent the relevant study information sheet and been given adequate time to read the information prior to the consultation. During the consultation interview, the potential resident (or consultee) will be provided with the opportunity to understand the nature, significance, implications and risks of the trial in order that they may make an informed decision about whether to take part or not (or provide advice that the resident would wish to take part).

7.4 Documenting consent

Where the resident has capacity but is unable to indicate their consent by or marking a document then their consent may be given orally in the presence of at least one witness and recorded in writing.

7.4.1 eConsent /eDeclaration

Remote consultations may be documented utilising an e-consent mechanism; as this is a low risk non-CTIMP study which involve risks no higher than that of standard medical care (and following the HRA and MHRA joint statement on seeking and documenting consent using electronic methods (eConsent) (Published 24Sep 2018). The relevant study consent/declaration form will be presented in an electronic format and a simple electronic signature will be used to record consent (tick box for each statement, a typed name, and declaration). eConsent/eDeclaration will not be followed up with a paper (wet ink) written consent form as an electronic signature constitutes documented informed written consent. A copy of the eConsent/eDeclaration form will be made available to the resident/Consultee.

7.4.2 Verbal consent/ verbal declaration

If verbal consent is taken over the phone or during a video consultation, where possible, two copies of the relevant blank consent form will be sent with the information that can be completed during the call. Where this is not possible, a study-approved verbal consent/declaration form completed by the researcher during a telephone or video consultation with the resident/consultee will be utilised.

This will be followed up with a written consent/declaration form or eConsent/eDeclaration form. To obtain written consent via post (only after verbal consent has been given) the study staff should sign two copies of the written consent form and post both copies, along with a copy of the completed verbal consent form, to the resident/consultee. The resident/consultee should complete and sign both copies of the written consent/declaration form. The resident/consultee must then send one copy (e.g. either via post/email or by uploading it to the database via the care home) of the completed written consent/declaration forms back to the study team and keep the other copy of the written consent/declaration form and the completed verbal consent/declaration form for their records.

8 MIXED METHODS PROCESS EVALUATION

The main aim of the mixed method process evaluation is to understand acceptability and whether and how the intervention is implemented within the trial and to contribute to the interpretation of effectiveness findings. The process evaluation will comprise of 1) quantitative data from staff and resident questionnaires, 2) qualitative interviews and 3) a sustainability stakeholder workshop. Qualitative data will be collected in parallel throughout the duration of the study and be merged with the quantitative findings during analysis and interpretation. These findings will inform the sustainability workshop. Preliminary findings from the qualitative data collected during the pilot phase will be reported to the TMG to help inform progression criteria, refinement and optimisation of study processes and intervention implementation within the main trial phase.

The process evaluation will be informed by the Theoretical Framework for Acceptability (TFA) (35) and Normalisation Process Theory (NPT), (36-38) which was developed to explain the processes leading to routine embedding of interventions in health care settings. The TFA will inform the acceptability constructs to be assessed including affective attitude, burden, ethicality, intervention coherence, opportunity costs, perceived effectiveness and self-efficacy. NPT proposes that implementation of the air filters in care homes will be dependent four constructs which are

- 'Coherence' (how people make sense of the intervention and their roles and responsibilities in using and maintaining the intervention both individually and collectively. Perceived value of the intervention including perceived and experienced benefits and harms),
- 'Cognitive participation' (the work people do to develop and maintain new practices, is there buy in from participants for the intervention to work? Does it align with care home priorities?),
- 'Collective action' (the work to operationalise practices, Interactional Workability (people communicate and work together to deliver the intervention within their setting), and
- 'Reflexive monitoring' (ways in which people assess and understand the ways in which the intervention and new ways of working affect them and others around them, how new practices are working, and potential/actual changes required to implement and maintain the intervention, assessment of worth of intervention and impact on other tasks).

NPT will be used to explore implementation processes involved in the delivery of the study and intervention and sustainability of the intervention.

Based on team experiences in Winter 1, we will also carry out 5-10 interviews with staff in control arm care homes. The aim of these interviews will be to understand the challenges of and facilitators to data collection in the usual care homes, and also to explore if these homes and/or their residents have purchased air filters (contamination). These interviews will be with staff only and will take place in Winter 2. Findings will be fed back rapidly to the TMG, with the aim of implementing any potential improvements that are identified.

8.1 Logic model

The logic model for the air filter intervention represents the process of the intervention, and the outcomes being assessed. The context in which the intervention is delivered (resident and care home characteristics), potential mediators and moderators and the impact these may have is also considered. See Figure 3.

8.2 Quantitative data (questionnaires)

Quantitative data will be collected through the resident and staff questionnaires at baseline and in March. (see table 3). This includes the

- Infection prevention and control strategies questionnaires (residents and staff)
- Satisfaction of care home environment questionnaires (residents and staff)
- Resident air filter satisfaction questionnaire (residents)
- Staff air filter satisfaction questionnaire (staff)

8.3 Qualitative Interviews

Semi-structured interviews with care home staff, residents and their relatives/friends will explore perceptions and experiences of using the air filters including perceived/experiences benefits and harms, intervention

acceptability and intervention implementation process including context- intervention interactions and dependencies with reference to the core NPT constructs (as outlined above). Within the pilot phase we will also explore feasibility and acceptability of trial processes including consent pathways. Interviews will be conducted by the study qualitative researcher.

Interview topic guides will be used to ensure similar topics are covered in each interview but applied in a flexible manner to enable issues of importance to the participants to emerge. Topic guides will be informed by study aims and previous literature and be developed with PPI input. Interviews may be conducted over the telephone, via a secure online platform or in person dependent on participant preference and COVID restrictions at the time of interview. Interviews will be audio-recorded with informed consent using encrypted audio recorders. Audio-recordings will be transcribed verbatim by members of the study team or a University of Bristol approved transcription company.

Maximum variation/ purposive sampling (39) will be used to select participants encompassing: (i) size/type of care home; (ii) characteristics of residents (e.g., age, level of care, ethnicity, agreed/declined); and staff role (e.g., providing personal care, care home managers). Up to 45 care home staff, residents and relatives/friends will be interviewed (15 during the pilot study and 30 during the main trial phase). The final sample size will be determined by 'information power' relevant to study objectives (40, 41). Some participants may be interviewed at more than one point, for example to explore trial processes and participant decisions in the early stages and later to explore acceptability and intervention implementation or decision to stop taking part in the study.

8.3.1 Identifying and consenting participants

Care home staff, residents and relatives/friends will be informed about the interviews when the study is introduced (tea parties, videos, leaflets).

Residents/relatives

Residents (or their consultees) and family/friends of residents being invited to consent to have the air filters in private bedrooms (the "consented") will be informed of the interviews through the resident/consultee/family or friends information sheets. If they are happy to be contacted for an interview, they will indicate this on resident/consultee consent/declaration forms. Residents will be interviewed only if they have capacity at the time of being invited. If a resident does not have capacity at this point, their consultee will be invited to be interviewed. If the resident's capacity fluctuates between the time of consent and the time of qualitative interview (i.e. regains capacity or loses capacity) the process described in section 7 will be followed.

Those who agree to be contacted and who are selected to be invited for an interview will be contacted by the qualitative researcher who will explain more about the interview, answer any questions and if the resident/consultee agrees arrange a convenient time and preferred method to conduct the interview. Verbal consent (where the resident/consultee agrees to standard consent form statements at the start of the qualitative interviews) will be audio recorded and obtained by the qualitative researcher who will be GCP trained. Verbal consent for the qualitative interviews will not be followed up with written consent.

Care home staff

Initial interest in potentially taking part in the interview and agreement for names and contact details to be passed to the qualitative researcher will be recorded by the study champions. Those who agree will be contacted by the qualitative researcher who will provide the *'staff interview information sheet'* (if not already provided), explain more about the interview, answer any questions and if they agree arrange a convenient time and preferred method to conduct the interview. Consent for the interviews will be obtained by the qualitative researcher verbally using the same process outlined for residents/consultees (as above)

8.3.2 Qualitative data analysis

Anonymised interview transcripts will be analysed using reflexive thematic analysis (42, 43). Transcripts will be coded for key categories and concepts, using deductive coding (using a priori codes, based on the research

aims and NPT concepts) and inductive coding (developing new codes based on issues emerging from the data),(42, 44) with the aid of Nvivo software. Analysis will be ongoing and iterative with interviews and analysis proceeding concurrently. Analysis will be led by the qualitative researcher, with a sub-set of data independently coded by the qualitative lead to enhance trustworthiness of the analysis process and to contribute to theme development. Any discrepancies will be discussed and incorporated into the final coding framework.

8.4 Data management, protection and patient confidentiality in relation to the qualitative research data

Interviews will be recorded using an encrypted audio recorder and transferred as soon as possible after the interview to secure UoB storage. The recording will then be deleted from the recorder. If recordings are made through a video conferencing platform then only the audio-recording file will be transferred securely to the UoB and both the audio and video files will be deleted from the video-conferencing platform. Audio-recordings of interviews will be transcribed in full by a UoB member of staff or UoB approved transcription service that has signed the necessary confidentiality agreements. Audio-recordings and transcripts will be labelled with a study I.D number, and stored securely adhering to the University’s data storage policies. Transcripts will be edited to ensure anonymity of respondents. Anonymised quotations may be used for training, teaching, research and publication purposes for this and future studies. Anonymised transcripts may be made available by controlled access to other researchers who secure the necessary approvals for purposes not related to this study, subject to individual written informed consent from participants. Excerpts of audio-recordings concerning verbal consent will be retained for auditing purposes in line with trial archiving policies. The remaining content will be deleted at the end of the study.

8.5 Safeguarding residents during interviews

We will ensure that participants are not subjected to undue distress during the qualitative interviews. To mitigate this, and the possibility that participants may disclose information to provoke concern about risk, the interviewer will be an experienced qualitative researcher who will adhere to the following:

Participants will be informed that the interview is strictly confidential, but should they disclose information to suggest that they or others are at significant risk of harm, the interviewer will discuss this with a clinical advisor and may need to disclose these details to the designated safeguarding authority. The interview will only continue if participants are happy to proceed and engage with the interview topics. If the researcher feels a participant becoming tired, confused or distressed, they will ask the participant if they wish to have a break or discontinue the interview (with the option to continue later) and will offer support. Resident participants will be asked if they would like someone to be present during the interview. To help build a relationship and rapport with residents the researcher will speak with the resident before the interview is conducted. A safeguarding protocol for researchers and participants will be implemented, and participants will also be provided with a leaflet with the contact details of support networks. If in person interviews are conducted the qualitative researcher will adhere to the latest University of Bristol lone working guidance and current COVID-19 restrictions/guidance.

8.6 Sustainability Workshop

A workshop will be held with care home providers, CCG and Local Authority commisioners and national policy makers to discuss intervention maintenance and sustainability outside of the trial context. The workshop will take place once data collection, analysis of the process evaluation questionnaires, qualitative interviews and health economic interviews have been conducted. Findings from these components will help to inform discussion at the workshop. This will likely include scenarios/models of future implementation including potential funding mechanims. The workshop will be held online/virtually.

Figure 3. AFRI-c logic model

Resident and care home characteristics
Resident health and socio-demographic factors Consented residents only: age; gender; ethnicity; receiving nursing care; care home satisfaction and infection, prevention and control strategy questionnaire. All residents: infection symptoms, frailty, and antibiotic consumption.
Care home factors self-reported: area-level deprivation (IMD); residents (overall number, those requiring nursing care, those with dementia, number of males/females, age, ethnicity); number of staff/roles; number of rooms (staff only, resident communal/private); funding (public/private); air filter usage; ventilation policy (windows/doors open/closed); confidence in, and use of, infection prevention and control strategies; care home satisfaction
Care home mandated external reporting: PHE outbreak reports; CQC IPC audits; CQC rating

Intervention

CARE HOME

Air filter function

Installation
- Central study team and care home manager assessment of number of air filters required for private rooms and communal areas
- Delivery of air filters to care home
- Installation (with electrician supervision if necessary)
  - Location and set up as per manual
  - Switch on (as guided by central study team)

Staff training and instructions
- Where each air filter should be installed
- Which setting to be used and when filters to be turned on
- weekly
  - check labelled unit in correct location and switched on
  - record electricity meter value
  - check filter change needed
  - cleaning of air particle sensor
  - cleaning of external filter surface
- Gov.uk infection prevention and control measures

Mediators and moderators

Air filter function

Viral dimensions range between 20–30nm, and bacteria 500–10,000nm (SARS-CoV-2 ~100nm). They become airborne via talking, coughing or sneezing generating aerosols (≤5000nm) or droplets (>5000nm). Portable air filtration units containing high efficiency particulate air (HEPA or equivalent) filters are designed to trap particles >20nm. Unit are designed for specific room sizes (floor m^2). Based on filtration performance characteristics and PPI preference (sturdy design, quiet mode) we selected Philips^{TM} AC3033 (for communal rooms up to 104m^2) and AC2936 (for private rooms up to 85m^2) units. These contain filters capable of removing particles ≥3nm.

Staff attitude and adherence to infection prevention and control (intervention AND control groups)

Staff will be asked to report their beliefs and confidence in IPC using 5-point Likert scales (scales (very satisfied/ satisfied agree/ not sure/ dissatisfied/ very dissatisfied))

Staff will also be asked to completed a care home satisfaction questionnaire about their overall satisfaction with the care home environment (e.g. air temperature, odours). Air filtration device use in control care homes will be monitored.

Outcomes

Quantitative (1° powered) resident

1°: Respiratory (including PCR confirmed SARS-CoV-2) infection episode
2°:
- respiratory and gastro-intestinal infection days
- fever, delirium and/or deterioration in physical ability
- PCR confirmed SARS-CoV-2 (and any other viral) tests
- medically diagnosed infections, antibiotic prescribing
- days on which antibiotics consumed
- Perception of care home environment
- Infection prevention and control strategies
- Hospitalisations

Qualitative

- care home staff acceptability
- resident acceptability
- perceived/experienced benefits/harms
- barriers/facilitators to implementation
- perceived sustainability

Health economic

- cost per respiratory infection averted
- primary and community health care contacts, antibiotics
- secondary care admissions, length of stay
- care worker absence days

Care home environment assessed using a combination of , interviews and questionnaires) to contextualise trial results

Guidance and policy context
UK Government infection prevention and control and COVID-19 policies

9 STUDY CLASSIFICATION

This study is not classified as a device study. In line with the manual on borderline classification in the community regulatory framework for Medical Devices (Version 1.22(05-2019)) the air filtration units being used in the AFRI-c study are 'Stand alone' / mobile units which are intended to purify or decontaminate the air in individual rooms or areas, which may be moved from room to room in accordance with the perceived need to purify or decontaminate the air in the room.

The air filtration units stand in the room and function independently. They are not connected to individual residents directly (e.g. via the use of a mask) and there is no direct contact with an individual resident. In order for a product to be classified as a medical device, the device must have a direct association with the individual resident. Although maintaining clean air may contribute to keeping a resident in an appropriate environment, this is not considered to be a 'medical purpose'. Air is part of the environment of the resident and its cleanness is necessary in a similar way as for surfaces, walls, floors and other objects which also need to be cleaned and disinfected. Since the air filtration units do not fulfil the definition of a medical device, they are not considered to be medical devices, but are rather products for the general environment. This study therefore falls outside the remit of the MHRA.

10 INTERVENTION

10.1 General information

We are conducting the study using Philips™ devices: AC3033 for communal rooms (≤135m^2) and AC2936/33 for private bedrooms (≤98m^2). Due to supply issues, in Winter 3 up to 17 AC3033 devices will be used in private bedrooms in place of the AC2936/33 model. The AC3033 model has a higher clean air delivery rate and therefore has a more powerful/effective filtration system.

Each has a filter consisting of three layers. The outer layer captures visible particles and requires vacuuming when the red brush indicator light turns on. The inner filters are self-contained, capturing particles ≥3nm, and will not 'leak' viruses or bacteria during vacuuming.

10.2 Care home allocation

Care homes allocated to the intervention group will receive usual care plus air (HEPA) filters and will receive up to 21 air filters each. A maximum of 16 will be placed in the private bedrooms of individual residents who give consent, and five will be placed in communal areas (the location of which will be decided during site set up). Each intervention care home will receive new units for one winter (usually 1 Sept to 31 May).

The central study team will determine how many communal air filters are required in each intervention care home by reviewing floorplans / room dimensions of the care home (provided by intervention care homes in set-up) and discussing use of communal areas with care home staff. .

10.3 Distribution of air filters

Air filters will be shipped by Phillips to a logistic company in the UK. Once the care home has completed site set-up and been randomised to the intervention arm, the central study team will liaise with logistics company to notify them how many air filters are required to be delivered to the care home. Delivery will be directly from the logistics company to the individual care homes.

10.4 Installation and use of air filter units

On delivery care homes will confirm receipt of the air filter units, unpack them and confirm that there has been no damage. Units will be labelled with a unique ID. The units are 'plug in and go' and care homes will be supported by the study team with installation and location of the air filters. The study team will ensure room location and set up is as per manufacturer advice. This process is straightforward, the device is removed from transport packaging and plugged into an energy meter, which plugs into the wall. Energy monitors will record unit operating time. Care homes will be asked to check they can plug in the air filters / energy meters to electrical sockets on receipt, without switching on the filters. Extension leads can be provided if needed.

When data collection starts, care homes will be required to enter 2 days of data collection before the air filters can be switched on. Once the care home have installed both communal and private bedroom the 'plug in and go' air filters, they will be advised to keep the air filter switched on at all times. The advice is to keep the air filters on 1 fixed mode consistently. They may want to adjust to the different modes to decide which one(s) are preferable to them, down to personal preference and whether there are different modes preferred between day and night time.

10.5 Air filter modes and settings

Communal room filters have an auto mode and four manual speed levels (sleep, gentle mode, moderate mode and turbo). It is recommended that the filter should be set on gentle mode (level 1).

The private bedroom filters have an auto mode and three manual speed levels (sleep, gentle mode, and turbo). It is recommended that the filter is set fixed on sleep mode.

10.6 Maintenance of filters

Care homes will be advised to:

- When the red brush indicator light is ON (red) clean the ‘internal filter’ using a vacuum cleaner
- When necessary (such as one a week) wipe the outside of the filter device with a dry cloth (or damp cloth for the fabric section).

10.7 Removal of air filters

Used air filters will be removed from the unit and packaged according to trial specific working instructions if a new internal filter is required. A new filter requirement may be indicated by the air filter unit, or at the end of the winter, the study team may ask that internal filters are returned for analysis.

The processing and analysis of the air filters will be done in consultation with the Public Health Laboratory at Southmead Hospital. Given the size of the internal air filters, initial processing is likely to be required before sections of the filter are stored for any future analysis. This initial processing will take place in a suitable laboratory. At the end of the winter period (when all the internal filters from each intervention care home are returned), it may be necessary to store some of the air filters before processing. Suitable storage will be arranged for the air filters (as required) until they are analysed / identified for disposal.

The analysis of the filters will be covered by a separate protocol. It is possible that not all filters will be processed / analysed. The decision about any analyses will be made by the AFRI-c CI, in consultation with microbiologist colleagues.

When no longer required, the filters will be disposed of safely (at least in accordance with the manufacturer recommendations).

The care home will be offered the opportunity to keep the shell units for future use, on the understanding they will be responsible for future running costs of air filters including electricity bills and repairs.

11 CONTROL

All care homes will be advised to continue providing usual care and to follow usual infection prevention and control procedures according to UKHSA, CQC and any other relevant legal/ regulatory authority guidelines. The following are the only differences between control and intervention group care homes:

- Installation and use of air filters
- Quantitative and qualitative assessments of 'living with' the air filters

12 SAFETY

Serious and other adverse events (S/AEs) will be recorded and reported in accordance with the GCP guidelines and the Sponsor's Research Related Adverse Event Reporting Policy. Participant safety will be monitored by the Trial Management Group (TMG), Sponsor and Trial Steering Committee (TSC) and the trial will be stopped if any indication of harm from using the intervention is found.

12.1 Adverse event terms and definitions

Term	Abbreviation	Definition
Adverse Event	AE	Any unfavourable and unintended sign or symptom that develops or worsens during trial participation, whether or not it is considered to be related to the trial intervention.<br>In all instances, it will be up to the PI of each participating site (or appropriate delegate, e.g. clinician) to determine whether the person's change in health is related to the trial.<br>AEs are not continuous and persistent disease or symptoms, present before the trial, which fail to progress; signs or symptoms of the disease being studied (in this case pulmonary complications or development of frailty syndromes); or treatment failure.
Serious Adverse Event	SAE	Any untoward medical occurrence that:<br>• Results in death<br>• Is life threatening<br>• Requires hospitalisation or prolongation of existing hospitalisations<br>• Results in persistent or significant disability or incapacity<br>• Consists of a congenital anomaly or birth defect<br>• Or is considered by the investigator to be an important medical event<br><br>The term "life-threatening" in the definition of "serious" refers to an event in which the participant was at risk of death at the time of the event; it does not refer to an event which hypothetically might have caused death if it were more severe.<br><br>The definition of hospitalisation is an unplanned overnight stay. Note, however, that the resident must be formally admitted – waiting in outpatients or an Accident & Emergency Department (A&E) would not count as hospitalisation (even though this can sometimes be overnight). Prolongation of an existing hospitalisation qualifies as a SAE. Planned hospital stays would not be counted as SAEs, nor would stays in hospital for "social reasons" (e.g. respite care, the fact that there is no-one at home to care for the resident). Also, if residents had a day-case operation, this would not qualify as hospitalisation. However, if a planned operation was brought forward because of worsening symptoms, this would be considered as an SAE. Hospitalisations for the purpose of the intervention are an exception to SAE reporting unless complications occur.

12.2 Classification of severity

Mild event:	An event that is easily tolerated by the participant, causing minimal discomfort and not interfering with everyday activities.
Moderate event	An event that is sufficiently discomforting to interfere with normal everyday activities.
Severe event:	An event that prevents normal everyday activities.

12.3 Classification of relatedness

Not related	Temporal relationship of the onset of the event, relative to administration of the intervention, is not reasonable or another cause can by itself explain the occurrence of the event.

Unlikely to be related	Temporal relationship of the onset of the event, relative to administration of the intervention, is unlikely and it is likely there is another cause which can by itself explain the occurrence of the event.
Possibly related	Temporal relationship of the onset of the event, relative to administration of the intervention, is reasonable but the event could have been due to another, equally likely cause.
Probably related	Temporal relationship of the onset of the event, relative to administration of the intervention, is reasonable and the event is more likely explained by the intervention than any other cause.
Definitely related	Temporal relationship of the onset of the event, relative to administration of the intervention, is reasonable and there is no other cause to explain the event, or a re-challenge (if feasible) is positive.

12.4 Identification of AEs

In this study population (S)AEs are expected due to the participants being older residents in a care home setting. Care home staff are responsible for recording AEs for their residents, in the care home records as per standard practice. It is anticipated that most AEs related to infection and falls will be captured as part of the study CRF, but other AEs will not be recorded in the study documentation unless required for outcome measures.

12.5 Operational definitions for (S)AEs due to residents health status

Expected SAEs relating to residents' health status include (but are not limited to);

- Infection complications such as pneumonia
- Falls and near falls using PROFANE (Prevention of Falls Network Europe consensus, see http://www.profane.eu.org/) definitions respectively for falls as ''an unexpected event in which the participants come to rest on the ground, floor, or lower level''
- Frailty syndromes such as delirium and constipation
- Prolonged hospitalisation due to complications from initial injury
- Prolonged hospitalisation due to social care needs
- Pressure sores due to immobility

Death is also an expected outcome in this population.

SAEs related to the health status of older people will **not be** recorded or reported in this study (unless collected as one of the outcomes measures). This study is classified as low risk (the risk to participants taking part is no higher than that of standard care).

12.6 Operational definition of SAEs related to research procedures

We do not expect there to be any SAEs related to a research procedure, except for an SAE possibly related to the intervention. The only expected SAE related to the intervention (use of an air filter) is a trip, fall or injury due to the air filtration unit being placed in a resident's bedroom or in a communal area. SAEs that are related and expected will be recorded on the SAE log.

If an SAE occurs that care home staff judge to be related to a research procedure and is unexpected, this will be recorded on the SAE log and reported to the sponsor on a sponsor approved SAE form with 24 hours of becoming aware of the event.

Reportable SAEs include those relating to care home staff, residents or visitors.

12.7 Recording and reporting SAEs related to the research procedure

All SAEs that are related to the research procedure/ intervention and unexpected must be reported on a sponsor approved SAE initial report form, this may be provided orally but a written *SAE/SAR Initial Report Form*

must be completed within 24 hours of staff becoming aware of the event. A record of all recordable SAEs must be kept in the site file.

SAEs that are assessed as being possibly, probably or definitely related to the intervention/research procedure and unexpected, from baseline data collection until completion of the last trial-related procedure will be subject to expedited reporting procedures.

For all reportable SAEs the following information (as a minimum) will be collected:

- Full details in medical terms and case description
- Event duration (start and end dates, if applicable)
- Action taken
- Outcome
- Expectedness criteria
- Seriousness criteria

Sites should scan and email the SAE form, with high importance, to the (i) Sponsor, (ii) AFRI-c central trial team (Trial Manager), and (iii) cc'd Professor Alastair Hay, Chief Investigator; see 'Key Trial Contacts' for contact details (pages 2-3).

(Please note: typical University (central trial team) staff working hours are Monday to Friday, 09:00-17:00 (subject to variation). In the event of University closure dates or limited availability, an out of office automatic response will notify the site of alternative contact details/arrangements).

The Sponsor and/or central trial team will confirm receipt and, if required, forward the completed form to REC within the reporting periods (see below).

Each SAE must be reported to the Sponsor separately and not combined on one SAE form.
Any change of condition or other follow-up information relating to a previously reported SAE should be documented on the separate *SAE/SAR Follow Up Report Form* provided by the central trial team.

Events will be followed up until the event has resolved or a final outcome has been reached.

Figure 4 Overview of safety reporting requirements for SAEs related to the research procedure/intervention

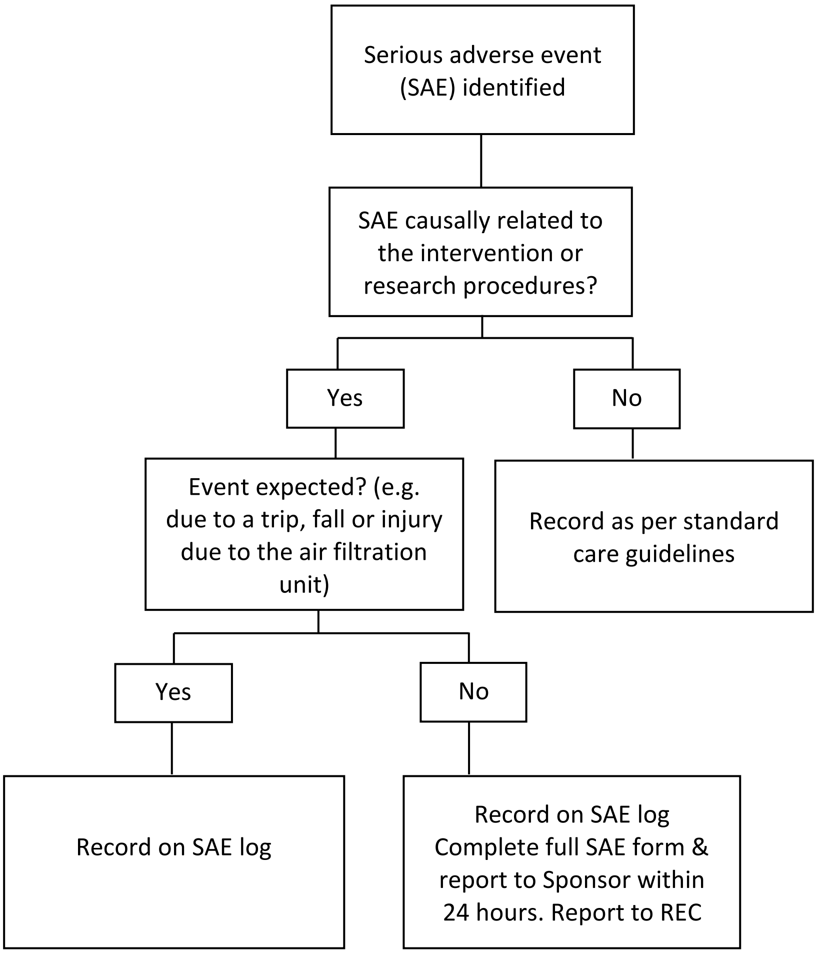

12.8 Responsibilities related to SAE reporting

Study champions – Study champions (or suitably trained delegates, including delivery teams) at each site be responsible for recording/reporting any SAEs that are related to research procedures this will include

- Using their professional judgement in assessing and assigning seriousness, causality and expectedness (where needed, the central study team will support this assessment).
- Ensuring that all appropriate S/AEs are documented.
- Ensuring that all SAEs are recorded and reported as per the procedures noted above, including the provision of further follow-up information as soon as available.
- Ensuring that SAEs are chased with the Sponsor/central trial team if a record of receipt is not received within 2-working days of initial reporting.

Study champions should also comply with any internal SAE reporting requirements within their care home.

Chief Investigator (CI) – The CI (or agreed delegate) will be responsible for:

- Clinical oversight of the safety of residents participating in the trial, including an ongoing review of the risk/benefit.
- Using medical judgement in assessing and assigning seriousness, causality and expectedness of SAEs where it has not been possible to obtain local medical assessment.
- Immediate review of SAEs requiring expedited reporting.
- Ensuring safety reports are prepared in collaboration with appropriate members of the TMG group for the relevant oversight committees and regulatory authorities.
- Expedited reporting of SAEs to the REC within required timelines.
- Notifying PIs of SAEs that occur within the trial.
- Central data collection of SAEs.

Trial Manager – The Trial Manager will provide a summary of S/AEs to the TSC and Sponsor as required.

12.9 Safeguarding procedures

Should residents or staff disclose information to suggest that they or others are at significant risk of harm, we may need to disclose these details to the designated safeguarding authority. A safeguarding protocol for researchers and participants will be implemented. See section 8.5 for details of the safeguarding procedure for residents during interviews.

13 STATISTICS AND HEALTH ECONOMICS ANALYSIS

13.1 Main trial

13.1.1 Sample size calculation

Previous research suggests ~30% of residents will wish to take part and ~40% will leave the care home or die during the study,(34) reflecting the frailty and vulnerability of the study population. To maintain study power, we will continually recruit new residents, resulting in an expected overall study data attrition of~20%. We consider the advantages in power outweighs the potential disadvantage arising from the unblinded recruitment of a proportion of study participants.

Data are surprisingly limited(45) regarding the incidence of infections in care homes. One widely cited study suggests healthy adults experience four to six respiratory per year.(46) A recent study found UK care home residents experienced an average of one respiratory infection per annum.(34) Other studies suggest the incidence of all medically diagnosed infections range from 3 to 9.8 per 1,000 resident care days, equivalent to between 1 and 3 infections per resident per year.(45) (5) Another study suggested the incidence of all infections per resident is one to three infections per annum,(47) with around 50% considered to be respiratory.(48)

Considering these together, and based on PPI and investigator experience, we expect care home residents to experience two respiratory infections during the winter months (usually 1 Sept to 31 May = 273 days). This will be monitored closely as one of our stop/go criteria (Table 2). Our PPI group advised that a reduction of one infection would be important to residents, relatives and staff.

Based on 90% power and an alpha of 0.05, to detect a reduction in winter respiratory infections of 1 per 242 person days from 2 to 1 per 242 person days, assuming a coefficient of variation of 0.78 to allow for between care home infection rate variation, calculated from data provided in personal communication from the PRINCESS study team,(34) a total of 74 care homes will be required. This assumes a mean cluster size of 12 for winter 3 (10 participants per care home for winters 1 & 2) and accounting for an attrition level as observed in PRINCESS,(34) and the continual recruitment strategy as described above, each cluster will provide a mean of 1920 person days of follow up. After reviewing accumulating data with the DMC and TSC on the number of follow up days, a decision was made to increase the number of care homes taking part in Winter 3 from 32 to 46 in order to achieve the number of person days required in the original calculation. This gives an approximate total of 91 care homes taking part in the study overall.

Each care home will participate for a single winter season (usually 1 Sept to 31 May). We estimate that 10 care homes will be randomised in winter 1 (the most experienced) and then 35 in winter 2 and 46 in Winter 3.

13.1.2 Data and statistical analysis

Analyses and reporting will be according to CONSORT guidelines(49) and will be pre-specified prior to the analysis in a detailed statistical analysis plan. Baseline data will be presented by trial arm, for both individual- and cluster-level characteristics, using descriptive statistics. Continuous measures will be presented using a measure of central tendency and variation as appropriate and categorical data as frequencies and percentages.

The primary effectiveness analysis will be conducted on the "consented" under the intention to treat principle (ITT) using mixed effects Poisson regression to estimate an incidence rate ratio (along with associated 95% confidence interval and p-value), comparing incidence of resident winter respiratory infection between groups. The primary analysis model will adjust for nursing care provision and deprivation tertile (stratification variables) and will include a random effect for care home to account for clustering. The use of a mixture distribution that captures the variation in rates across care-homes, and/or zero inflated models will be explored dependent on the nature of the data obtained. Robust standard errors will account for clustering if mixed effects models fail to converge.

The effect of the intervention on secondary outcomes in the ”consented” will be investigated using the same analytical approach as in the primary analysis; using mixed effects regression models that are appropriate to the nature of the outcome being analysed (e.g. a logistic model for a binary outcome).
Analyses will be repeated using anonymous data collected on all residents.

13.1.3 Health economic evaluation

A within-trial cost-consequence analysis (CCA) will be conducted, utilising primary and secondary outcome data, taking an NHS and care home provider perspective.

Intervention costs (total, incremental and mean per participant) will be based on the acquisition, set-up and installation (including care-home staff training), maintenance (e.g. filter replacement) and air filter running costs. The acquisition costs of the units are outlined in our treatment costs (see *Justification of Costs*). The typical lifecycle of the units will be sought from the manufacturer and used to annuitize the capital costs of running the units over subsequent winters. The total cost over the lifecycle will be calculated including the acquisition, installation and electricity running costs, the cost of the annual replacement filter and annual safety testing by an electrician. We will calculate the equivalent annual cost (discount rate 3.5% per annum). The lifecycle duration and further assumptions regarding equipment depreciation will be varied in a sensitivity analysis.

Downstream healthcare resource use will be collected via medical notes review where possible, or via care homes if necessary or via the study CRF. This includes (but is not limited to) data on GP (e.g. GP, practice nurse) and community (e.g. district nurse) contacts relating to respiratory infection episodes, ED visits, hospital admissions and antibiotic prescribing which will be valued using national sources of unit cost(50, 51) and/or market (actual) prices where applicable.

We acknowledge that the funding situation for air filters in care homes is complex, with the NHS, LA and private care home providers all being stakeholders. As part of the iterative development of a detailed health economic analysis plan, we will seek input from a range of funders with regards to the health economics data collection. We will meet with a range of potential funders (this may be virtually or in person, depending on attendee preference) in order to establish what metrics will be useful to support commissioning in their patch. While there is some uncertainty about who will ultimately pay for the intervention, we believe that the NHSand care home provider perspectives are most relevant, and that a cost-consequences analysis separated out by the different perspective will be useful to the range of potential funders.

Our scoping work has shown that care worker absence days due to illness and self-isolation are likely to result in significant productivity losses to care home providers. We will therefore consider the productivity costs relating to care home worker absence. Our care home provider collaborators will be used to validate the assumptions for costing these absences (e.g. the cost of backfill by an agency care worker may be appropriate). We acknowledge that there may be costs borne by care home residents and their families. These are not included within the economic evaluation. While we will explore these qualitatively we do not propose to add to respondent burden by asking for reporting.

The economic evaluation will correspond to the period in which air filters are implemented in care homes, i.e. one winter season usually runs from 1st September to 31st May (9 months). The analysis set for the primary economic evaluation will be the intention-to-treat population.

Additionally, we will provide economic data (including the installation cost of air filters which has been identified as relevant to stakeholders) and economic interpretation (for example examining the impact of spillover effects of air filters to non-participants) to support the scalability and dissemination plans (see section 18). We would proceed with our scalability work only if there is both evidence of effectiveness and potential evidence of cost-effectiveness.

14 DATA MANAGEMENT

14.1 Source data and documentation

When a resident is entered onto the study register, resident names will be entered on the study database for ease of data collection purposes for care home staff. Residents will have a unique participant identification number allocated to them and the resident name will be redacted from the central study team.

Data collected on residents will be anonymised to the study team unless an individual has consented to their personal data being shared with the study team. Data will be entered directly onto the password protected database and maintained on a SQL Server database system within the University of Bristol and will only be accessible to members of the research team. Any data stored on laptops will be encrypted. Any information that is analysed or transferred outside the EEA will be anonymised.

Information capable of identifying participants will not be removed from University of Bristol or care homes or made available in any form to those outside the trial, with the exception of NHS digital/UKHSA for linkage. Consent forms and clinical letters with personal identifiable data will be stored separately in a locked filing cabinet. Participant details will be anonymised in any publications that result from the trial.

Data collection will focus on outcomes as detailed in section 2 and Table 1.
Source data for this trial will by default consist of electronic versions of the care home expression of interest form, the resident register, care home baseline questionnaire, daily CRFs, SAE screening logs and participant/ staff completed questionnaires and other CRFs designed specifically for the study. Data will be entered onto the study specific database by delegated care home staff as soon as practical and where applicable or required, by the central research team / local delivery teams. Where electronic data collection is not possible, equivalent paper documents will become the source data. Paper records can be sent securely (electronically or by post) to the central trial team (study office) or local delivery teams for entry into the trial specific database.

Any paper documents containing identifiable information will be stored in a locked filing cabinet at site or central research offices, which only members of the local/central research team have access to. Local care homes must keep a paper record of consent form(s) in their site file for monitoring purposes, regardless of methods of data collection.

Other source data may include resident records/GP records and data routinely collected by UKHSA.

14.2 Storage and access to data

Data will be held at the University of Bristol and will conform to the University of Bristol Data Security Policy and in Compliance with the General Data Protection Regulation (GDPR) as it applies in the UK, tailored by the Data Protection Act 2018.

For monitoring purposes, the CI will allow monitors from the Sponsor (or delegate), persons responsible for the audit, representatives of the REC and other Regulatory Authorities to have direct access to source data/documents.

The Trial, and Data, Manager(s) (in collaboration with the CI) will manage access rights to the data set. Prospective new users must demonstrate compliance with legal, data protection and ethical guidelines before any data are released.

14.3 Archiving and destruction of trial materials

An archiving plan will be developed for all study materials. Data will be held in compliance with the Sponsor's SOPs. All research data will be retained in a secure location during the conduct of the trial and for at least 5 years after the end of the trial. Care home notes containing source data or other trial-related information should be identified by a label (or equivalent for electronic notes, where feasible) "Keep until at least dd/mm/yyyy" where the date given is at least five years (or applicable period) after the end of the trial. Data will be kept at the University of Bristol (and/or care homes) for this time and, at the end of the archiving

period, will be destroyed by confidential means with the exception of a final trial dataset which will be made available for data-sharing purposes (see section 14.4 below).

Where electronic records are in use, University of Bristol policy will be followed. The approval of University of Bristol as owner of the data and Study Sponsor, as well as the CI, will be sought prior to destruction of the data.

Participating care home sites will be responsible for ensuring that all study records held at site are archived appropriately when notified by the Sponsor/BTC (central trial team).

14.4 Access to the final study data set

Members of the TMG will develop a data sharing policy that is consistent with University of Bristol policy. We anticipate that anonymised trial data will be kept for future analysis and may be shared with other researchers, including those outside of the UK, EU and EEA, to enable international prospective meta-analyses.

The final trial data set will be stored as restricted data on the data.bris research data repository. Data will be made available to approved bona fide researchers only, after their host institution has signed a data access agreement. Details of how to request access are available at the University of Bristol's data repository website.

15 TRIAL MANAGEMENT

The CI will take overall responsibility for managing the various components of the trial, with the support of the Trial Manager(s), and will meet regularly (as required) with the leads for each component. The BTC, a UK Clinical Research Collaboration (UKCRC) registered trials unit, will support the delivery and conduct of the trial.

15.1 Trial management group (TMG)

The Trial Management Group (TMG), led by an experienced CI (Professor Hay), will comprise all investigators, the PPI representatives, the trial manager, the study nurse, and administrative staff. The TMG will be responsible for trial design, conduct, management, strategy, costs, data analyses and publication. With the support of all staff, the trial manager, will operationalise TMG strategy and oversee day to day management. The TMG will meet monthly to review progress against project specific milestones. Most meetings will be by video conference, but biannually face to face (when possible).

15.2 Trial Steering committee (TSC)

A Trial Steering Committee (TSC) will provide independent supervision of the trial on behalf of the Sponsor and NIHR. The TSC will focus on recruitment, adherence to the protocol, resident safety and consider new evidence. It will be chaired by an experienced public health/ geriatrician academic and independent members will include a clinical trialist, a statistician and PPI representation. Representation will be invited from the Host, Sponsor and the NIHR.

The TSC will comprise of an independent chair, plus at least three others. The independent members will cover expertise in (at least) statistics, trials and primary care. Care home stakeholders and PPI representatives may also be invited. Observers may also attend (including other members of the TMG or members of other professional bodies) at the invitation of the Chair. The TSC will meet for the first time prior to recruitment of the first participant and then at agreed intervals thereafter.

15.3 Independent Data monitoring committee (IDMC)

The Independent Data Monitoring Committee (IDMC) will meet shortly before each TSC to advise and make recommendations to the TSC regarding trial safety issues, or other reasons for the trial not to continue. If necessary, the IDMC will have access to unblinded trial data. Professor Kerry Hood, an experienced care home trial statistician has agreed to chair the IDMC. The DMC will comprise of an independent chair, plus at least two others. The IDMC will meet once prior to recruitment of the first participant and convene prior to the TSC meeting to review the AE data and any other ethical aspects that arise and report to the TSC. Responsibilities and reporting mechanisms of the DMC will be formalised in a DMC charter.

15.4 Patient and public involvement (PPI)

The patient advisory group (PAG) comprising adults with an interest in residents in care homes has been set up to support the trial. Members of the advisory group will be involved throughout the study. This will involve PAG advisory group meetings, specific roles on the TMG, review of the protocol, participant information, consent and data collection forms and informing dissemination of the research findings to participants.

The PAG will be actively involved in the design and development of trial-specific resident information resources, follow-up questionnaires, topic guides for interviews and methods for enhancing recruitment and follow-up rates.

We will consult with the PAG when we analyse and interpret the data from this study. Findings will be presented in lay terms at a PAG meeting. We will then seek the group's interpretation of the findings as a guide to whether a definitive trial will be feasible. They will advise us on routes for dissemination to patient groups and the best format for this.

15.5 Collaborators

Ms Jane Borland, care home manager; Dr Thornton, Public Health Registrar and honorary Research Fellow; Dr Hammond senior research associate in infectious diseases epidemiology; Dr Muir, a PHE Consultant Clinical

Scientist in Virology; Dr Noel, a Clinical Scientist in Microbiology and Dr Woodall, a Daphne Jackson Fellow and Medical Microbiologist. Collaborators will be invited to TMG meetings.

15.6 Sponsor and funding

This trial will be sponsored by UoB. The sponsor will be responsible for overall oversight of the trial.

This study/project is funded by the NIHR Public Health Research (PHR) Programme NIHR129783. The views expressed are those of the author(s) and not necessarily those of the NIHR or the Department of Health and Social Care.

16 MONITORING, AUDIT AND INSPECTION

16.1 Monitoring

The trial will be monitored and audited in accordance with the Sponsor's policy, which is consistent with the UK Policy Framework for Health and Social Care Research. All trial related documents will be made available on request for monitoring and audit by the Sponsor, the relevant Research Ethics Committee (REC) and for inspection by other regulatory bodies.

A Trial Monitoring Plan will be developed by the Sponsors and agreed by the TMG and CI based on the trial risk assessment which may include on-site monitoring. This will be dependent on a documented risk assessment of the trial.

The sponsor usually delegates some of the monitoring to the central research team. The following checks would be typical:

- That consent is taken by an appropriately authorised person
- That informed consent has been properly documented
- That data collected are consistent with adherence to the trial protocol
- That CRFs are only being completed by authorised persons
- That SAE recording and reporting procedures are being followed correctly
- That no key data are missing
- That data are valid
- Review of recruitment rates, changes in participation status and losses to follow-up.

On a regular basis we will monitor the percentage of residents that meet the eligibility criteria and report the percentage of participants who consent. To assess the generalisability of the participants, the available characteristics of eligible and non-eligible residents will be compared. We will also report to the DMC if requested, preliminary data on event rates observed in the trial population: including SAE rates and dropout and death rates.

16.2 Protocol compliance

There will be no prospective, planned deviations or waivers to the protocol. Any protocol deviations will be documented and reported to the Trial Manager, CI and Sponsor immediately (see Key Trial Contact for contact details). Information about protocol deviations will also be included in routine reports to the DMC and TSC. Protocol breaches identified by the central research team will be reported to the relevant study champion and Sponsor as soon as possible. The Sponsor will determine the seriousness of the breach.

In the event of systematic protocol deviations, investigation and remedial action will be taken in liaison with the CI, DMC and the TMG.

16.3 Notification of serious breaches to GCP and/or the protocol

A "serious breach" is a breach which is likely to effect to a significant degree:

- the safety or physical or mental integrity of the subjects of the trial; or
- the scientific value of the trial

The Sponsor will be notified immediately of any case where the above definition applies during trial conduct; they will determine the seriousness of the breach.

17 ETHICAL AND REGULATORY CONSIDERATIONS

17.1 Research governance

The study will be undertaken at multiple UK care homes, subject to appropriate REC approval and HRA approval. The trial will be conducted in accordance with the protocol, the conditions and principles of the Declaration of Helsinki and GCP. Any amendments of the protocol will be submitted to the REC for approval. On request, the study investigators and their institutions will permit trial-related monitoring and audits by the Sponsor and relevant REC by providing direct access to source data and other documents (i.e. residents' care home or medical notes, where relevant).

17.2 Governance and legislation

This trial will be conducted in accordance with:

- Conditions and principles of GCP guidelines
- Data Protection Act (DPA) 2018
- General Data Protection Regulation (GDPR)
- UK Policy Framework for Health and Social Care Research

Any amendments to the trial documents must be approved by the Sponsor prior to submission to the REC.

Before any site can enrol participants into the trial, the CI or designee will obtain confirmation of capacity and capability for each site in-line with HRA processes along with other documentation required for the sponsor to grant sites with a greenlight letter.

For all amendments the CI or designee will confirm with the Sponsor, the HRA (+/- REC) and sites that permissions are ongoing.

This research trial will be conducted in accordance with conditions and principles of GCP. GCP is the international ethical, scientific, recording and reporting studies that involve the participation of human subjects to which all clinical research is conducted. Compliance with this standard provides public assurance that the rights, safety and well-being of trial subjects are protected, consistent with the principles that originated in the Declaration of Helsinki and that the clinical trial data are credible.

17.3 Research Ethics Committee (REC) review and reports

Ethics review of the protocol for the trial and other trial related participant facing documents (e.g. RIS and consent forms) will be carried out by RECs. Any amendments to these documents, after a favourable opinion from the REC has been given, will be submitted to the REC for approval prior to implementation.

All correspondence with the REC will be retained in the Trial Master File (TMF)/ISF. An annual progress report will be submitted to the REC within 30-days of the anniversary date on which the favourable opinion was given, and annually until the trial is declared ended. The CI will notify the REC of the end of the trial and if the trial is ended prematurely (including the reasons for the premature termination). Within one year after the end of the trial, the CI will submit a final report with the results, including any publications/abstracts, to the REC.

ICH GCP training will be carried out by certain staff members depending on their delegated responsibilities within the trial, the level of training required will be determined according to the NIHR Delegation and Training Decision Aid. Informed consent to participate in the trial will be sought and obtained according to ICH GCP guidelines.

17.4 Peer review

The proposal for this trial has been peer-reviewed through the NIHR peer-review process, which includes independent expert and lay reviewers.

17.5 Poor quality data

The quality of the trial data will be monitored throughout the trial and data completeness will be reported to the TSC, and any cause for concern over data quality will be highlighted and an action plan put in place.

17.6 Financial and other competing interests

This applies to the CI and PIs at each site and committee members for the overall trial management. Research team, trial committee members and all PIs must disclose any ownership interests that may be related to products, services, or interventions considered for use in the trial or that may be significantly affected by the trial. Competing interests will be reported in all publications and in the final report.

17.7 Risks and benefits

We have conducted a full risk assessment and identified two areas consider medium risk (none were considered high risk). The first is timely procurement of the air filters. This will be mitigated by central monitoring by the University of Bristol Procurement Department, who is already working with Philips™ to ensure compliance with procurement law and contracting. The second is the impact of COVID-19, particularly for care homes already working at capacity. At the time of writing (December 2020) care home resident and staff vaccination has begun. It is not clear yet how long immunity will last, so the study has been designed to be conducted remotely, that is without study staff visiting care homes in person. We have surveyed care home managers to assess capacity to conduct study processes and been assured they will be possible. Once recruitment begins, the main threats to success have been included as internal pilot stop/go criteria.

The trial was designed anticipating the COVID-19 pandemic would remain active for at least the first study year. Since managers advised that research staff will not be welcome in care homes for the foreseeable future, it was concluded that a 'remote' data collection policy was required, with research staff supporting study activities with telephone and video communications.

The main risk of this approach is that staff will be too busy with care home responsibilities to assist with study activities. To minimise this, we will:

(i) Select care homes with the most research experience, via the NIHR ENRICH network and other routes
(ii) Consent and data collection will be overseen by a study researcher experienced and trained in the enrolment and retention of older people with and without capacity
(iii) Most data will be collected by care home staff (i.e. Not relying on residents to grapple with website forms) using simple-to-use forms collecting the minimum, necessary data
(iv) Appoint 'study champions' at each care home to act as study coordinators, points of contact and oversee study processes
(v) Providing financial reimbursement

As with all trials the main benefit of participating is an altruistic one to improve care and increase quality of living for care home residents.

The PIS will provide clear details of the anticipated risks and benefits of taking part in the study. The risk and benefits of the study will be discussed with the participating sites as part of the process of inviting residents to take part and providing written informed consent.

17.8 Indemnity

The necessary trial insurance is provided by the Sponsor. The PIS provides a statement regarding indemnity for negligent and non-negligent harm.

18 DISSEMINATION POLICY

The CI and TMG will establish a writing committee which will be responsible for preparing scientific reports of the study findings. The aim will be to publish a primary manuscript in a primary care medical journal, published as open access, with additional qualitative analyses described in specialty journals. Primary findings will also be presented at key meetings and conferences.

We will complete a full report for the NIHR. The results of this work are likely to influence the updates of the NICE guidelines for quality of life for care home residents.

With input from the University of Bristol Research Commercialisation Team, a business consultant will conduct a piece of market research with potential stakeholders including care home managers, CCG and Local Authority commissioners and national policy makers about priorities and mechanisms of funding and potential dissemination of study findings.

An engagement plan will be produced with the TMG, PAG and other collaborators. We will aim to disseminate the findings widely to the care home community, health care providers, policymakers and the public through presentations, clinical conferences and blogs. Furthermore, our team has experience of media contributions, including local, national and international television, radio and press contributions, as well as public lectures and writing for the public (e.g. the Conversation).

A study Twitter account and website will be set up. Various communication methods will be used to keep interested participants, managers and policy makers up-to-date with trial progress and results. These may include a study website, newsletters and social media, amongst others.

The Host will (NHS Bristol, North Somerset South Gloucestershire (BNSSG) CCG) will ensure NHS as well as public health engagement, with members of the CCG's Research Team supporting the project as well as the CCG's Clinical Lead for Integrated Care (a co-applicant). The CI holds an honorary contract with NHS BNSSG CCG, ensuring he benefits from academic and relevant NHS support.

19 SIGNATURE PAGE

The undersigned confirm that the following protocol has been agreed and accepted by the study Sponsor and by the REC and that the Chief Investigator agrees to conduct the trial in compliance with the approved protocol and will adhere to the principles outlined in UK Policy Framework for Health and Social Care Research, the Sponsor's SOPs, and other regulatory requirements as amended.

I agree to ensure that the confidential information contained in this document will not be used for any other purpose other than the evaluation or conduct of the clinical investigation without the prior written consent of the Sponsor.

I also confirm that I will make the findings of the study publicly available through publication or other dissemination tools without any unnecessary delay and that an honest accurate and transparent account of the study will be given; and that any discrepancies from the study as planned in this protocol will be explained.

Chief Investigator:
Signature:
.... Date: ....../......./.............
Name (please print):
....

Senior Statistician:
Signature:
.... Date: ....../......./.............
Name (please print):
....

20 REFERENCES

1. WHO. Coronavirus: World Health Organisation; 2020 [Available from: https://www.who.int/health-topics/coronavirus#tab=tab_1.
2. Morawska L, Cao J. Airborne transmission of SARS-CoV-2: The world should face the reality. Environment international. 2020;139:105730-.
3. WHO. WHO Coronavirus Disease (COVID-19) Dashboard: World Health Organisation; 2020 [Available from: https://covid19.who.int/.
4. Statistics OfN. Deaths involving COVID-19 in the care sector, England and Wales: deaths occurring up to 12 June 2020 and registered up to 20 June 2020 (provisional) https://www.ons.gov.uk/peoplepopulationandcommunity/birthsdeathsandmarriages/deaths/articles/deathsinvolvingcovid19inthecaresectorenglandandwales/deathsoccurringupto12june2020andregisteredupto20june2020provisional: Office for National Statistics; 2020 [
5. Büla CJ, Ghilardi G, Wietlisbach V, Petignat C, Francioli P. Infections and Functional Impairment in Nursing Home Residents: A Reciprocal Relationship. JAGS. 2004;52(5):700-6.
6. Muder RR, Brennen C, Swenson DL, Wagener M. Pneumonia in a Long-term Care Facility: A Prospective Study of Outcome. JAMA Internal Medicine. 1996;156(20):2365-70.
7. Spires SS, Talbot HK, Pope CA, Talbot TR. Paramyxovirus Outbreak in a Long-Term Care Facility: The Challenges of Implementing Infection Control Practices in a Congregate Setting. Infection Control & Hospital Epidemiology. 2017;38(4):399-404.
8. Statistics OfN. Overview of the UK population: July 2017. 2017.
9. Office for National Statistics. National Population Projections, 2012-based Statistical Bulletin. Office for National Statistics; 2013.
10. Parliament U. Care Standards Act. Her Majesty's Stationery Office; 2000.
11. Sundvall P-D, Stuart B, Davis M, Roderick P, Moore M. Antibiotic use in the care home setting: a retrospective cohort study analysing routine data. BMC geriatrics. 2015;15:71-.
12. Costelloe C, Metcalfe C, Lovering A, Mant D, Hay AD. Effect of antibiotic prescribing in primary care on antimicrobial resistance in individual patients: systematic review and meta-analysis. BMJ. 2010;340(May18_2):c2096.
13. Department of Health. Antimicrobial Resistance (AMR) Systems Map. 2014.
14. Kessel AS, Sharland M. The new UK antimicrobial resistance strategy and action plan. BMJ. 2013;346:f1601.
15. Public and International Health Directorate HPaERD, Infectious Diseases Branch 10200. Code of Practice on the prevention and control of infections and related guidance. 2015.
16. Agency DoHaHP. Prevention and control of infection in care homes – an information resource. 2013.
17. Jackson MM, Fierer J, Barrett-Connor E, Fraser D, Klauber MR, Hatch R, et al. Intensive Surveillance for Infections in a Three-year Study of Nursing Home Patients. American Journal of Epidemiology. 1992;135(6):685-96.
18. Mathur P. Hand hygiene: back to the basics of infection control. The Indian journal of medical research. 2011;134(5):611-20.
19. Fernstrom A, Goldblatt M. Aerobiology and Its Role in the Transmission of Infectious Diseases. Journal of Pathogens. 2013;2013:13.
20. Morawska L, Tang JW, Bahnfleth W, Bluyssen PM, Boerstra A, Buonanno G, et al. How can airborne transmission of COVID-19 indoors be minimised? Environment International. 2020;142:105832.
21. Bar-On YM, Flamholz A, Phillips R, Milo R. SARS-CoV-2 (COVID-19) by the numbers. eLife. 2020;9:e57309.
22. Kutter JS, Spronken MI, Fraaij PL, Fouchier RAM, Herfst S. Transmission routes of respiratory viruses among humans. Current Opinion in Virology. 2018;28:142-51.
23. Eames I, Tang JW, Li Y, Wilson P. Airborne transmission of disease in hospitals. Journal of the Royal Society, Interface. 2009;6 Suppl 6(Suppl 6):S697-S702.
24. Weinstein RA, Said MA, Perl TM, Sears CL. Gastrointestinal Flu: Norovirus in Health Care and Long-Term Care Facilities. Clinical Infectious Diseases. 2008;47(9):1202-8.
25. Mitchell B. Philips AC4600 H1N1 air purification certificate. Dublin; 2014.
26. Group S-EaM. Potential application of Air Cleaning devices and personal decontamination to manage transmission of COVID-19. 2020.

27. Hammond A, Khalid T, Thornton H, Woodhall C, Hay A. Should homes and workplaces purchase portable air filters to reduce the transmission of SARS-CoV-2 and other respiratory infections? A systematic review. PLoS ONE (accepted). 2020.
28. Vokurka S, Bystrická E, Svoboda T, Škoda Gorican IK, Sever M, Mazur E, et al. The availability of HEPA-filtered rooms and the incidence of pneumonia in patients after haematopoietic stem cell transplantation (HSCT): results from a prospective, multicentre, eastern European study. J Clin Nurs. 2014;23(11-12):1648-52.
29. Ethington T, Newsome S, Waugh J, Lee LD. Cleaning the air with ultraviolet germicidal irradiation lessened contact infections in a long-term acute care hospital. American Journal of Infection Control. 2018;46(5):482-6.
30. Little P, Stuart B, Hobbs FD, Moore M, Barnett J, Popoola D, et al. An internet-delivered handwashing intervention to modify influenza-like illness and respiratory infection transmission (PRIMIT): a primary care randomised trial. Lancet. 2015.
31. Sandora TJ, Taveras EM, Shih MC, Resnick EA, Lee GM, Ross-Degnan D, et al. A randomized, controlled trial of a multifaceted intervention including alcohol-based hand sanitizer and hand-hygiene education to reduce illness transmission in the home. Pediatrics. 2005;116(3):587-94.
32. Lee GM, Salomon JA, Friedman JF, Hibberd PL, Ross-Degnan D, Zasloff E, et al. Illness transmission in the home: a possible role for alcohol-based hand gels. Pediatrics. 2005;115(4):852-60.
33. Zazzara MB, Penfold RS, Roberts AL, Lee KA, Dooley H, Sudre CH, et al. Probable delirium is a presenting symptom of COVID-19 in frail, older adults: a cohort study of 322 hospitalised and 535 community-based older adults. Age Ageing. 2020.
34. Butler CC, Lau M, Gillespie D, Owen-Jones E, Lown M, Wootton M, et al. Effect of Probiotic Use on Antibiotic Administration Among Care Home Residents: A Randomized Clinical Trial. Jama. 2020;324(1):47-56.
35. Sekhon M, Cartwright M, Francis JJ. Acceptability of healthcare interventions: an overview of reviews and development of a theoretical framework. BMC Health Serv Res. 2017;17(1):88.
36. May C, Finch T. Implementing, Embedding, and Integrating Practices: An Outline of Normalization Process Theory. Sociology. 2009;43:535-54.
37. May CR, Mair FS, Dowrick CF, Finch TL. Process evaluation for complex interventions in primary care: understanding trials using the normalization process model. BMC Fam Pract. 2007;8:42.
38. Murray E, Treweek S, Pope C, MacFarlane A, Ballini L, Dowrick C, et al. Normalisation process theory: a framework for developing, evaluating and implementing complex interventions. BMC Med. 2010;8:63.
39. Ritchie J, Spencer L, O'Connor W. Qualitative research practice: A guide for social science students and researchers. London: Sage; 2003 2003.
40. Malterud K, Siersma VD, Guassora AD. Sample Size in Qualitative Interview Studies: Guided by Information Power. Qual Health Res. 2016;26(13):1753-60.
41. Guest G, Bunce A, Johnson L. How many interviews are enough? an experiment with data saturation and variability. Field Methods. 2006(15):59-82.
42. Braun V, Clarke V. Using thematic analysis in psychology. Qualitative Research in Psychology. 2006;3(2):77-101.
43. Braun V, Clarke V, Hayfield N, Terry G. Thematic Analysis. In: Liamputtong P. (eds), Handbook of research methods in health and social sciences Singapore: Springer; 2019. pp. 843-60 p.
44. Strauss A, Corbin J. Basics of Qualitative Research: Grounded Theory Procedures and Techniques: Sage, London; 1990.
45. Richards C. Infections in residents of long-term care facilities: an agenda for research. Report of an expert panel. J Am Geriatr Soc. 2002;50(3):570-6.
46. Worrall G. Common cold. Canadian family physician Medecin de famille canadien. 2011;57(11):1289-90.
47. Garibaldi RA. Residential care and the elderly: the burden of infection. J Hosp Infect. 1999;43 Suppl:S9-18.
48. Utsumi M, Makimoto K, Quroshi N, Ashida N. Types of infectious outbreaks and their impact in elderly care facilities: a review of the literature. Age and Ageing. 2010;39(3):299-305.
49. Campbell MJ. Extending CONSORT to include cluster trials. BMJ. 2004;328(7441):654-5.
50. Curtis L, Burns A. Unit Costs of Health and Social Care. Canterbury: Personal Social Services Research Unit, University of Kent; 2019.

51. NHS. National Cost Collection for the NHS England 2021 [Available from: https://www.england.nhs.uk/national-cost-collection/.

Document History

Version Number	Version Date	Summary of Changes
2.0	23 Sep 2021	Note – first version submitted to REC was v2.0
3.0	04 Nov 2021	In accordance with request from REC:<br>• Removed references to “nominated consultee”<br>• Updated references to “retrospective consent” in 7.2.3<br>• Removed intention to identify consultees before resident loses capacity<br>• Added information about resources available to support Care Homes in Section 6.3.4 (detailed information will be included in the contract with each site)<br>• Removed text about interviewing residents who decline participation from 6.5.2 and 8.3.1<br>• Updated section 6.10 to say that staff consenting residents will not be asked to complete staff questionnaires<br>• Clarified eligibility regarding CQC rating<br>Updated STOP/GO criteria (correction of typographical errors)<br>Corrected other typographical errors and updated contact details
4.0	08 Feb 2022	Updated the protocol to match the information sheets, consent form and A36 of the IRAS form to state that we would share identifiable information for consented residents with the UKHSA to obtain information about study outcome measures.<br>Also inserted the ISRCTN number and corrected 2 copy and paste errors relating to air filters in control group care homes (p27 and p33).
5.0	22 Feb 2022	We have made the following changes to the protocol:<br>1. Extended winter 1 to finish on 31 May (assuming covid-19 is still prevalent).<br>2. Changed the care home (CH) eligibility to enable us to include CHs with at least 20 residents (previously 30). We had based the original criteria on an expected consent rate of 30% (thereby obtaining our 10 consented residents per CH). However, we have been made aware that research active CHs are often smaller, we have had to turn away some slightly smaller CHs that wished to take part and early indications suggest that we may not need 30 residents to consent "the 10".<br>3. Updated the STOP/GO criteria, in discussion with the funder, to insert an additional criterion of "proportion of consented residents" completing the data collection.<br>4. Changed our resident inclusion criteria to include residents expected to reside in the CH for >=1 month (previously 2). Currently we can only

		replace consented residents who leave the study if we are at least 2 months before the end of the study. Relaxing this criterion will enable more residents to participate if any of "the ten" need to be replaced, which means we can collect more days of data for the study and makes it more likely that the internal pilot will meet the feasibility STOP/GO criteria.<br>5. Updated the process for remote consent to ensure it is feasible.<br>6. Updated the terminology of "Research nurse" to "Researcher" as research practitioners may be doing some of these tasks.<br>7. Clarified the PI may be a study champion, or the PI may delegate the role of "Study Champion" to other team member(s).<br>8. Added that we're asking for safeguarding policies to be provided for each care home (these will be filed so that we can refer to them if one of the AFRI-c researchers encounters an incident at the care home).<br>9. Minor amendments about the planned health economic stakeholder workshop (previously called focus group).<br>10. Correction of minor typographical errors.
6.0	4 April 2022	Updated information about where used filters will be sent and how filters would be disposed of following any analysis.<br>Corrected colours in the stop/go table
7.0	26 July 2022	• Updated the contact details, name and declaration for NIHR<br>• Updated the trial centre name (throughout) & contact details, and Sponsor contact details<br>• Capped the number of non-consented residents that care homes collect data on to approx 30 randomly selected residents<br>• Clarified that the GP notes review could take place later than May/June for winter 1 & 2<br>• Clarification of processes (e.g. identification / approach of sites, eligibility assessment, set-up, who asks the residents the questionnaires, how air filters are placed)<br>• Updated protocol (section 6.7) to be clear that "sufficient residents" may be <10 (at least 7 is expected to be the minimum number consented before data collection starts).<br>• Inserted text to allow some flexibility around what happens to the internal filters at the end of the winter period.<br>• Changed the timing of staff questionnaires at baseline to aim for completion before data collection starts (and therefore before air filters are switched on).

		• Updated protocol to allow for e-consent form to be used to document face to face consents where residents / consultees are unable to manage written consent.<br>• Inclusion of some qualitative interviews in the control care homes staff.<br>• Updated wording around communication methods to reflect current processes.<br>• Corrected some typographical errors and updated some wording to ensure meaning is clearer and correct throughout protocol.<br>• Included text to indicate additional support from CRN delivery teams (e.g. with SAE reporting).<br>• Deleted signature section for Sponsor.
8.0	25 Oct 2022	• Updated eligibility criteria for care homes to clarify that care home with capacity for at least 20 residents in single rooms would be eligible<br>• Introduced use of Nominated Consultees, independent from care home staff and the study. This is to enable residents without capacity or a willing and suitable relative or friend, to be included in the research.
8.0_updated	10 Nov 2022	• Removal of specific number of residents before data collection can start.
9.0	08 Mar 2023	• Changed “the ten” to reflect that there will be an average of 12 consented residents (maximum 16) per care home<br>• Increased the number of air filters supplied to care homes to a maximum of 21<br>• Updated end date of Winters 2 and 3 to 31st May
10.0	30 May 2023	• Total number of care homes increased to ~91 (10 in winter 1, 35 in winter 2, 46 in winter 3)<br>• Total number of consented residents increased to ~1002<br>• New secondary objective added for hospitalisations<br>• Details on residents with and without capacity to consent to take part updated for clarity and to reduce repetition of similar information in different sections<br>• Health economic initial stakeholder meeting changed to interviews
11.0	18 September 2023	• Due to supply issues, in Winter 3 up to 17 AC3033 devices will be used in private bedrooms in place of the AC2936/33 model. This change has been added to section 10.1 of the protocol
12.0	05 September 2024	• Changes made to section 13.1.3 – Health Economics evaluation – the first paragraph has been edited and now states that we will take an NHS and care home provider perspective when conducting a

		within-trial cost consequence analysis; Personal Social Service is now omitted.<br><br>In addition the following changes have been implemented to tables 1 and 3 and sections 2.3 and 6.12 because the study team are unable to count numbers of non-consented residents or staff members with infections in the UKHSA supplied HPZone outbreak data.<br>Secondary objectives –<br>• Removed the **all resident** population from four outcomes (Possible SARS-CoV-2 and Influenza-like illness infection episodes, PCR confirmed infections and other microbiologically confirmed infections).<br>• Removed the PCR confirmed infections and other microbiologically confirmed infections outcomes from the **staff members'** secondary outcomes.<br>• Added sickness days away from work due to respiratory infections and sickness days away from work due to any other infection to **staff members'** secondary outcomes.<br>• Added number of days experiencing PCR or other microbiologically confirmed infection outbreaks in **Care Homes** as an outcome.<br><br>• Clarified that the study team were not blinded to allocation (section 6.19)
