# Supplementary material for: High-Efficiency Particulate Air Filters to Prevent Winter Respiratory Infections in Care Homes: The AFRI-c Cluster Randomized Clinical Trial
Source: JAMA Intern Med. 2026 Jul 27:e262199. Online ahead of print. doi: 10.1001/jamainternmed.2026.2199 (PMC13409133; doi:10.1001/jamainternmed.2026.2199)
Supplement: Supplement 2. — eTable 1. Primary and secondary objectives and outcomes eMethods eTable 2. Description of residents’ intervention exposure eTable 3. Dates of key activities in participating care homes for AFRI-c eTable 4. Deviations from specified models eFigure 1. Communal room HEPA filter in-situ eFigure 2. Daily resident data collection for infection symptoms eFigure 3. Daily resident data collection confusion and falls/near falls eFigure 4. Duration of respiratory infection episodes by group eTable 5. Primary outcome pre-specified subgroup analyses: bedroom residents eTable 6. Secondary outcomes: bedroom residents eTable 7. Complier average causal effect (CACE) analyses: bedroom residents eTable 8. Baseline characteristics: communal room residents eTable 9. Secondary outcomes: communal room residents eTable 10. Complier average causal effect (CACE) analyses: communal room residents eTable 11. Secondary outcomes: staff absenteeism eTable 12. Secondary outcomes: care home microbiological outbreaks eTable 13. Description of turnover eTable 14. Primary and secondary outcomes presented per 1000 resident days: bedroom residents eReferences [file jamainternmed-e262199-s002.pdf]

Supplemental Online Content

Hay AD, Brierley RCM, Turner N, et al. High-efficiency particulate air filters to prevent winter respiratory infections in care homes: the AFRI-c cluster randomized clinical trial. *JAMA Intern Med*. Published online July 27, 2026. doi:10.1001/jamainternmed.2026.2199

eTable 1. Primary and secondary objectives and outcomes

eMethods

eTable 2. Description of residents' intervention exposure

eTable 3. Dates of key activities in participating care homes for AFRI-c

eTable 4. Deviations from specified models

eFigure 1. Communal room HEPA filter in-situ

eFigure 2. Daily resident data collection for infection symptoms

eFigure 3. Daily resident data collection confusion and falls/near falls

eFigure 4. Duration of respiratory infection episodes by group

eTable 5. Primary outcome pre-specified subgroup analyses: bedroom residents

eTable 6. Secondary outcomes: bedroom residents

eTable 7. Complier average causal effect (CACE) analyses: bedroom residents

eTable 8. Baseline characteristics: communal room residents

eTable 9. Secondary outcomes: communal room residents

eTable 10. Complier average causal effect (CACE) analyses: communal room residents

eTable 11. Secondary outcomes: staff absenteeism

eTable 12. Secondary outcomes: care home microbiological outbreaks

eTable 13. Description of turnover

eTable 14. Primary and secondary outcomes presented per 1000 resident days: bedroom residents

eReferences

This supplemental material has been provided by the authors to give readers additional information about their work.

© 2026 Hay AD et al. *JAMA Intern Med*.

eTable 1. Primary and secondary objectives and outcomes

Objective and outcome	Tool / method
**Primary: bedroom residents[a]**	
Symptomatic winter respiratory infection episodes	Resident symptoms measured daily by staff using paper/ electronic case report form (CRF/ eCRF)
**Secondary: bedroom residents[a]**	
a. Antibiotics prescribed (number and name)	Primary care medical notes review
b. Number of diagnosed respiratory (including COVID), gastrointestinal, skin and urinary infections	
c. Hospitalisations (all cause, and potentially air filter preventable)[b]	
d. Number of PCR confirmed SARS-CoV-2 infections	UKHSA Second Generation Surveillance System (SGSS)[e] data supplied by UKHSA
e. Number of PCR (or other test) confirmed Influenza A&B infections	
f. Number of other microbiologically confirmed infections as investigated by PHE during care home outbreaks, including Streptococcal, Meningococcal, Respiratory Syncytial Virus, Norovirus and Human Metapneumovirus infections	
**Secondary: communal room residents[c]**	
g. Number of days with respiratory infection symptoms	Daily CRF/eCRF
h. Presence of fever and/or delirium and/or acute deterioration in physical ability	
i. Number of gastro-intestinal infection episodes and number of symptomatic days of gastro-intestinal infection symptoms	
j. Number of days antibiotics are consumed	
k. Number of falls/near falls	
l. Number of possible[d] SARS-CoV-2 infection episodes and possible[d] Influenza-like illness episodes	
**Secondary: staff**	
m. Number of sickness days away from work	Care home manager CRF/eCRF weekly
n. Working days lost to sickness due to respiratory infections	
o. Working days lost to sickness due to any other infection	
**Secondary: care homes**	
p. Number of days experiencing PCR or other microbiologically confirmed infection outbreaks	HPZone[f] data supplied by UKHSA
**Secondary: intervention care homes only**	
q. Staff, bedroom resident and relatives/ friends' satisfaction with, and perception of, HEPAFUs	Questionnaires (to be reported in more detail elsewhere)
r. Fidelity to intervention	Staff weekly CRF/eCRF (to be reported in more detail elsewhere)

[a] Residents in intervention care homes exposed to HEPA filters in their private bedrooms and communal rooms (and their control group counterparts)

[b] Reasons for hospital admissions were reviewed to identify those potentially preventable by air filters, namely: respiratory tract infections (+/- sepsis); urinary tract infections (+/- sepsis); skin and soft tissue infections (+/- sepsis); sepsis (no focus identified); trauma (+/- fracture); and major adverse cardiovascular events (MACE). MACE was further categorized into: *arterial* (myocardial infarct, angina, new/exacerbation heart failure, transient ischaemic attack, haemorrhagic or thrombotic cerebrovascular accident) and *venous* (pulmonary embolism or deep vein thrombosis of the upper or lower limb).

[c] Residents in intervention care homes exposed to HEPA filters in communal rooms only (and their control group counterparts)

© 2026 Hay AD et al. *JAMA Intern Med.*

[d] Using UKHSA ‘possible’ case definitions: (i) COVID - new continuous cough OR temperature ≥37.8°C OR loss of, or change in, normal sense of smell or taste; and (ii) ILI - temperature ≥37.8°C AND one of the following: acute onset of at least one of the following respiratory symptoms (cough, with or without sputum), hoarseness, nasal discharge or congestion, shortness of breath, sore throat, wheezing, sneezing) OR an acute deterioration in physical or mental ability without other known cause.

[e] UKHSA Second Generation Surveillance System (SGSS) is the national laboratory reporting system used in England to capture routine laboratory data on infectious diseases and antimicrobial resistance. All diagnostic laboratories must notify the UKHSA following the identification of defined causative agents from human samples; this process is predominately through a daily feed to SGSS (see https://www.gov.uk/government/publications/sources-of-surveillance-data-for-influenza-covid-19-and-other-respiratory-viruses)

[f] UKHSA HPZone is a secure, web-based case and outbreak management system for use by UKHSA Health Protection Teams (HPT). Confirmed and suspected cases, clusters, environmental events and other intelligence notified to HPTs are usually but not exhaustively recorded on to HPZone.

© 2026 Hay AD et al. *JAMA Intern Med*.

Methods

Recruitment

Care homes: Some larger care homes reported having more than one self-contained household. In these circumstances care homes could opt to include some households in the study, providing that the household/s met the eligibility criteria.

Residents: Study information was shared with residents, relatives and staff, with opportunities provided to opt out of daily infection symptom data collection. Residents not opting out were added to a register from which they were randomly selected to participate. Where only some households within a care home were taking part in the study, only residents living within participating households were included on the resident register. In care homes with >30 residents, 30 residents were randomly selected by the database for anonymized daily infection symptom data collection. For care homes with ≤30 residents, all residents were included for anonymized daily infection symptom data collection. Within the group of 30 residents, the database also randomly selected residents be approached for consent. This process happened as soon as the care home had completed set-up, though if any residents declined to be part of the study (or left the study), the database randomly selected a 'replacement resident' to take their place. Once the required number of residents had been consented (or after 1 September, whichever was later), the care home was asked to initiate daily resident symptom data collection. Two days after this, intervention care home staff were asked to switch on, and leave on, all HEPA filters (eTable 3).

Intervention

Ideally care homes switched on the air filters on 1 September, though due to delays in set-up this was not always possible. They were also asked to record daily if they were switched on, in the correct location and using the correct setting, until 31 May each winter. If units were reported to the study team as being faulty, they were replaced. Both intervention and control group care homes were advised to continue following their usual infection prevention and control measures.

Data collection, primary outcome definition and sensitivity analyses

Care home staff reported daily symptom data using paper or electronic case report forms (CRFs, eFigures 2 and 3) between 1 September and 31 May.

As per previous studies,[1 2] the start of an infection episode was defined as the onset of two new (worsening) respiratory symptoms for at least one day, or one respiratory symptom for at least two days; and the end as the last symptomatic day preceding two asymptomatic days. When deriving episodes, valid days of daily data collection excluded days where a resident's data was not required, e.g. when hospitalized. Non-valid days did not contribute to episode start or end points. Days at risk was defined as valid days minus episode days including the two asymptomatic days that defined the end of an episode. If a 'communal room resident' was invited to replace a 'bedroom resident' who died or moved away, their contribution of symptomatic days was reallocated from the 'communal room resident' to the 'bedroom resident' dataset at the time they agreed.

To support sensitivity analyses and secondary outcomes, staff were also asked to report the presence of fever (defined as temperature ≥37.8°C); physical and/ or mental decline; subjective symptoms in residents able to report them (sore throat, earache and change in taste/smell) to facilitate sensitivity analyses, falls/near falls to assess safety, and the number of days antibiotics were consumed. Data quality and completeness were checked regularly by the research team, with queries followed until resolution.

© 2026 Hay AD et al. *JAMA Intern Med.*

For each staff sickness episode, one member of staff (an administrator or care home manager) documented on the study database: the staff role; start date; absence in hours; if agency staff were used to cover and; if the absence was due to infection (if yes: respiratory, urinary, skin, don't know, other - specify). This was considered reliable because the care homes kept central records for staff absence and the questions we asked were designed based on these data, so they could be completed retrospectively.

Serious adverse events

Based on the risk profile of the study, serious adverse events were only reported if related to study participation.

Sample size

The sample size assumed two respiratory infection episodes per winter (1 September to 30 April, 242 days) per bedroom resident (resident[b]), based on data from a previous trial.[3] Our PPI group advised that a reduction of one infection would be important to residents, relatives and staff. Based on 90% power and a 0.05 alpha, to detect a reduction from 2 to 1 infection per 242 resident[b] days, assuming a coefficient of variation of 0.78 to allow for variation between care homes in infection rates and 20% attrition, we calculated 74 care homes were required, with a mean cluster size of 10 residents[b] per care home.

Due to delays in starting recruitment at care homes in winters 1 and 2, we reviewed the number of resident[b] follow up days with the independent oversight committees during winter 2, and determined that we needed to increase the number of care homes (to 91), increase the number of residents[b] per care home (to 12 on average), and extend each winter data collection period (to 31 May). The trial was stopped at the end of May 2024, when we reached the required number of resident[b] follow up days.

Analytic methods

The statistical analysis plan SAP was finalized and agreed by the independent Trial Steering Committee (TSC), before data lock and any analyses performed. The primary effectiveness analysis and all count outcomes were conducted under the intention-to-treat principle using a mixed effects generalized linear model (Poisson with log link or negative binomial if an over-dispersed Poisson distribution was indicated) to estimate an incidence rate ratio (along with associated 95% confidence interval and p-value), comparing incidence of the outcome between groups. Days at risk were included as exposure in all models.

All models were adjusted for factors included in the randomisation stratification where possible. These factors were care home nursing care provision, socio-economic status (care home IMD tertile), and winter as fixed effects; and care home as a random effect to account for clustering. Cluster level outbreak outcomes were also adjusted for care home size using number of staff and number of residents at baseline. Where mixed effect models failed to converge then robust standard errors were used to account for clustering and / or covariates were excluded as appropriate. All count models used a negative binomial distribution, included care home as a random effect, and adjusted for all factors included in the randomisation stratification as specified in the SAP, except for those listed in eTable 4. For information regarding sub-group and compliance analyses, see Tables S5 and S7. Interaction terms were added to the primary analysis model to explore potential treatment effect heterogeneity by pre-specified subgroups (care home provides nursing care, frailty score, frequency of use of communal rooms, resident in receipt of nursing care, resident has dementia, resident has chronic lung disease, resident taking immunosuppressant drug(s)); and sensitivity analyses were performed on the

© 2026 Hay AD et al. *JAMA Intern Med.*

primary outcome (including alternative definitions of the primary outcome and exploring the impact of (through removal of) potential outliers). Level of intervention compliance is reported descriptively, and impact of intervention adherence was also explored as a sensitivity (CACE) analysis on both the primary resident[b] outcome and the associated secondary outcome in communal room residents.

Results are presented as treatment effects with 95% confidence intervals. Analyses were performed using Stata, version 18.0 (StataCorp, College Station, Texas) and SAS, version 9.4 1M5 (SAS Institute Inc., Cary, North Carolina).

Patient and public involvement

Care home staff and residents made recommendations regarding HEPA filter selection and air flow setting, and the clinically important effect size. Our two PPI co-applicants attended Trial Management Group meetings throughout the trial and advised on development of participant facing documents and dissemination materials. Both PPI members commented on the manuscript and one (JS) is an author. We had two PPI members on the TSC.

Amendments to protocol and statistical analysis plan

Pre-winter 2: In February 2022 we: extended the end of the winter period from 1 September to 30 April (=242 days in non-leap years) to 1 September to 31 May, because we identified that post-pandemic respiratory infections were persisting into April and May, and to enable the study to collect sufficient data for sample size requirements (NB. we continued to report outcomes per resident per winter where winter =242 days); changed the inclusion criteria at to include care homes with capacity for ≥20 residents in single rooms (previously ≥30 single rooms) due to being advised by the RDN network that most care homes interested in research were smaller care homes and observing that we should be able to meet the minimum number of residents in smaller care homes; changed our resident inclusion criteria to include residents expected to reside in the CH for ≥1 month (previously ≥2 months).

In July 2022 we capped the number of communal room residents in whom infection outcomes were being collected to 30 randomly selected residents (previously all residents) to enable very large care homes to take part; changed the timing of the baseline staff questionnaires to encourage completion before the start of data collection; made some minor changes to the consent process to ensure that it could be easily used, e.g. allowing e-consent forms to be used for face to face consents where residents / consultees were unable to complete written consent.

Pre-winter 3: In October 2022 we introduced the use of nominated consultees after a care home in winter 2 had been unable to participate due to none of their residents having capacity to consent, nor personal consultees. In March 2023 we increased the mean cluster size from 10 to 12 (max 16) and confirmed that winters 2 and 3 would also finish on 31st May (rather than 30th April), this was during winter 2 so some winter 2 care homes opted to only collect data until 30th April, as originally agreed.

In May 2023 we increased the number of care homes to 91 to enable enough daily days of data to be collected for sufficient power to confidently answer the research question; we added a new secondary objective to look at hospitalisations (all cause and potentially air filter preventable); amended the health economics stakeholder meeting to interviews. We also decided that as well as collecting all cause hospitalisation, we would review them to identify those potentially preventable by HEPA filers, namely: respiratory tract infections (+/- sepsis); urinary tract infections (+/- sepsis); skin and soft tissue infections (+/- sepsis); sepsis (no focus identified); trauma (+/- fracture); and major adverse cardiovascular events (MACE). MACE were further categorized into: arterial (myocardial infarct, angina, new/exacerbation heart failure, transient ischaemic attack, haemorrhagic or thrombotic

© 2026 Hay AD et al. *JAMA Intern Med.*

cerebrovascular accident) and venous (pulmonary embolism or deep vein thrombosis of the upper or lower limb).

During winter 3: In September 2023 we allowed for use of AC3033 devices to be used in bedrooms as a result of supply issues with the AC2939/33 model, we did not need to use AC3033 devices in any of the bedrooms for the study.

During close-down: In September 2024 we removed personal social service from the within-trial cost consequence analysis; we changed some secondary outcomes relating to UK Health Security Agency (UKHSA) data, due to the available dataset. Data were unavailable for UKHSA confirmed infections for “all resident” and “staff” populations, these were replaced with outcomes – ‘sickness days away from work due to respiratory infections’ and ‘sickness days away from work due to any other infection to staff members’ secondary outcomes’ and ‘number of days experiencing PCR or other microbiologically confirmed infection outbreaks in care homes’. These changes were made in the SAP before the final dataset was received from the UKHSA.

eTable 2. Description of residents’ intervention exposure

	**Intervention**	**Control**
‘Bedroom residents’ contributing to primary analyses[a]	HEPA filters in private bedrooms + communal rooms	Usual care only
‘Residents’ contributing to secondary analyses[b]	Communal room HEPA filters	Usual care only

[a] Tables 3 and eTables 5, 6, 7 and 14

[b] eTables 9 and 10

eTable 3. Dates of key activities in participating care homes for AFRI-c

Winter[a]	Site ID[b]	Randomisation[c]	Allocation	Consent greenlight[d]	Data collection greenlight[e]	Filters on[f]	Data collection complete[g]
1	1	19/01/2022	Intervention	02/02/2022	09/02/2022	14/02/2022	01/06/2022
1	2	13/01/2022	Control	03/02/2022	22/03/2022	.	01/06/2022
1	3	16/02/2022	Control	22/02/2022	08/03/2022	.	01/06/2022
1	4	24/01/2022	Control	03/02/2022	10/03/2022	.	01/06/2022
1	5	01/02/2022	Control	01/02/2022	17/03/2022	.	01/06/2022
1	6	08/02/2022	Intervention	17/02/2022	10/03/2022	12/03/2022	01/06/2022
1	7	21/02/2022	Intervention	22/02/2022	08/03/2022	10/03/2022	01/06/2022
1	8	09/03/2022	Intervention	14/03/2022	06/04/2022	11/04/2022	01/06/2022
1	9	09/03/2022	Intervention	14/03/2022	01/04/2022	05/04/2022	01/06/2022
1	10	28/03/2022	Intervention	11/04/2022	27/04/2022	29/04/2022	01/06/2022
2	11	08/08/2022	Control	01/09/2022	19/10/2022	.	31/05/2023
2	12	09/08/2022	Intervention	01/09/2022	19/09/2022	27/09/2022	15/05/2023
2	13	09/08/2022	Control	31/08/2022	20/09/2022	.	31/05/2023
2	14	17/08/2022	Control	09/09/2022	28/10/2022	.	31/05/2023
2	15	09/08/2022	Control	07/09/2022	26/09/2022	.	31/05/2023
2	16	11/08/2022	Control	14/09/2022	03/11/2022	.	31/05/2023
2	17	12/08/2022	Control	05/09/2022	29/09/2022	.	31/05/2023
2	18	08/08/2022	Control	01/09/2022	06/10/2022	.	31/05/2023
2	19	18/08/2022	Control	06/10/2022	05/12/2022	.	31/05/2023
2	20	11/08/2022	Intervention	08/09/2022	10/10/2022	12/10/2022	31/05/2023
2	21	22/08/2022	Control	25/10/2022	07/11/2022	.	31/05/2023
2	22	23/08/2022	Intervention	31/10/2022	16/11/2022	18/11/2022	31/05/2023
2	23	22/08/2022	Intervention	14/09/2022	29/09/2022	03/10/2022	31/05/2023
2	24	02/09/2022	Intervention	04/11/2022	23/11/2022	29/11/2022	15/05/2023
2	25	02/09/2022	Intervention	15/09/2022	03/10/2022	04/10/2022	31/05/2023
2	26	06/09/2022	Intervention	06/10/2022	21/01/2023	24/01/2023	31/05/2023

© 2026 Hay AD et al. *JAMA Intern Med*.

2	27	02/09/2022	Control	11/10/2022	21/12/2022	.	31/05/2023
2	28	06/09/2022	Control	15/11/2022	13/12/2022	.	19/05/2023
2	29	07/09/2022	Intervention	04/10/2022	04/11/2022	09/11/2022	31/05/2023
2	30	07/09/2022	Intervention	06/10/2022	24/10/2022	26/10/2022	31/05/2023
2	31	21/09/2022	Control	31/10/2022	17/11/2022	.	15/05/2023
2	32	22/09/2022	Intervention	03/11/2022	09/11/2022	25/11/2022	15/05/2023
2	33	03/10/2022	Control	29/11/2022	16/12/2022	.	31/05/2023
2	34	03/10/2022	Intervention	28/10/2022	10/11/2022	11/11/2022	31/05/2023
2	35	03/10/2022	Control	12/12/2022	16/01/2023	.	31/05/2023
2	36	03/10/2022	Control	07/11/2022	24/11/2022	.	31/05/2023
2	37	03/10/2022	Control	29/11/2022	13/12/2022	.	31/05/2023
2	38	24/10/2022	Control	04/11/2022	14/11/2022	.	31/05/2023
2	39	03/11/2022	Intervention	07/12/2022	13/12/2022	15/12/2022	31/05/2023
2	40	03/11/2022	Control	07/12/2022	22/12/2022	.	31/05/2023
2	41	28/10/2022	Intervention	01/12/2022	09/12/2022	12/12/2022	31/05/2023
2	42	03/11/2022	Intervention	29/11/2022	19/12/2022	21/12/2022	31/05/2023
2	43	14/11/2022	Intervention	02/12/2022	21/12/2022	23/12/2022	31/05/2023
2	44	09/11/2022	Intervention	13/12/2022	25/01/2023	31/01/2023	31/05/2023
2	45	17/11/2022	Intervention	12/12/2022	11/01/2023	13/01/2023	15/05/2023
3	1	06/06/2023	Control	04/07/2023	01/09/2023	.	03/06/2024
3	2	30/05/2023	Intervention	11/07/2023	07/09/2023	18/09/2023	03/06/2024
3	3	27/06/2023	Intervention	14/07/2023	01/09/2023	04/09/2023	03/06/2024
3	4	19/06/2023	Control	27/07/2023	19/09/2023	.	03/06/2024
3	5	06/06/2023	Intervention	27/07/2023	01/09/2023	05/09/2023	03/06/2024
3	6	14/06/2023	Control	31/07/2023	01/09/2023	.	03/06/2024
3	7	04/07/2023	Control	27/07/2023	01/09/2023	.	03/06/2024
3	8	03/07/2023	Control	30/08/2023	19/09/2023	.	03/06/2024
3	9	26/04/2023	Control	03/08/2023	01/09/2023	.	03/06/2024
3	10	15/06/2023	Control	10/07/2023	08/09/2023	.	03/06/2024

© 2026 Hay AD et al. *JAMA Intern Med*.

3	11	30/06/2023	Intervention	04/08/2023	01/09/2023	04/09/2023	03/06/2024
3	12	29/06/2023	Control	26/07/2023	04/09/2023	.	03/06/2024
3	13	27/06/2023	Intervention	18/07/2023	01/09/2023	06/09/2023	03/06/2024
3	15	30/06/2023	Intervention	14/07/2023	01/09/2023	11/09/2023	03/06/2024
3	16	03/07/2023	Control	12/09/2023	04/10/2023	.	03/06/2024
3	17	02/05/2023	Control	20/07/2023	01/09/2023	.	03/06/2024
3	18	13/06/2023	Intervention	14/08/2023	12/09/2023	14/09/2023	03/06/2024
3	19	07/07/2023	Intervention	24/07/2023	01/09/2023	04/09/2023	03/06/2024
3	20	03/07/2023	Intervention	18/07/2023	01/09/2023	05/09/2023	03/06/2024
3	21	07/07/2023	Intervention	27/07/2023	04/09/2023	06/09/2023	03/06/2024
3	22	07/07/2023	Intervention	26/07/2023	01/09/2023	05/09/2023	03/06/2024
3	23	12/07/2023	Control	09/08/2023	17/10/2023	.	03/06/2024
3	24	18/05/2023	Control	23/08/2023	11/10/2023	.	03/06/2024
3	25	11/07/2023	Control	31/07/2023	02/10/2023	.	03/06/2024
3	26	11/07/2023	Control	21/08/2023	11/10/2023	.	03/06/2024
3	28	11/07/2023	Intervention	31/07/2023	01/09/2023	07/09/2023	03/06/2024
3	29	11/07/2023	Control	26/07/2023	04/09/2023	.	03/06/2024
3	30	21/07/2023	Control	25/09/2023	05/10/2023	.	03/06/2024
3	31	21/07/2023	Intervention	22/08/2023	02/10/2023	04/10/2023	03/06/2024
3	32	24/07/2023	Control	05/09/2023	29/09/2023	.	03/06/2024
3	33	21/07/2023	Intervention	08/08/2023	01/09/2023	05/09/2023	03/06/2024
3	34	25/07/2023	Control	05/09/2023	28/09/2023	.	03/06/2024
3	35	13/07/2023	Intervention	11/08/2023	01/09/2023	05/09/2023	03/06/2024
3	36	08/08/2023	Intervention	16/08/2023	04/09/2023	06/09/2023	03/06/2024
3	37	08/08/2023	Control	08/09/2023	14/11/2023	.	03/06/2024
3	38	27/07/2023	Intervention	30/08/2023	10/10/2023	16/10/2023	03/06/2024
3	40	16/08/2023	Intervention	26/09/2023	09/10/2023	12/10/2023	03/06/2024
3	41	02/08/2023	Control	25/08/2023	27/09/2023	.	03/06/2024
3	42	17/08/2023	Control	03/10/2023	24/10/2023	.	03/06/2024

© 2026 Hay AD et al. *JAMA Intern Med*.

3	43	21/08/2023	Intervention	05/09/2023	02/10/2023	06/10/2023	03/06/2024
3	44	16/08/2023	Control	03/10/2023	17/10/2023	.	03/06/2024
3	45	01/09/2023	Control	15/09/2023	09/10/2023	.	03/06/2024
3	46	15/09/2023	Control	25/09/2023	18/10/2023	.	03/06/2024
3	47	06/10/2023	Intervention	16/10/2023	30/10/2023	02/11/2023	03/06/2024
3	48	06/11/2023	Control	13/11/2023	18/12/2023	.	03/06/2024
3	49	01/02/2024	Intervention	21/02/2024	26/03/2024	10/04/2024	03/06/2024

[a] Which winter the care home participated in the study
[b] The care home ID number
[c] The date that the care home was randomized
[d] The date that the care home was given permission to start consenting activities
[e] The date that the care home started collecting study data
[f] The date the air filters were switched on in the care home (intervention care homes only)
[g] The date that the daily data collection forms were switched off in the database.

© 2026 Hay AD et al. *JAMA Intern Med*.

eTable 4. Deviations from specified models

**Outcome**	Population	Distribution	Covariates removed	Clustering
**Sensitivity analysis 1 – fever required as a symptom**	Consented	Negative binomial	Winter	Sandwich estimator clustered on care home
**Gastrointestinal infection episodes**	Consented	Poisson	None	Care home as random effect
**Gastrointestinal infection episodes**	Non-consented	Poisson	None	Care home as random effect
**Possible SARS-CoV-2 infection episodes**	Non-consented	Poisson	None	Care home as random effect
**Possible flu-like infection episodes**	Consented	Poisson	Winter 1 (Winter retained as a covariate with winters 2 and 3)	Care home as random effect
**Possible flu-like infection episodes**	Non-consented	Poisson	None	Care home as random effect
**Confirmed Influenza A&B infections**	Consented	Negative binomial	Winter	Sandwich estimator clustered on care home
**Other microbiologically confirmed infections**	Consented	Negative binomial	Winter	Sandwich estimator clustered on care home
**Sickness days away from work**	Staff	Negative binomial	None	Sandwich estimator clustered on care home
**Sickness days due to respiratory infection**	Staff	Negative binomial	None	Sandwich estimator clustered on care home

© 2026 Hay AD et al. *JAMA Intern Med*.

eFigure 1. Communal room HEPA filter in-situ

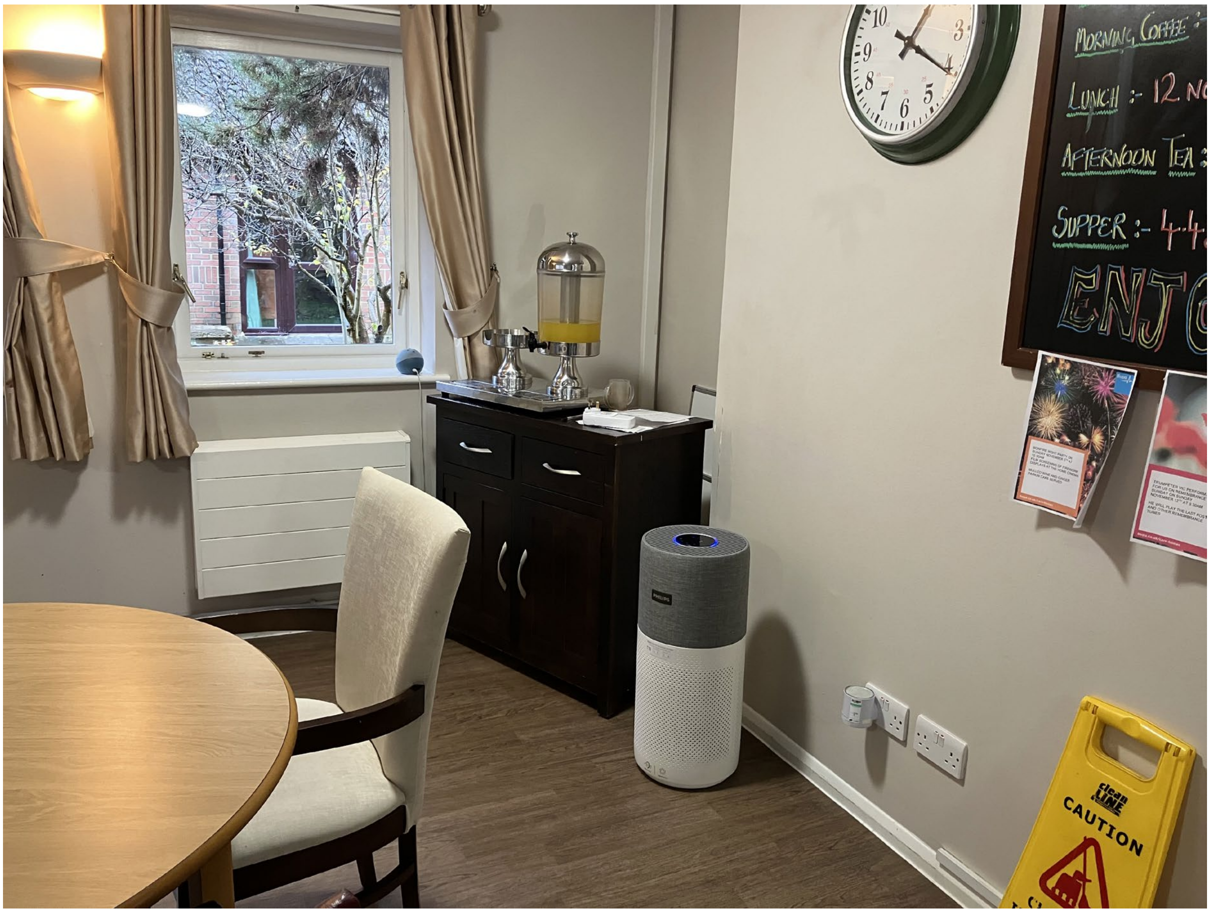

© 2026 Hay AD et al. *JAMA Intern Med*.

eFigure 2. Daily resident data collection for infection symptoms

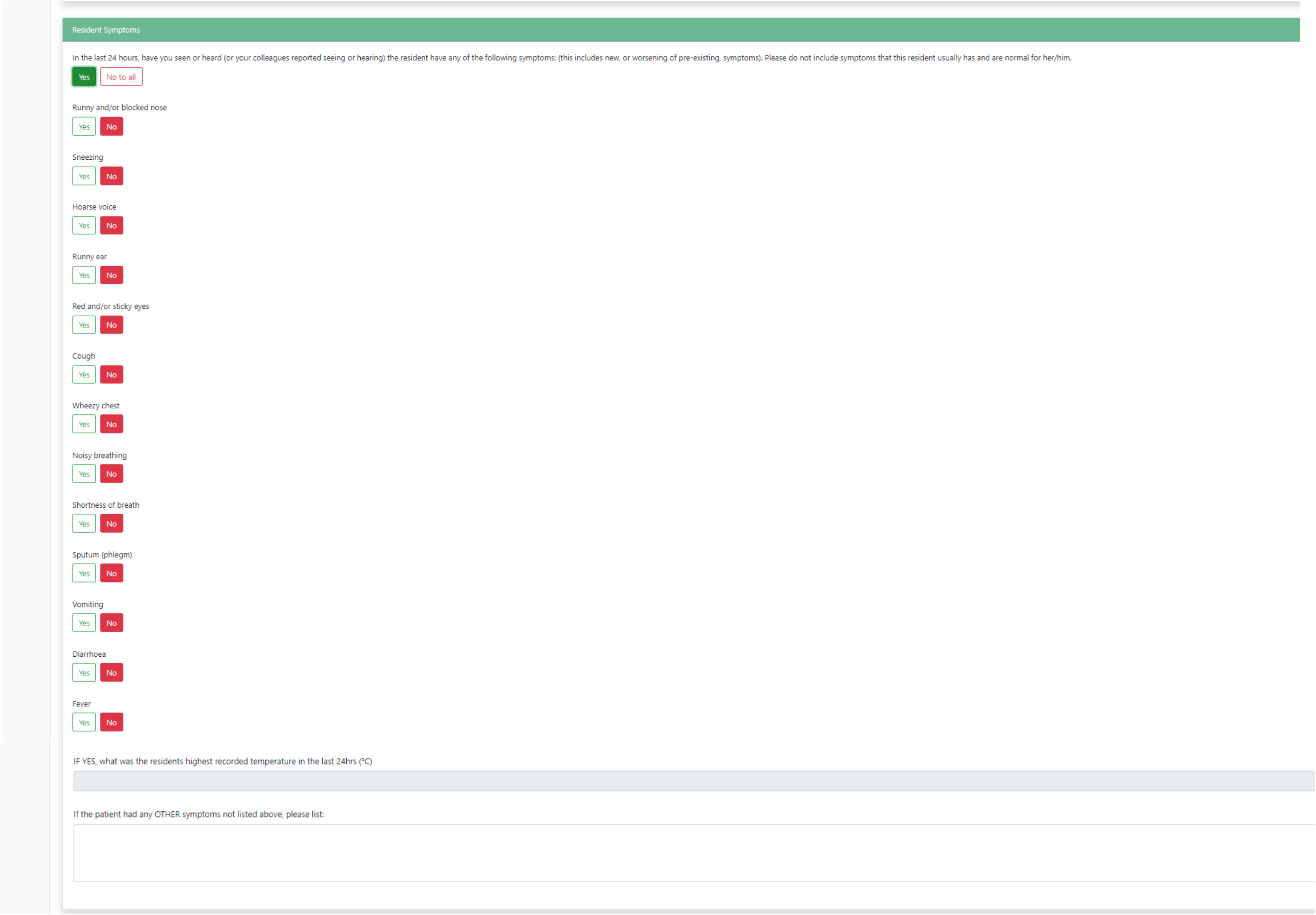

Resident Symptoms

In the last 24 hours, have you seen or heard (or your colleagues reported seeing or hearing) the resident have any of the following symptoms: (this includes new, or worsening of pre-existing, symptoms). Please do not include symptoms that this resident usually has and are normal for her/him.

Yes No to all

Runny and/or blocked nose

Yes No

Sneezing

Yes No

Hoarse voice

Yes No

Runny ear

Yes No

Red and/or sticky eyes

Yes No

Cough

Yes No

Wheezy chest

Yes No

Noisy breathing

Yes No

Shortness of breath

Yes No

Sputum (phlegm)

Yes No

Vomiting

Yes No

Diarrhoea

Yes No

Fever

Yes No

IF YES, what was the residents highest recorded temperature in the last 24hrs (°C)

If the patient had any OTHER symptoms not listed above, please list:

© 2026 Hay AD et al. *JAMA Intern Med*.

eFigure 3. Daily resident data collection confusion and falls/near falls

Resident wellbeing

Do you feel this resident has been more confused in the last 24 hours?

Yes No

Value is required

Do you feel this resident's physical ability has deteriorated in the last 24 hours?

Yes No

Antibiotics consumed in the past 24hrs? (Note to person completing from: By consumed, we mean any antibiotic prescribed by a healthcare professional that has been taken by mouth (liquid, tablet or capsule) or given directly into a vein (IV) or into a feeding tube. Please check with the BNF if you are not sure if a medication is an antibiotic.

Yes No

In the last 24 hours, has the resident had any falls? (Defined as "an unexpected event in which the participants come to rest on the ground, floor, or lower level.")

Yes No

If YES, how many?:

In the last 24 hours, has the resident had any near falls? (Defined as a stumble event or loss of balance that would result in a fall if sufficient recovery mechanisms were not activated)

Yes No

If YES, how many?

© 2026 Hay AD et al. *JAMA Intern Med*.

eFigure 4. Duration of respiratory infection episodes by group

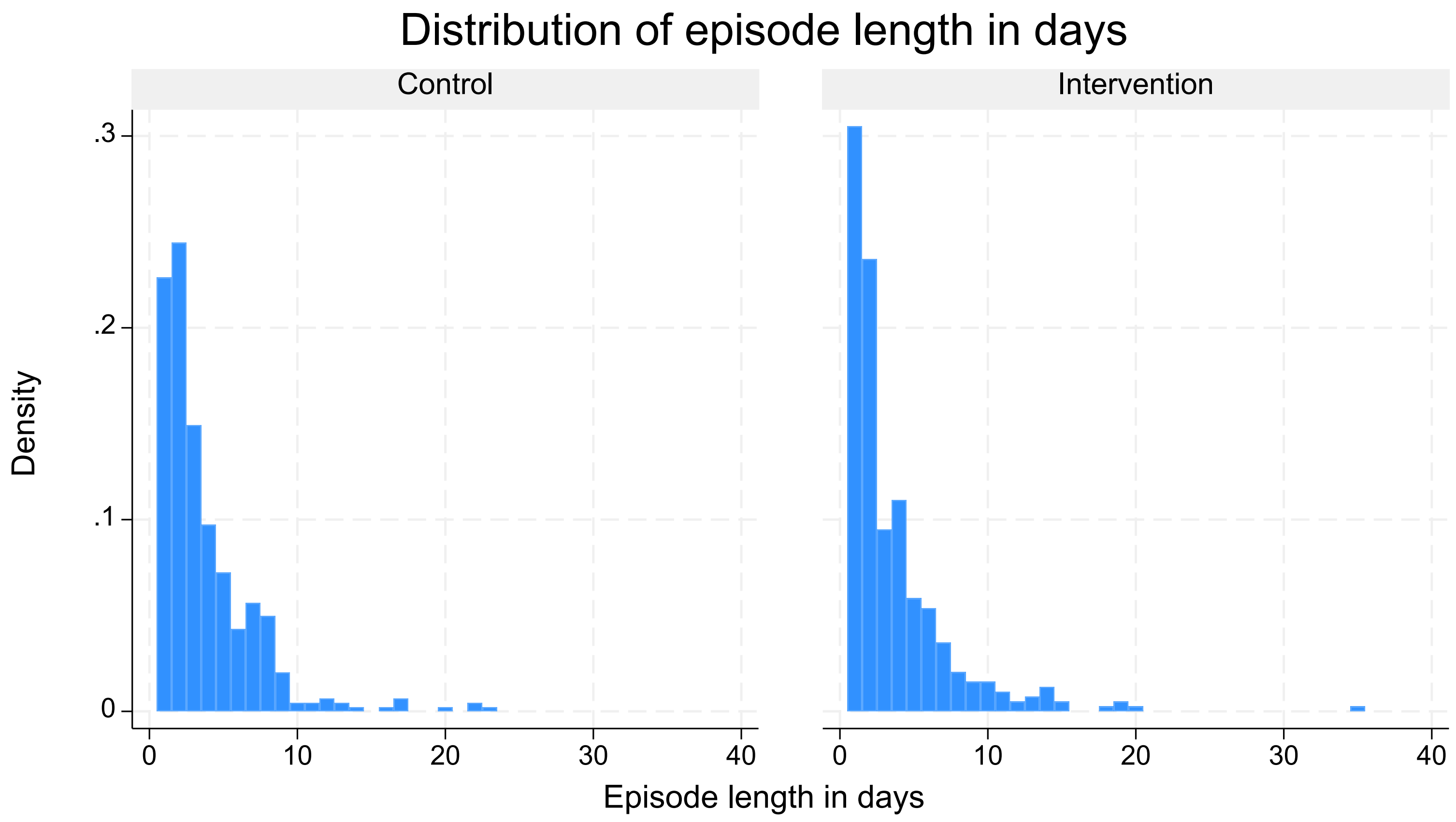

Overall median respiratory infection episode duration = 2 days, IQR: 1, 5; intervention group median respiratory infection episode duration = 2 days, IQR: 1, 5; control group median respiratory infection episode duration = 3 days, IQR: 2, 5.

© 2026 Hay AD et al. *JAMA Intern Med*.

eTable 5. Primary outcome pre-specified subgroup analyses: bedroom residents

Analyses	Intervention			Control			Allocation estimate			Subgroup interaction estimates		
	N[a]	Risk days[b]	Rate per resident per winter[c]	N[a]	Risk days[b]	Rate per resident per winter[c]	Adjusted IRR[d]	95% CI	p-value	Adjusted IRR[d] / χ2(d.f.)	95% CI	p-value
Care home provides nursing care	390	95235	0·99	442	102579	1·04	1·03	0·64, 1·65	0·90	0·77	0·37, 1·59	0·48
Rockwood frailty score (continuous)	390	95235	0·99	442	102579	1·04	0·64	0·27, 1·56	0·33	1·07	0·92, 1·25	0·38
Rockwood frailty score >5	390	95235	0·99	442	102579	1·04	0·86	0·51, 1·47	0·59	1·10	0·64, 1·88	0·74
Use of communal rooms[e]	354	80493	1·06	399	90850	1·06	1·11	0·64, 1·95	0·70	$X^2(2) = 2{\cdot}09$		0·35
Resident received nursing care[f]	390	95172	0·99	442	102579	1·04	0·95	0·62, 1·46	0·82	0·92	0·50, 1·71	0·80
Resident has dementia[f]	390	95235	0·99	442	102579	1·04	0·92	0·59, 1·44	0·72	1·00	0·63, 1·58	0·99
Resident has chronic lung disease[f]	347	87297	0·96	412	94930	1·05	0·89	0·60, 1·31	0·55	0·98	0·58, 1·65	0·93
Resident on immunosuppressing drugs[g]	349	87679	0·96	417	95526	1·06	0·86	0·59, 1·27	0·45	1·40	0·43, 4·57	0·58

[a] Number of resident respiratory infection episodes

[b] Number of 'risk' days defined as the number of valid days, minus the number of episode days, minus two days (that define episode end), plus one person day to ensure risk >0. Valid days defined as the resident follow-up days with daily data collection, excluding days when a resident's data is not required, or a resident is not in the care home, e.g. hospitalized

[c] Number of symptomatic winter respiratory infection episodes per 242-resident 'risk' days.

[d] Incident rate ratio, adjusted for nursing care provision, deprivation tertile, and winter

[e] Use of communal rooms – categorical variable with three levels: visits communal rooms less than 2 days per week, visits communal rooms 2-4 days per week, visits communal rooms more than 4 days per week

[f] Baseline measures

[g] Immunosuppressing drugs included: chemotherapy; radical radiotherapy; immunosuppressive or immunomodulating biological therapy including anti-TNF, alemtuzumab, ofatumumab, rituximab; patients receiving protein kinase inhibitors or PARP inhibitors; steroid sparing agents such as cyclophosphamide and mycophenolate mofetil; systemic steroids for >1 month at a dose equivalent to prednisolone at ≥20mg; long term immunosuppressive treatment for conditions including systemic lupus erythematosus, rheumatoid arthritis, inflammatory bowel disease, scleroderma and psoriasis.

© 2026 Hay AD et al. *JAMA Intern Med*.

eTable 6. Secondary outcomes: bedroom residents

Infection-related outcomes	Intervention			Control					
	N[a]	Resident -days[b]	Rate per resident per winter[c]	N[a]	Resident -days[b]	Rate per resident per winter[c]	Adjusted IRR[d]	95% CI	p-value
Days with respiratory infection symptoms[e]	1416	94666	3·60	1643	101990	3·88	0·77	0·46, 1·29	0·32
Episodes of fever and/or delirium and/or acute deterioration in physical ability[e]	281	96139	0·71	309	103679	0·72	0·85	0·57, 1·28	0·45
Days with fever and/or delirium and/or acute deterioration in physical ability[e]	774	96139	2·92	857	103679	2·98	0·80	0·43, 1·48	0·47
Episodes of gastro-intestinal infection[e]	61	97118	0·15	48	104877	0·11	1·28	0·53, 3·09	0·59
Days with gastro-intestinal infection symptoms[e]	167	97118	0·42	103	104877	0·24	1·22	0·33, 4·50	0·77
Antibiotic courses prescribed[f g]	572	89567	1·55	599	98322	1·47	1·14	0·86, 1·53	0·36
Days antibiotics taken[e]	2067	92924	5·38	2305	100298	5·56	0·69	0·43, 1·11	0·13
Number of falls / near-falls[e]	281	95349	0·71	422	102904	0·99	0·64	0·35, 1·17	0·15
Episodes of possible SARS-CoV-2-infection[e f]	191	96127	0·48	245	103563	0·57	0·81	0·55, 1·21	0·31
Episodes of possible influenza-like-illness[e g]	14	93099	0·04	23	102054	0·05	0·55	0·22, 1·3706	0·20
PCR confirmed SARS-CoV-2 infections[h j]	31	96834	0·08	19	104485	0·04	1·70	0·76, 3·78	0·19
PCR confirmed Influenza A&B infections[h j]	6	96834	0·02	7	104485	0·02	0·98	0·21, 4·52	0·97
Microbiologically confirmed care home infection outbreaks[h j k]	8	96834	0·02	8	104485	0·02	0·97	0·31, 3·04	0·96
GP diagnosed respiratory, gastrointestinal, skin and urinary infections[h i]	655	89567	1·77	640	98386	1·57	1·21	0·90, 1·62	0·20

© 2026 Hay AD et al. *JAMA Intern Med*.

Hospitalisations: all causes[h i]	322	89567	0·87	320	96309	0·80	1·15	0·75, 1·76	0·53
Hospitalisations: potentially air filter preventable[h i l]	93	89567	0·25	113	96309	0·28	0·89	0·56, 1·42	0·63
**Residents' perception of care home[m]**	**N[n]**			**N[n]**			**OR[o]**	**95% CI**	**p-value**
Temperature	356	--	--	393	--	--	1·00	0·62, 1·61	0·99
Odour	354	--	--	393	--	--	0·77	0·47, 1·24	0·22
Air quality	356	--	--	393	--	--	0·71	0·47, 1·08	0·11

[a] Number of outcome events

[b] Number of follow-up days

[c] Number of symptomatic winter respiratory infection episodes per 242-resident days

[d] Incident rate ratio, adjusted for nursing care provision, deprivation tertile, and winter

[e] Rate determined using 'risk' days, defined as the number of valid days, minus the number of episode days, minus two days (that define episode end), plus one person day to ensure risk >0.

[f] Using UKHSA 'possible' COVID case definition: new continuous cough OR temperature ≥37.8°C OR loss of, or change in, normal sense of smell or taste

[g] Using UKHSA 'possible' influenza-like-illness case definition: temperature ≥37.8°C AND one of: acute onset of at least one of the following respiratory symptoms: cough (with or without sputum), hoarseness, nasal discharge or congestion, shortness of breath, sore throat, wheezing, sneezing) OR an acute deterioration in physical or mental ability without other known cause

[h] Rate determined using 'valid' days defined as the resident follow-up days with daily data collection, excluding days when a resident's data is not required, or a resident is not in the care home, e.g. hospitalized

[i] Data collected from GP medical records

[j] Data provided by UKHSA

[k] Streptococcal, Meningococcal, Respiratory Syncytial Virus, Norovirus and Human Metapneumovirus infections

[l] Defined as respiratory tract infections (+/- sepsis); urinary tract infections (+/- sepsis); skin and soft tissue infections (+/- sepsis); sepsis (no focus identified); trauma (+/- fracture); and major adverse cardiovascular events (MACE). MACE will be further categorized into: arterial (myocardial infarct, angina, new/exacerbation heart failure, transient ischaemic attack, haemorrhagic or thrombotic cerebrovascular accident) and venous (pulmonary embolism or deep vein thrombosis of the upper or lower limb)

[m] Collected using follow-up questionnaires

[n] Number of residents

[o] Odds ratio, adjusted for nursing care provision, deprivation tertile, and winter

© 2026 Hay AD et al. *JAMA Intern Med*.

eTable 7. Complier average causal effect (CACE) analyses: bedroom residents

	Intervention			Control					
	N[a]	Resident days[b]	Rate per resident per winter[c]	N[a]	Resident days[b]	Rate per resident per winter[c]	Adjusted IRR[d]	95% CI	p-value
Potential outliers removed[e]	370	93881	0.95	393	99619	0.95	0.99	0.71, 1.39	0.98
CACE: binary compliance	390	95235	0.99	442	102579	1.04	1.09	0.61, 1.95	0.77
CACE: continuous compliance	390	95235	0.99	442	102579	1.04	1.10	0.58, 2.09	0.77
Missing data sensitivity analysis 1[f]	390	95235	0.99	1078	98870	2.64	0.33	0.23, 0.47	<0.001
Missing data sensitivity analysis 2[g]	1534	90261	4.11	442	102579	1.04	3.66	2.47, 5.41	<0.001

[a] Number of outcome events

[b] Number of days at risk. Days at risk is defined as the number of valid days, minus the number of episode days, minus the number of 'dead days' (the two days immediately following an episode, plus one person day to ensure risk >0.

[c] Number of symptomatic winter respiratory infection episodes per 242-person risk days.

[d] Estimated incident rate ratio, adjusted for nursing care provision, deprivation tertile, and winter

[e] Outlier care homes, as assessed by Tukey's fences, removed from analysis

[f] Missing runs of days in non-symptomatic periods are assessed using a best / worst case scenario, where control is worst (episode) and intervention is best (no episode)

[g] Missing runs of days in non-symptomatic periods are assessed using a best / worst case scenario, where control is best (no episode) and intervention is worst (episode)

© 2026 Hay AD et al. *JAMA Intern Med*.

eTable 8. Baseline characteristics: communal room residents[a]

	Intervention<br>N = 1004	Control<br>N = 932	Overall<br>N = 1936
Winter randomized			
1	157 / 1004 (15·6%)	73 / 932 (7·8%)	230 / 1936 (11·9%)
2	370 / 1004 (36·9%)	335 / 932 (85·5%)	705 / 1936 (36·4%)
3	477 / 1004 (47·5%)	524 / 932 (56·2%)	1001 / 1936 (51·7%)
Age[b]	87 (81, 92)	87 (81, 92)	87 (81, 92)
Has dementia	670 / 1004 (66·7%)	567 / 932 (60·8%)	1237 / 1936 (63·9%)
Frailty score	6 (4, 7)	6 (5, 7)	6 (4, 7)
Has received Influenza vaccine	939 / 1004 (93·5%)	869 / 932 (93·2%)	1808 / 1936 (93·4%)
Has received COVID-19 vaccine	964 / 1004 (96·0%)	876 / 931 (94·1%)	1840 / 1935 (95·1%)

[a] Data are medians (IQR)/Mean (SD) or n / N (%). Difference between denominators in n / N (%) figures and overall N are the numbers of missing values for the respective measurement. Unless otherwise stated, all data collected at resident screening (consent). Number of residents includes all anonymous residents over the winter periods who had valid daily data collection.
[b] Missing data (Intervention, control): 1 (1, 0).

© 2026 Hay AD et al. *JAMA Intern Med*.

eTable 9. Secondary outcomes: communal room residents

Secondary outcome	Intervention			Control					
	N[a]	Resident-days[b]	Rate per resident per winter[c]	N[a]	Resident-days[b]	Rate per resident per winter[c]	Adjusted IRR[d]	95% CI	p-value
Respiratory infection episodes[e]	483	126067	0·93	511	128641	0·96	0·91	0·67, 1·23	0·54
Respiratory infection symptomatic days[e]	1822	125063	3·50	1820	127709	3·42	0·79	0·48, 1·30	0·36
Fever and/or delirium and/or acute deterioration in physical ability episodes[e]	299	127397	0·57	343	129904	0·64	0·69	0·46, 1·06	0·09
Fever and/or delirium and/or acute deterioration in physical ability symptomatic days[e]	879	127397	1·66	929	129904	1·73	0·49	0·26, 0·90	0·02
Gastro-intestinal infection episodes[e]	48	128579	0·09	43	131232	0·08	1·02	0·42, 2·47	0·97
Gastro-intestinal infection symptomatic days[e]	118	128579	0·22	117	131232	0·22	1·14	0·30, 4·41	0·85
Days taking antibiotics[e]	1937	119587	3·92	2787	121457	5·55	0·57	0·37, 0·87	0·01
Falls / near-falls[e]	347	122723	0·68	429	125886	0·82	0·68	0·38, 1·22	0·20
Possible SARS-CoV-2-infection episodes[e f]	224	127293	0·43	255	129840	0·48	0·83	0·58, 1·19	0·32
Possible influenza-like episodes[e g]	17	119080	0·03	21	126358	0·04	0·60	0·21, 1·68	0·33

© 2026 Hay AD et al. *JAMA Intern Med*.

[a] Number of outcome events

[b] Number of follow-up risk days or valid days. For daily data related outcomes risk days are used. For GP and UKHSA related outcomes valid days are used. Valid days are defined as the resident follow-up days with daily data collection, excluding days when a resident's data is not required, or a resident is not in the care home, e.g., hospitalized, other medical appointment. Risk days are defined as number of valid days – episode days – dead days + 1 day per participant to avoid participants having 0 risk. Dead days are defined as the two days immediately following an episode.

[c] Number of symptomatic winter respiratory infection episodes per 242-resident days

[d] Incident rate ratio, adjusted for nursing care provision, deprivation tertile, and winter

[e] Derived from daily data collection where staff were asked to report new (or worsening of pre-existing) objective respiratory symptoms (e.g. runny/blocked nose; sneezing; cough; wheeze or sputum) reported daily by staff between 1 September and 31 May; infection episode start defined as the onset of two new (worsening) respiratory symptoms for at least one day, or one respiratory symptom for at least two days; and the end as the last symptomatic day preceding two asymptomatic days. Rate determined using 'risk' days, defined as the number of valid days, minus the number of episode days, minus two days (that define episode end), plus one person day to ensure risk >0.

[f] Using UKHSA 'possible' COVID case definition: new continuous cough OR temperature ≥37.8°C OR loss of, or change in, normal sense of smell or taste

[g] Using UKHSA 'possible' influenza-like-illness case definition: temperature ≥37.8°C AND one of: acute onset of at least one of the following respiratory symptoms: cough (with or without sputum), hoarseness, nasal discharge or congestion, shortness of breath, sore throat, wheezing, sneezing) OR an acute deterioration in physical or mental ability without other known cause

© 2026 Hay AD et al. *JAMA Intern Med*.

eTable 10. Complier average causal effect (CACE) analyses: communal room residents

	Intervention			Control					
	N[a]	Resident days[b]	Rate per resident per winter[c]	N[a]	Resident days[b]	Rate per resident per winter[c]	Adjusted IRR[d]	95% CI	p-value
CACE: binary compliance	483	126067	3.83	511	128641	3.97	0.82	0.47, 1.44	0.50
CACE: continuous compliance	483	126067	3.83	511	128641	3.97	0.80	0.43, 1.51	0.49

[a] Number of outcome events

[b] Number of days at risk. Days at risk is defined as the number of valid days, minus the number of episode days, minus the number of 'dead days' (the two days immediately following an episode, plus one person day to ensure risk >0.

[c] Number of symptomatic winter respiratory infection episodes per 242-person risk days.

[d] Estimated incident rate ratio, adjusted for nursing care provision, deprivation tertile, and winter

eTable 11. Secondary outcomes: staff absenteeism

	Intervention			Control					
	N[a]	Staff - days[b]	Days per FTE staff per winter[c]	N[a]	Staff-days[b]	Days per FTE staff per winter[c]	IRR[d]	95% CI	p-value
Working days lost to sickness	5153	247373	5·04	7339	308235	5·76	0·80	0·55, 1·16	0·24
Working days lost to sickness due to respiratory infections	1334	251192	1·29	1591	313983	1·23	1·03	0·63, 1·68	0·90
Working days lost to sickness due to any other infection	465	252061	0·57	745	314829	0·45	0·80	0·26, 2·50	0·70

[a] Number of outcome events

[b] Number of full-time equivalent (FTE) staff days based on full-time and part-time staff hours at baseline

[c] Number of symptomatic winter respiratory infection episodes per 242-FTE staff days

[d] Incident rate ratio, adjusted for nursing care provision, deprivation tertile, and winter

© 2026 Hay AD et al. *JAMA Intern Med*.

eTable 12. Secondary outcomes: care home microbiological outbreaks

	Intervention			Control					
	N[a]	Care home days[b]	Rate per winter[c]	N[a]	Care home days[b]	Rate per winter[c]	IRR[d]	95% CI	p-value
Number of PCR or other microbiologically confirmed infection outbreaks	19	8986	0·51	26	10031	0·63	0·78	0·41, 1·45	0·43
Number of days experiencing PCR or other microbiologically confirmed infection outbreaks	203	8783	5·59	249	9782	6·16	0·59	0·34, 1·00	0·05

[a] Number of outcome events

[b] Number of person days defined as expected FTE hours based on full-time and part-time staff numbers at baseline

[c] Rate per 242 days occurring between 1 September and 31 May

[d] Estimated Incident Rate Ratio, adjusted for care home size, nursing care provision, deprivation tertile, and winter.

© 2026 Hay AD et al. *JAMA Intern Med*.

eTable 13. Description of turnover[a]

	Intervention	Control	Overall
Number of care homes	44	47	91
Total number of consented residents	577	592	1169
Residents in withdrawn care homes	2 (0.3%)	0 (0%)	2 (0.2%)
Resident opted-out	7 (1.2%)	1 (0.2%)	8 (0.7%)
Resident left care home	14 (2.4%)	26 (4.4%)	40 (3.4%)
Resident died	101 (17.5%)	105 (17.7%)	206 (17.6%)
Resident consented, but no daily data collected	0 (0%)	1 (0.2%)	1 (0.1%)
Residents active at end of winter	453 (78.5%)	459 (77.5%)	912 (78%)

[a] Some consented residents were not included in analysis due to not having any valid days of data collection

© 2026 Hay AD et al. *JAMA Intern Med*.

eTable 14. Primary and secondary outcomes presented per 1000 resident days: bedroom residents

	Intervention group			Control group			
	N[a]	Resident days[b]	Rate per 1000 resident days[c]	N[a]	Resident days[b]	Rate per 1000 resident days[c]	Adjusted
Primary outcome: number of resident respiratory infection episodes	390	95235	4.10	442	102579	4.31	0.92
Days with respiratory infection symptoms[e]	1416	94666	14.87	1643	101990	16.02	0.77
Episodes of fever and/or delirium and/or acute deterioration in physical ability[e]	281	96139	2.92	309	103679	2.98	0.85
Days with fever and/or delirium and/or acute deterioration in physical ability[e]	774	96139	8.05	857	103679	8.27	0.80
Episodes of gastro-intestinal infection[e]	61	97118	0.63	48	104877	0.46	1.28
Days with gastro-intestinal infection symptoms[e]	167	97118	1.72	103	104877	0.98	1.22
Antibiotic courses prescribed[f g]	572	89567	6.39	599	98322	6.09	1.14
Days antibiotics taken[e]	2067	92924	22.24	2305	100298	22.98	0.69
Number of falls / near-falls[e]	281	95349	2.95	422	102904	4.10	0.64
Episodes of possible SARS-CoV-2-infection[e f]	191	96127	1.99	245	103563	0.57	0.81
Episodes of possible influenza-like-illness[e g]	14	93099	0.15	23	102054	0.23	0.55
PCR confirmed SARS-CoV-2 infections[h j]	31	96834	0.32	19	104485	0.18	1.70
PCR confirmed Influenza A&B infections[h j]	6	96834	0.06	7	104485	0.07	0.98
Microbiologically confirmed care home infection outbreaks[h j k]	8	96834	0.08	8	104485	0.08	0.97
GP diagnosed respiratory, gastrointestinal, skin and urinary infections[h i]	655	89567	7.31	640	98386	6.50	1.21
Hospitalisations: all causes[h i]	322	89567	3.60	320	96309	3.32	1.15

© 2026 Hay AD et al. *JAMA Intern Med.*

[a] Number of outcome events
[b] Number of follow-up days
[c] Number of outcome events per 1000-resident days
[d] Incident rate ratio, adjusted for nursing care provision, deprivation tertile, and winter
[e] Rate determined using 'risk' days, defined as the number of valid days, minus the number of episode days, minus two days (that define episode end), plus one person day to ensure risk >0.
[f] Using UKHSA 'possible' COVID case definition: new continuous cough OR temperature ≥37.8°C OR loss of, or change in, normal sense of smell or taste
[g] Using UKHSA 'possible' influenza-like-illness case definition: temperature ≥37.8°C AND one of: acute onset of at least one of the following respiratory symptoms: cough (with or without sputum), hoarseness, nasal discharge or congestion, shortness of breath, sore throat, wheezing, sneezing) OR an acute deterioration in physical or mental ability without other known cause
[h] Rate determined using 'valid' days defined as the resident follow-up days with daily data collection, excluding days when a resident's data is not required, or a resident is not in the care home, e.g. hospitalized
[i] Data collected from GP medical records
[j] Data provided by UKHSA
[k] Streptococcal, Meningococcal, Respiratory Syncytial Virus, Norovirus and Human Metapneumovirus infections
[l] Defined as respiratory tract infections (+/- sepsis); urinary tract infections (+/- sepsis); skin and soft tissue infections (+/- sepsis); sepsis (no focus identified); trauma (+/- fracture); and major adverse cardiovascular events (MACE). MACE will be further categorized into: arterial (myocardial infarct, angina, new/exacerbation heart failure, transient ischaemic attack, haemorrhagic or thrombotic cerebrovascular accident) and venous (pulmonary embolism or deep vein thrombosis of the upper or lower limb).

© 2026 Hay AD et al. *JAMA Intern Med*.

eReferences

1. Lee GM, Salomon JA, Friedman JF, et al. Illness transmission in the home: a possible role for alcohol-based hand gels. *Pediatrics*. Apr 2005;115(4):852-60. doi:10.1542/peds.2004-0856
2. Little P, Stuart B, Hobbs FD, et al. An internet-delivered handwashing intervention to modify influenza-like illness and respiratory infection transmission (PRIMIT): a primary care randomised trial. *Lancet*. Aug 6 2015;doi:10.1016/S0140-6736(15)60127-1
3. Butler CC, Lau M, Gillespie D, et al. Effect of Probiotic Use on Antibiotic Administration Among Care Home Residents: A Randomized Clinical Trial. *JAMA*. Jul 7 2020;324(1):47-56. doi:10.1001/jama.2020.8556

© 2026 Hay AD et al. *JAMA Intern Med*.
