# Supplementary material for: High-Efficiency Particulate Air Filters to Prevent Winter Respiratory Infections in Care Homes: The AFRI-c Cluster Randomized Clinical Trial
Source: JAMA Intern Med. 2026 Jul 27:e262199. Online ahead of print. doi: 10.1001/jamainternmed.2026.2199 (PMC13409133; doi:10.1001/jamainternmed.2026.2199)
Supplement: Supplement 3. — Data Sharing Statement [file jamainternmed-e262199-s003.pdf]

# Data Sharing Statement

Hay. High-Efficiency Particulate Air Filters to Prevent Winter Respiratory Infections in Care Homes. *JAMA Intern Med*. Published July 27, 2026. doi:10.1001/jamainternmed.2026.2199

**Data**
**Additional Information:** ISRCTN: 63437172
**Data available:** Yes
**Data types:** Deidentified participant data
**How to access data:** https://data.bris.ac.uk/data/dataset/250w5vo8i6he32dc6y3plrq8d9
**When available:** With publication

**Supporting Documents**
**Document types:** Statistical/analytic code
**How to access documents:** https://data.bris.ac.uk/data/dataset/250w5vo8i6he32dc6y3plrq8d9
**When available:** With publication

**Additional Information**
**Who can access the data:** De-identified quantitative and qualitative care home, air filter and participant data collected as part of the randomized trial (including data collected from a review of participant GP notes and provided by the UKHSA), information sheets, template consent forms, interview topic guides, data dictionaries and statistical code are available through the data.bris service (https://data.bris.ac.uk/data/dataset/250w5vo8i6he32dc6y3plrq8d9). In accordance with participant consent, a metadata record describing the Restricted dataset is available through the data.bris repository but the actual data is only made available to authenticated researchers upon application. The applicant must have ethical approval in place to access the data and the applicant's host institution will need to sign a Data Access Agreement
**Types of analyses:** Specified purpose
**Mechanisms of data availability:** De-identified quantitative and qualitative care home, air filter and participant data collected as part of the randomized trial (including data collected from a review of participant GP notes and provided by the UKHSA), information sheets, template consent forms, interview topic guides, data dictionaries and statistical code are available through the data.bris service (https://data.bris.ac.uk/data/dataset/250w5vo8i6he32dc6y3plrq8d9). In accordance with participant consent, a metadata record describing the Restricted dataset is available through the data.bris repository but the actual data is only made available to authenticated researchers upon application. The applicant must have ethical approval in place to access the data and the applicant's host institution will need to sign a Data Access Agreement
**Any additional restrictions:** De-identified quantitative and qualitative care home, air filter and participant data collected as part of the randomized trial (including data collected from a review of participant GP notes and provided by the UKHSA), information sheets, template consent forms, interview topic guides, data dictionaries and statistical code are available through the data.bris service (https://data.bris.ac.uk/data/dataset/250w5vo8i6he32dc6y3plrq8d9). In accordance with participant consent, a metadata record describing the Restricted dataset is available through the data.bris repository but the actual data is only made available to authenticated researchers upon application. The applicant must have ethical approval in place to access the data and the applicant's host institution will need to sign a Data Access Agreement
